# LigninGraphs: lignin structure determination with multiscale graph modeling

**DOI:** 10.1186/s13321-022-00627-2

**Published:** 2022-07-06

**Authors:** Yifan Wang, Jake Kalscheur, Elvis Ebikade, Qiang Li, Dionisios G. Vlachos

**Affiliations:** 1 Department of Chemical and Biomolecular Engineering, University of Delaware, 150 Academy St, Newark, DE 19716 USA; 2 Catalysis Center for Energy Innovation, RAPID Manufacturing Institute, and Delaware Energy Institute (DEI), University of Delaware, 221 Academy St, Newark, DE 19716 USA

**Keywords:** Lignin, Biomass, Structure optimization, Stochastic simulations, NMR spectroscopy

## Abstract

Lignin is an aromatic biopolymer found in ubiquitous sources of woody biomass. Designing and optimizing lignin valorization processes requires a fundamental understanding of lignin structures. Experimental characterization techniques, such as 2D-heteronuclear single quantum coherence (HSQC) nuclear magnetic resonance (NMR) spectra, could elucidate the global properties of the polymer molecules. Computer models could extend the resolution of experiments by representing structures at the molecular and atomistic scales. We introduce a graph-based multiscale modeling framework for lignin structure generation and visualization. The framework employs accelerated rejection-free polymerization and hierarchical Metropolis Monte Carlo optimization algorithms. We obtain structure libraries for various lignin feedstocks based on literature and new experimental NMR data for poplar wood, pinewood, and herbaceous lignin. The framework could guide researchers towards feasible lignin structures, efficient space exploration, and future kinetics modeling. Its software implementation in Python, LigninGraphs, is open-source and available on GitHub.

**Graphical abstract:**

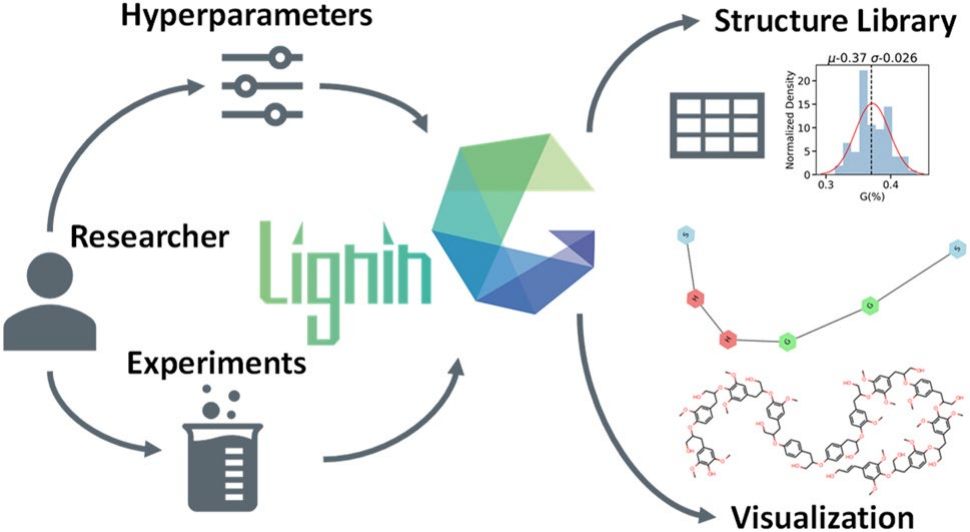

**Supplementary Information:**

The online version contains supplementary material available at 10.1186/s13321-022-00627-2.

## Introduction

Lignocellulosic biomass is a promising and broadly available feedstock for the production of biofuels and renewable chemicals and materials. Lignin, an aromatic polymer, constitutes a significant fraction of lignocellulosic biomass (up to 30 wt%) and is crucial to plant life functioning and structural stability [1, 2]. Despite its abundance in nature and the vast amount generated in the pulp and paper industry, lignin is either unused or burned for heat. Depolymerization techniques, such as reductive catalytic fractionation (RCF), cleave C-O bonds to produce monomers as building blocks for biofuels, functional polymers [2–4], lubricants [5], etc. High-temperature pyrolysis [6, 7] could further break the C–C bonds and increase the monomer yield. However, strong C–C bonds and reactive functional groups in lignin make deconstruction challenging and result in low yields of monomers [8]. To enhance its valorization and produce “designer” lignin with specific properties, we need to understand the lignin structure of various feedstocks better and establish quantitative structure–property relations [7, 9, 10].

**Fig. 1:**
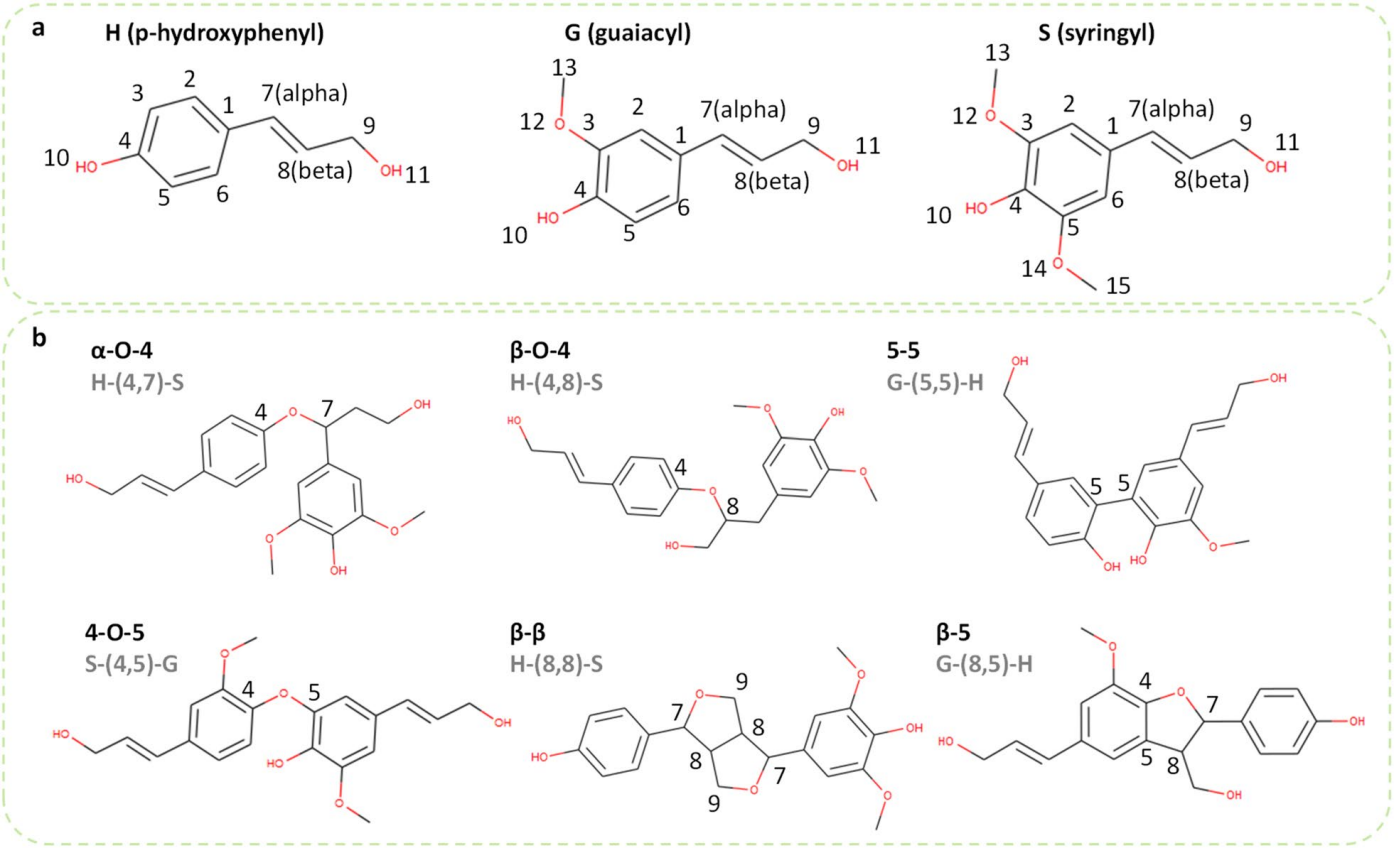
Common monomer units and linkages in lignin. **a** Monomer (monolignol) structures. The numbers are the atomic indices in a monomer (α = ^7^th C and β = ^8^th C). **b** Examples of dimmer structures connected by linkages. The numbers are the atomic indices of the bonding atoms. A linkage between two units is written as the M1-(C1,C2)-M2 (M1 = the type of the first monomer, M2 = the type of the second monomer, C1 = atomic index of the bonding C in the first monomer, C2 = atomic index of the bonding C in the second monomer). For instance, H-(4,7)-S represents an α-O-4 linkage between an H and an S monomer

Lignin’s structural complexity stems from its compositional diversity [11]. As shown in Fig. 1a, phenolics, including p-hydroxyphenyl (H), syringyl (S), guaiacyl (G), and the newly discovered caffeyl alcohol (C-unit) [12–14] in catechyl lignin are the basic lignin monomers (monolignols) [15]. They are coupled and cross-linked combinatorically via various C–O and C–C intramolecular bonds (i.e., linkages, Fig. 1b). These entail α-O-4, β-O-4, 5-5, 4-O-5, β-β, and β-5 [16]. This diversity in monomers and linkages gives rise to compositional variation in each polymer chain. Lignin is polydisperse containing polymers of unequal lengths and branches, leading to a distribution of a complex structural network (i.e., a hyperbranched topology) [17, 18]. Spectroscopic methods, such as nuclear magnetic resonance (NMR) and infrared spectroscopy (IR), can provide compositional distributions on monomers, linkages, and functional groups [3, 19–21]. However, interpretation of spectroscopic data and peak assignment often cause errors. Lignin structures follow mechanistic and chemical principles rather than being a random collection of monomers and linkers, rending the generation of the molecular and atomistic scale structures a non-trivial task.

Significant progress has been made toward computational reconstruction of the lignin structure. One approach builds structural models using the reverse Monte Carlo [22, 23] method to match experimental properties. Examples include an earlier model, SIMREL [24], which adds monomers to growing lignin chains, and the Metropolis Monte Carlo-based methods [17, 25] developed by Broadbelt and co-workers. They generated structure libraries for herbaceous, softwood, and hardwood lignin that satisfy the experimentally measured monomer, linkage, and molecular weight distributions. These models denote structures with tables of monomer indices and sample high-dimensional decision trees to enforce the bonding rules. As a result, the optimization requires many iterations (~ 10^7^) to converge and is slow. Another approach focuses on the structure formation (or dissociation) energies using first-principles density functional theory (DFT) calculations [16, 26, 27]. Yet, DFT calculations can be performed on a limited number of small model compounds that constitute a narrow subset of possible radical species. The computational cost to enumerate all bonds among the species is prohibitively high. A third approach applies hybrid models that estimate energetics from correlations obtained from first-principles data. For instance, Li et al. constructed a database of 4100 possible species that could occur in lignin pyrolysis and estimated the thermochemistry using DFT, group additivity, and machine learning [28]. Orella et al. developed the Lignin-KMC software to model the reactions of S and G monomers on a growing chain using the estimated linkage energies [29, 30]. Lignin-KMC employs graph theory and represents structures as adjacency matrices consisting of indices for bonding atoms in each monomer, which may not be intuitive for non-computational researchers. Atomistic representation becomes computationally intensive as the degree of polymerization increases.

Determining the lignin structure needs a rapid reconstruction method to satisfy experimental observables and encode first-principles information for follow-up kinetic studies. In this work, we design a reconstruction model and LigninGraphs, a lightweight software tool in Python, for speed and accuracy. LigninGraphs has two important methodological innovations. First, it encodes the lignin structures in multiscale graphs over multiple length scales and supports graph and molecular structure visualization. Second, unlike the traditional algorithm, it introduces a rejection-free Metropolis Monte Carlo method that keeps track of bonding atoms, accelerating convergence significantly. As a result, the candidate structures can be generated quickly, saved in smiles strings or graphs, and their experimental observables can be computed. Furthermore, LigningGraphs can be interfaced easily with other tools to calculate the energetics using group additivity [28] or render 3D structures using the LigninBuilder [31]. LigninGraphs is an open-source software with comprehensive online documentation. We apply it to generate structure libraries for poplar, pinewood, and herbaceous lignin (miscanthus) NMR data.

## Computational methods

### Overview

**Fig. 2:**
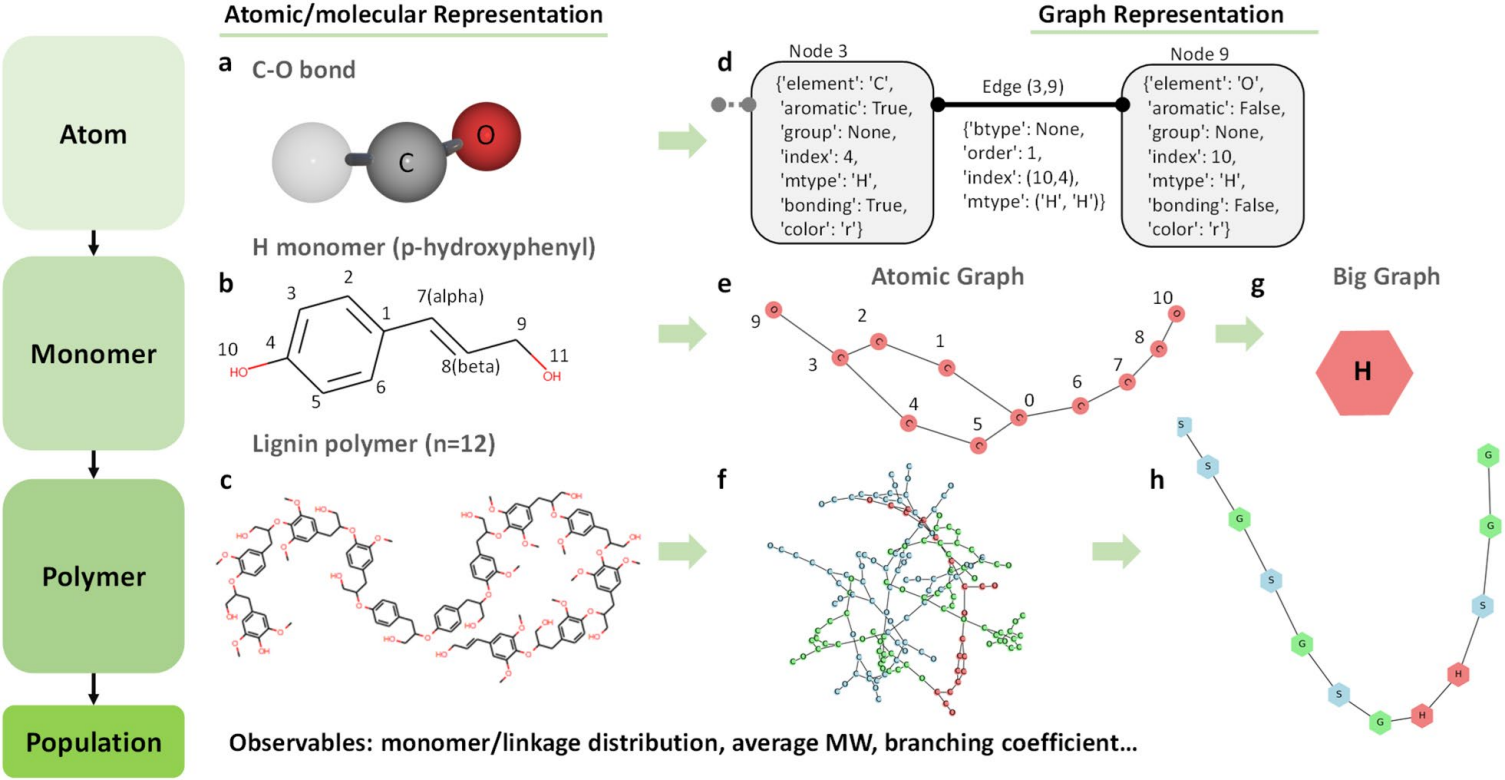
Multiscale modeling framework in LigninGraphs. Lignin structures are modeled at multiple scales (atom, monomer, polymer, and population). The left column shows the atomic or molecular representations for **a** a C–O bond, **b** an H monomer with C and O atom indices, and **c** a lignin polymer of 12 monomers. The right column shows the corresponding structures as atomic graphs (**d**–**f**) and big (coarse-grained) graphs (**g**, **h**). **d** Building blocks in atomic graphs: each C or O atom is a node, and each bond is an edge in the graph with properties saved in a dictionary format. **e** Atomic graph for an H monomer with node index for each C or O atom (note that the node index starts from 0 and the atom index starts from 1, i.e., the node index is equal to the corresponding atom index minus 1). In this example, node 3 is the 4th C atom and node 9 is the 10th O atom in an H monomer. They are connected by a single bond (order = 1) edge (3, 9). **f** Atomic graph for the lignin polymer in (**c**). **g** Big graph for an H monomer. **h** Big graph for the lignin polymer in (**c**): each monomer is a node, and each linkage is an edge. H, G, and S monomers and the atoms belonging to each type are marked in red, green, and blue, respectively

Our framework (Fig. 2) models lignin structures across scales, from the atomistic to the monomer to the polymer to a population of chains (i.e., a library of structures). We map structures to undirected graphs consisting of a list of nodes and connections (edges) between nodes, as in traditional graph theory. Graphs offer efficient data storage, fast lookup operations, and intuitive visualization for each node and its neighbors as an abstract data structure. At the atomic scale, each C or O atom is a node, and each bond is an edge (Fig. 2a, b). Atom properties are conveniently stored in a node. These include the element (“element”: C or O), the presence of an aromatic ring (“aromatic”: True or False), the monomer type (“mtype”: H, G, or S), the atomic index (“index”: the numeric labels for each C/O atom in a monomer, shown in Fig. 1a), bonding (“bonding”: True or False), etc. Similarly, bond properties are stored in an edge. These include the linkage type (“btype”: the common ones, α-O-4, β-O-4, 5-5, 4-O-5, β-β, and β-5, are shown in Fig. 1b) or None for intra-monomer bonds), the bond order (“order”: 1 for a single or 2 for a double bond), atomic indices (“index”: atomic indices of the pair), and the monomer types of the bonding atoms (“mtype” of the pair). The resulting “atomic graphs” represent structures at the monomer (Fig. 1e) and the polymer (Fig. 1f) scale.

Unlike the typical graph theory that resolves each atom and bond individually, we introduce a multiscale representation of the structure by coarse-graining upon molecular structures. Specifically, we coarse-grain the atomic graphs into “big graphs” by aggregating all nodes in a monomer into a single node (Fig. 2h), so that each monomer is a node and each linkage is an edge (examples of building blocks are shown in Additional file 1: Fig. S1). Big graphs are connected at the polymer scale to make up a chain. They allow a significantly lower computational cost and storage by counting only the number of monomers or linkages. Finally, at the population scale of the polymer, we compute the observables or structure metrics of the entire structure library, including the monomer and linkage distributions, the number average molecular weight (MW), and the branching coefficient. These observables can be compared to experimental values.

LigninGraphs offers multiple ways for visualization. The atomic graphs and big graphs can be plotted directly. The atomic graphs can also be easily converted to molecular structures, shown in Fig. 1b/c leveraging RDKit, or 3D structures, leveraging LigninBuilder [31]. We convert the framework to an open-source package, named LigninGraphs, written in Python. We provide the implementation details in Additional file 1: Note 1 and on the online documentation.

### Monomers and linkages

**Fig. 3:**
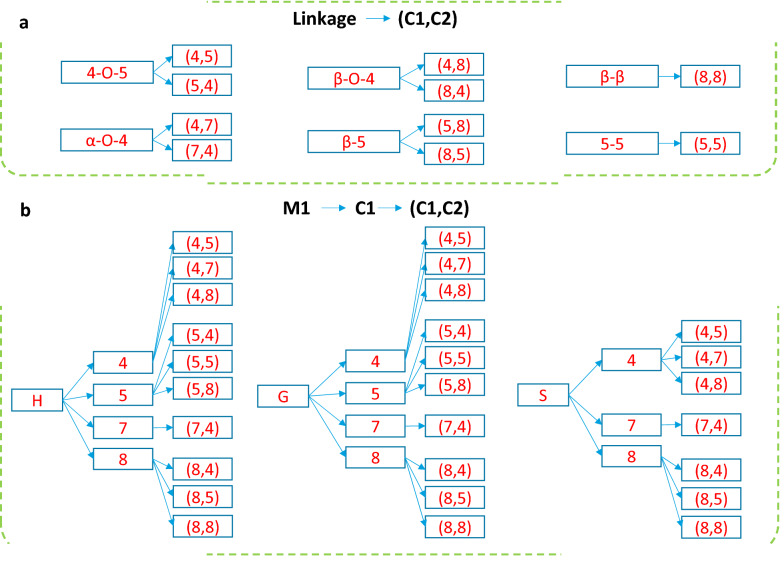
Rules for mapping monomer and linkage types to atomic index pairs in LigninGraphs. **a** Linkage types to atomic index pairs (C1,C2). **b** Types of monomer (M1) and feasible C atom indices (C1) and atomic index pairs (C1,C2) for bond formation between monomers M1 and M2

Figure 1a shows the three most common monomer units in lignin, p-hydroxyphenyl (H), syringyl (S), and guaiacyl (G). We assign an atomic index to each C and O atom in a monomer following the standard convention. Such index information is useful to correctly bond atoms as linkages in graph representations. Figure 1b shows example dimmers consisting of the six common linkages, namely α-O-4, β-O-4, 5-5, 4-O-5, β-β, and β-5. Depending on the bonding atoms, linkages are written as a pair of atomic indices. For instance, an α-O-4 linkage corresponds to (4,7) or (7,4) pair of atoms. Figure 3a shows the mappings for all types. Except for 5-5, all other linkages involve neighboring C or O atoms in bond formation. We implement rules to break and make the correct bonds based on the atomic indices.

### Polymerization

We model the growth of a single lignin polymer chain via a reconstruction process. Starting from a monomer, we add monomers one at a time with different linkages. Connecting a monomer M1 of a growing polymer to a monomer M2 with the correct linkages requires identifying the bonding atoms' possible atomic index pairs, i.e., the (C1,C2) pairs. C1 refers to the bonding C atom index in M1 (in a polymer) and C2 to M2 (a new monomer for each addition). Therefore, we list the mapping rules for each monomer or linkage-type to atomic index pairs (Fig. 3), and store them in Python dictionaries (hash maps) to achieve fast lookup operations at a constant time, of order 1, $O(1)$.

To add a specific linkage or monomer, we obtain the possible atomic index pairs and C1s via the mapping rules. We also keep a list of available bonding atoms and their atomic indices (i.e., the C1s) in the growing polymer. We quickly narrow down the suitable C1 atoms by taking the intersections of the two C1 lists. Next, one of the C1s is selected randomly and connected with the corresponding C2. From a C2 index, one could use the reverse mapping in Fig. 3b and determine the correct monomer type for the addition (C2 → M2). A new edge in the graph is connected, and its “bonding” properties are marked as False. Each addition is essentially a rejection-free Monte Carlo event, i.e., the proposed monomer and linkage are always added to a valid structure. We achieve a linear time ($O(n)$, where n is the polymer size, for each addition and eliminate unsuccessful attempts. Users may add a specific monomer, linkage (e.g., a ring formation with no monomer), or ring, and a random monomer or ring. The rules ensure that the structures are always chemically valid. The addition is still subject to the Metropolis acceptance criterion if we want to create a structure library like the experimental data.

### Characterization

Given a wealth of chemical information stored in graphs, we quickly compute the structure metrics of a polymer molecule. Such metrics include the number of methoxy groups (–OCH_3_), phenolic hydroxy groups (–OH), monomers and linkages of each type, MW, the size (total number of monomers), and the branching coefficient. We follow the definition of branching coefficient in Dellon et al., i.e., the ratio of branched monomers to the size of a branched monomer connecting to three or more monomers [25]. For a population of structures, the probability distribution function and global metrics, such as the percent of a monomer, linkage, and the branching coefficient, are computed.

### Optimization

**Fig. 4:**
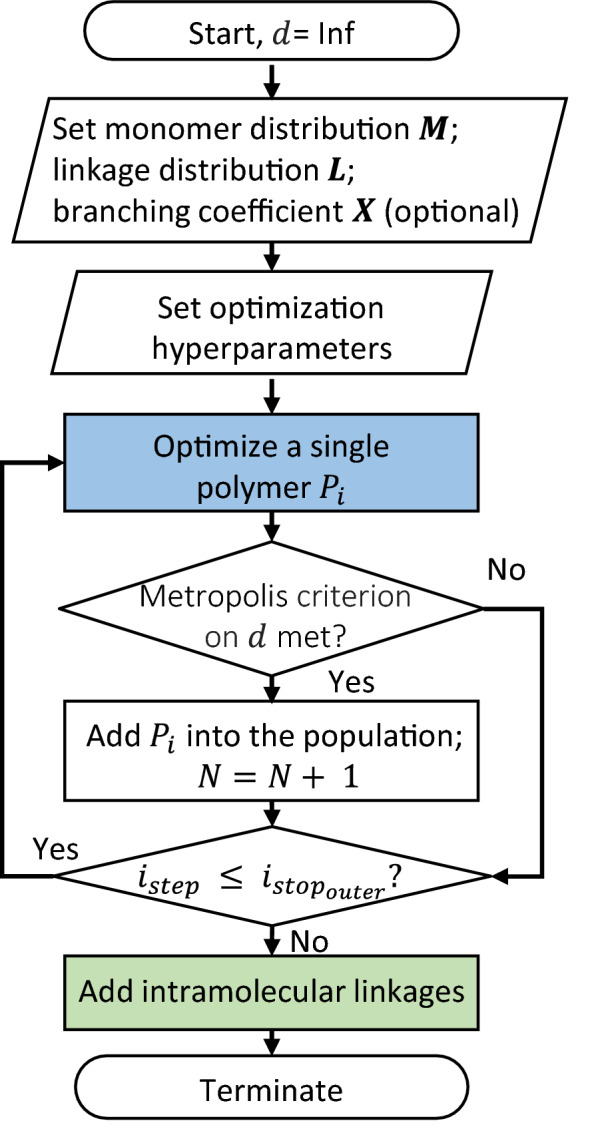
Hierarchical optimization scheme in LigninGraphs

Finally, we utilize a Metropolis Monte Carlo algorithm to obtain a library of structures and global metrics to compare to target experimental data. The hierarchical scheme (Fig. 4) optimizes the molecular and population structures and refines them with intramolecular linkages. We define a single distance metric ${d}_{i}$ for a polymer molecule ${P}_{i}$ as the sum of squared errors:

1 $${d}_{i}= {\sum }_{M\in \{H, G, S\}}({{M}_{exp}-{{M}_{i}}_{sim})}^{2}+{\sum }_{L\in \{6 types of linkages\}}({{L}_{exp}-{{L}_{i}}_{sim})}^{2}+\Sigma ({{X}_{exp}-{{X}_{i}}_{sim})}^{2}$$

Here, $M$, $L$, $X$ are the monomer percentages, linkage percentages, and additional metrics, such as the branching coefficient, respectively, $exp$ denotes the experimental values and $sim$ simulated values. For a population, the distance $d$ applies to the global $M$ s, $L$ s, $\mathrm{and} X$ s:

2 $$d= {\sum }_{M\in \{H, G, S\}}({{M}_{exp}-{M}_{sim})}^{2}+{\sum }_{L\in \{6 types of linkages\}}({{L}_{exp}-{L}_{sim})}^{2}+\Sigma ({{X}_{exp}-{X}_{sim})}^{2}+\Sigma \frac{({{\overline{MW} }_{exp}-{\overline{MW} }_{sim})}^{2}}{M{W}_{max}^{2}}$$

Here, we included the number average MW of the population (${\overline{MW} }_{sim}$) if the experimental value (${\overline{MW} }_{exp}$) is available. To ensure a similar scale ([0-1]), we divide the MW by the upper bound ($M{W}_{max}$, set as 10,000 Da). The optimization minimizes $d$, i.e., makes the simulated population as similar to the experiments as possible.

We first optimize a single polymer molecule by repeatedly adding monomers (linkages), as shown in the inner optimization loop in Additional file 1: Fig. S4. The monomer type, the linkage type, or the branching state for addition are sampled from the experimental distributions, following the rules described in the Polymerization section (Additional file 1: Fig. S5). For instance, for an experimental G:S ratio of 6:4 with no H present, the probability of adding a G and S monomer is 60% and 40%, respectively. Each event is accepted depending on the Metropolis criterion using $\Delta {d}_{i}$ (Additional file 1: Note 2). The polymer growth stops when the number of iterations or the polymer size is reached. The latter is sampled from a normal distribution and is expressed by the MW (${{MW}_{i}}_{stop}$, if the experimental value is available) or the number of monomers (${{n}_{i}}_{stop}$) (Additional file 1: Table S1). Next, we optimize the entire population (Fig. 4), shown as the outer optimization loop in Additional file 1: Fig. S4. Optimized polymers from the inner loop are added one at a time, based on the Metropolis criterion using $\Delta d$. Once 100 molecules have been added, or the maximum number of iterations is reached, we fine-tune linkages distributions by adding possible rings as intramolecular linkages (optimization loop in Additional file 1: Fig. S4). Several hyperparameters in the framework, such as the Metropolis temperature, the branching propensity, and the expected polymer size, can be tuned to speed up convergence. A complete list can be found in Additional file 1: Table S2.

## Experimental section

### Materials

The poplar and pine samples were obtained from the Idaho National Laboratory [32] and characterized using the NREL LAP protocols [33]. The samples were milled to particles ranging from 0.42 mm (40 mesh) to 2 mm (10 mesh) by Forest Concepts and used as received. The monomer content was determined using the thioacidolysis method [34] described in Ebikade et al. [35].

### Nuclear magnetic resonance (NMR)

Heteronuclear single quantum coherence (HSQC) nuclear magnetic resonance (NMR) spectra of extracted lignin oils were recorded at 25 °C on an Avance III 400 MHz NMR spectrometer (Bruker). Approximately 30 mg of filtered lignin oil was dissolved in 500 µl of premixed DMSO-d6/pyridine-d5 (4: 1) prepared in quartz NMR tubes (NewEra). Data was processed using the Mestrelab Research software (mNOVA). The NMR 2D spectra show contours of specific bonds between protons (^1^H) and carbon (^13^C) atoms. The relative abundance of chemical functionalities was performed by integrating the volume of ^1^H–13C cross-peaks and normalizing such volumes by the total integrated volumes of the monolignols. The calculation details from the HSQC experiments can be found in the previous work [35].

## Results and discussion

### Structure library generation

**Fig. 5:**
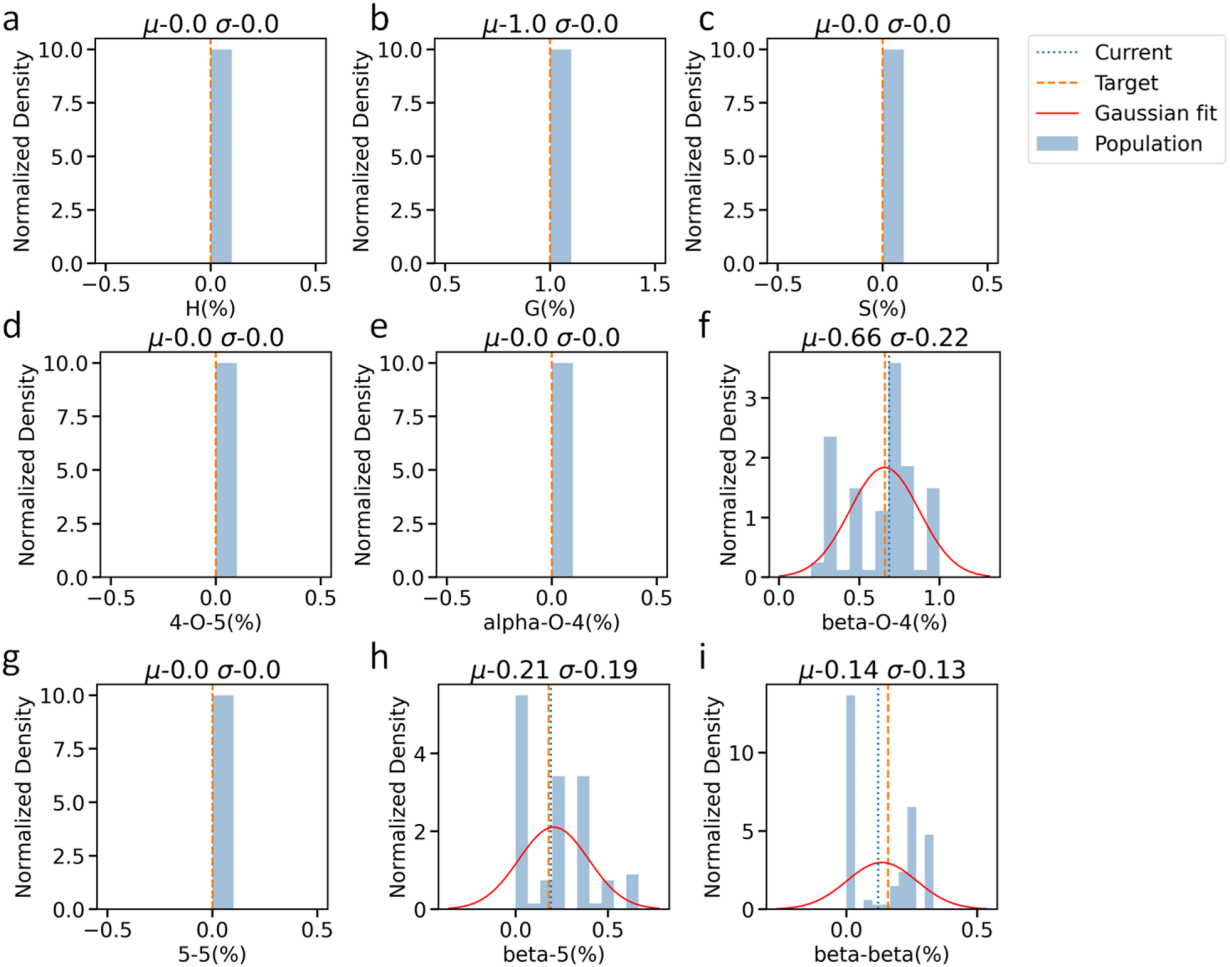
Pine lignin structure metrics of simulated optima and experimental (target) values. Monomer percentages: **a** H, **b** G, **c** S. Linkage distributions: **d** 4-O-5, **e** α-O-4, **f** β-O-4, **g** 5–5, **h** β-5, and **i** β-β. The means (µ) and standard deviations (σ) of the population are shown at the top of each subfigure

To demonstrate the effectiveness of LigninGraphs, we generate structure libraries for three lignin feedstocks: pine, poplar, and miscanthus. The linkage and monomer distributions for pine and poplar are determined by the procedure laid in the Experimental section. For miscanthus, a linear herbaceous lignin, we obtain the distributions from 2D HSQC NMR data, and the molecular weights from size exclusion chromatography reported in the literature [25, 34]. The experimental and simulated values are listed in Table 1. Pine and poplar are hardwood and softwood, respectively. In general, the composition of β-O-4 linkage in hardwood is 10–15% less than it in softwood [36] and our experiment and simulated results are within this range with differences of 12 and 10.7%, respectively. LigninGraphs also generates histograms of the population and a Gaussian distribution fit. Figure 5, Additional file 1: Figs. S6, and S7 show the distributions for poplar, pine, and miscanthus, respectively. The structure library, including the metrics and smiles strings of 100 polymer molecules, is given in Table S3-5. We chose the population size as 100 because it is statistically significant, and the simulations would finish in a reasonable time. Hydroxyl groups, measured by ^31^P NMR [20], can also be simulated using LigninGraphs (not demonstrated in this work).

**Table 1:** Target versus simulated values of lignin structure for pine, poplar and miscanthus

Feedstock	Pine		Poplar		Miscanthus	
Metrics	Experiments (this work)	Simulated	Experiments (this work)	Simulated	Experiments (literature [25, 34])	Simulated
H (%)	0	0	0	0.00	4	3.2
G (%)	100	100	37	37.7	46	48.0
S (%)	0	0	63	62.3	50	48.8
4-O-5 (%)	0	0	0	0	0	0
α-O-4 (%)	0	0	0	0	0	0
β-O-4 (%)	66	68.7	78	79.4	68	73.2
5-5 (%)	0.1	0	0.1	4.9	0	0
β-5 (%)	18	19.2	7	4.2	15	14.2
β-β (%)	16	12.1	15	11.7	17	12.6
APS^a^		5.5		4.7		6.6
BC^b^		0		0.02	0	0.0
N_MW_(Da)^c^		986		928	1240	1272

^a^Average polymer size (no. monomers)

^b^Branching coefficient

^c^Number of average MW

The monomer distributions closely match the experiments. For pine (Fig. 5), we have G units only. β-O-4 linkages dominate, and the fractions of β-5 and β-β are significant. The experiments for pine and poplar detect a small fraction (< 0.1%) of 5-5. For poplar, the simulations often assign a higher percentage to 5-5 (less than 5%) and lower percentages to β-β or β-5. We find that having a fraction of 5-5 is essential to minimizing the overall distance. Otherwise, a larger percentage of β-O-4 would be needed.

Pine and poplar lignin structures could be branched. With a nonzero branching propensity, the simulations attempt adding monomers to branched locations while still prioritizing matching other metrics. We observe a minor effect of the branching propensity on the branching coefficient of the resulting libraries (Additional file 1: Fig. S8a). The branching coefficients in the optimal structures for pine and poplar are 0 and 0.02, respectively. It is possible that the degree of branching is insignificant in both feedstocks. We also observe a strong positive correlation (${r}_{pearson}=0.95$) between the 5-5 linkage percentage and the branching coefficient (Additional file 1: Fig. S8b), indicating the 5-5 linkage could encourage branching. Such observation is consistent with the finding of Broadbelt and co-workers. [25] Other linkages, such as β-β or β-5, show a weak negative correlation with the branching coefficient (Additional file 1: Fig. S8b). The experiments have a margin of error in the linkage distributions up to 5% [25]. The extraction and characterization methods could also contribute to the uncertainty. The simulated statistics can also be improved by running at a lower Metropolis temperature or using more MC events.

### Computational performance

LigninGraphs shows a significant computational improvement compared to prior works. The hash-map-encoded bonding rules, enabled by the multiscale coarse-graining method, ensure a constant time for each lookup operation. We sacrifice a small amount of extra memory to store the list of available bonding C1 atom indices in a growing polymer but achieve a rejection-free addition of monomers and linkages at a linear runtime. As a result, the overall time complexity for polymerization is at a near quadratic time, $\sim O\left({n}^{2}\right)$, faster than the $O\left({n}^{2.5}\right)$ of Lignin-KMC (detailed comparison is shown in Additional file 1: Note 4). In addition, due to the rejection-free scheme, LigninGraphs uses fewer iterations to converge than the conventional Metropolis Monte Carlo-based methods [17, 25]. Prior methods require repeated sampling from a high-dimensional decision tree, employing more than ${10}^{7}$ Monte Carlo attempts in the optimization. In LigninGraphs, a specific monomer/linkage addition is obtained by sampling a simpler version of such a decision tree and is always executed. Depending on the Metropolis temperature, a typical simulation generating a structure library of 100 polymers would finish within ${10}^{5}$ MC attempts in ~ 1–30 min on a Windows 10 laptop with an Intel^(R)^ Core^(^™^)^ i7 processor. LigninGraphs is expected to be at least 100 × more efficient than other simulation methods based on its rejection-free approach and save additional time due to its multiscale graph approach. Table S6 shows the acceptance ratios, total MC attempts, and runtime as a function of the Metropolis temperature from the test simulations on the three feedstocks. The speedup becomes more pronounced at lower temperatures, where the classic Metropolis algorithm results mainly in rejected attempts, and for larger polymers. Multithreading on parallel machines could further increase the computational speed and is a potential direction for a future version of LigninGraphs. Each polymer molecule could be created on one thread concurrently and be aggregated into a population based on the Metropolis acceptance rule.

### Multiscale representation and big SMILES

The multiscale structure representation in LigninGraphs provides a wealth of information spanning from the atomic level to the population level. In general, the graph data structure makes it easy to store property information and perform visualization. Atomic graphs and associated bonding rules serve as a detailed atomic structure generator to eliminate human errors. We can also query the number of functional groups, such as –OCH_3_ and phenolic –OH groups that are unavailable in prior works. The big coarse-grained graphs allow fast characterization of the monomer and linkage distributions. At the population scale, a lignin structure library for a particular feedstock represents a stochastic distribution of polymers. We compute the observable metrics and save the SMILES strings for all polymer molecules. One could relate such a branched stochastic polymer to the BigSMILES notation [37]. BigSMILES can be an indexing tool to distinguish different simple lignin structures: branched vs. linear, S/G lignin vs. G lignin, etc. We include an example in Additional file 1: Fig. S2 to show the BigSMILES notation for hypothetical linear lignin with only G units and β-O-4 linkages. When more linkages or branching are allowed, the notation becomes complex and less comprehensible as the bonding descriptor ([$]) can be added to the 4th, 5th, 7th, or 8th C. BigSMILES alone could not represent structures due to the loss of the polymer size and linkage distributions which are essential in modeling the chemistry and predicting product distributions in the lignin valorization. In such cases, one should utilize the entire structure library.

## Conclusions

We introduce a multiscale graph-based modeling framework to construct lignin structure libraries based on experimental data. The framework represents lignin structures at the atomic, molecular, and population scales and coarse-grains information from scale to scale to save computational and storage needs. The multiscale representation in graphs enforces bonding rules, provides structure visualization, and characterizes observable properties, such as monomer and linkage distributions, functional group counts, molecular weights, and branching coefficients. The hierarchical Metropolis Monte Carlo optimization scheme guides structure generation to match the target experimental values. We introduce rejection-free polymerization and structure optimization algorithms for faster convergence. We illustrate the framework for pine, poplar, and miscanthus lignin with new 2D-HSQC NMR data for the first two obtained herein and from the literature for miscanthus. The branched structures of pine and poplar reveal a positive correlation between branching and the percentage of 5-5 linkage. The software implementation in Python, LigninGraphs, is modular, easy to use, and open-source, with online documentation. Future work could easily extend the current framework by including more types of linkages, such as β-1 (or spiro-dienone) [38, 39], and caffeyl alcohol monomers and associated bonding rules to obtain structures for diverse lignin feedstocks. In addition, more quantitative characterization techniques [40] on linkage distributions can be used to replace 2D HSQC NMR, the same computational approach would still apply. We believe LigninGraphs can be valuable in creating biopolymer and other polymer structures in general. It can be used to model the kinetics of lignin valorization, such as pyrolysis, based on mechanistic principles and chemical knowledge. For instance, one can easily connect it to previous approaches, such as LigninBuilder [31], to unveil thermodynamic properties. The structure library can also provide a feasible pool of candidate radicals for first-principles calculations when simulating radical coupling events in lignin polymerization.

## Supplementary Information

**Additional file 1.** (1) Software design of LigninGraphs, including the key modules and functions, (2) Details of the Metropolis Monte Carlo-based optimization algorithm, (3) Optimal structure metrics and simulated libraries for pine, poplar, and miscanthus lignin, (4) Computational performance. **Figure S1**. Units in big graphs where each monomer is a node and each linkage is an edge. In this example, both nodes 0 and 1 are H monomers, and the edge (0,1) represents a beta-O-4 linkage between the two. **Figure S2**. BigSMILES notation^1^ for a hypothetical linear lignin structure with only G units and β-O-4 linkages. The corresponding atomic indices, as shown in Figure 2a, are marked for each C/O atom. In this case, the bonding descriptor ([$]) indicates that the 4^th^ or the 8^th^ C can be connected to any other atom with the same bonding descriptor. Such linkages can be written as G-(4,8)-G or G-(8,4)-G in the M1-(C1,C2)-M2 format. Following smiles notation, the lower case ‘c’ indicates aromatic ring carbons, and capital case ‘C’ means non-aromatic carbons. **Figure S3**. LigninGraphs software design. (a) High-level workflow and (b) main modules, classes, functions (marked in bold), and their usages. **Figure S4**. Hierarchical optimization scheme in LigninGraphs. **Table S1**. Proposed structure additions, stopping criteria, and return values in each optimization loop. **Figure S5**. Illustration of new monomer addition via random sampling. For instance, the experimental monomer distributions are 0, 0.37, and 0.63 for H, G, S; the linkage distributions are 0, 0, 0.78, 0, 0.07, and 0.15 for 4-O-5, 5-5, α-O-4, β-O-4, β -5, β-β; the branching propensity is 0.25. After random sampling for each property, with random numbers *r* ∈ [0,1] shown in dials, a new G monomer is added via a β-O-4 to a terminal monomer to avoid branching. **Table S2**. List of hyperparameters in the multiscale optimization framework. **Figure S6**. Poplar lignin structure metrics for the simulated optima and target values. Monomer percentages: a, H. b, G. c, S. Linkage distributions: d, 4-O-5. e, α-O-4. f, β-O-4. g, 5-5. h, β-5. i, β-β. The population's means (µ) and standard deviations (σ) are shown at the top of each subfigure. **Figure S7**. Miscanthus lignin structure metrics for the simulated optima and target values. Monomer percentages: a, H. b, G. c, S; Linkage distributions: d, 4-O-5. e, α-O-4. f, β-O-4. g, 5-5. h, β-5. i, β-β. The population's means (µ) and standard deviations (σ) are shown at the top of each subfigure. **Figure S8**. Correlations between (a) branching propensity, (b) 5-5 linkage percentage, (c) β-O-4 linkage percentage, and (d) β- β simulated branching coefficient for pine and poplar simulations. The values of Pearson correlation coefficients ($${r}_{pearson}$$) are indicated. Values close to 1, -1, and 0 suggest a strong positive, negative, and weak correlation, respectively. **Table S3**. Example structure library for pine lignin. The counts of monomers, linkages and functional groups, branching coefficient, and the smiles strings of the structures are shown. **Table S4**. Example structure library for poplar lignin. The counts of monomers, linkages and functional groups, branching coefficient, and the smiles strings of the structures are shown. **Table S5**. Example structure library for miscanthus lignin. The counts of monomers, linkages and functional groups, branching coefficient, and the smiles strings of the structures are shown. **Figure S9**. Computational time complexity of polymerization. For LigninGraphs, blue dots indicate the measured mean CPU execution time for a simulation at a specific polymer size; the blue error bars indicate the 95% confidence interval of the CPU time, averaged over five runs. The blue and orange dashed lines are the power-law fits of the polymerization time complexity for LigninGraphs and Lignin-KMC, respectively. **Figure S10**. Example distance trajectories in the Metropolis Monte Carlo simulations for (a) a structure population and (b) a single polymer. **Table S6**. Acceptance ratios, total Monte Carlo (MC) attempts, and runtime for various lignin feedstocks.

## Data Availability

LigninGraphs is publicly available free of charge at https://github.com/VlachosGroup/ligningraphs. It can be installed using the standard package-management system in Python. The online documentation is available at https://ligningraphs.readthedocs.io/en/latest.
