# Supplementary material for: LigninGraphs: lignin structure determination with multiscale graph modeling
Source: J Cheminform. 2022 Jul 6;14:43. doi: 10.1186/s13321-022-00627-2 (PMC9261032; doi:10.1186/s13321-022-00627-2)
Supplement: Supplementary file 1 — Additional file 1. (1) Software design of LigninGraphs, including the key modules and functions, (2) Details of the Metropolis Monte Carlo-based optimization algorithm, (3) Optimal structure metrics and simulated libraries for pine, poplar, and miscanthus lignin, (4) Computational performance. Figure S1. Units in big graphs where each monomer is a node and each linkage is an edge. In this example, both nodes 0 and 1 are H monomers, and the edge (0,1) represents a beta-O-4 linkage between the two. Figure S2. BigSMILES notation1 for a hypothetical linear lignin structure with only G units and β-O-4 linkages. The corresponding atomic indices, as shown in Figure 2a, are marked for each C/O atom. In this case, the bonding descriptor ([$]) indicates that the 4th or the 8th C can be connected to any other atom with the same bonding descriptor. Such linkages can be written as G-(4,8)-G or G-(8,4)-G in the M1-(C1,C2)-M2 format. Following smiles notation, the lower case ‘c’ indicates aromatic ring carbons, and capital case ‘C’ means non-aromatic carbons. Figure S3. LigninGraphs software design. (a) High-level workflow and (b) main modules, classes, functions (marked in bold), and their usages. Figure S4. Hierarchical optimization scheme in LigninGraphs. Table S1. Proposed structure additions, stopping criteria, and return values in each optimization loop. Figure S5. Illustration of new monomer addition via random sampling. For instance, the experimental monomer distributions are 0, 0.37, and 0.63 for H, G, S; the linkage distributions are 0, 0, 0.78, 0, 0.07, and 0.15 for 4-O-5, 5-5, α-O-4, β-O-4, β -5, β-β; the branching propensity is 0.25. After random sampling for each property, with random numbers r ∈ [0,1] shown in dials, a new G monomer is added via a β-O-4 to a terminal monomer to avoid branching. Table S2. List of hyperparameters in the multiscale optimization framework. Figure S6. Poplar lignin structure metrics for the simulated optima and target values. Monomer per [file 13321_2022_627_MOESM1_ESM.docx]

**Additional Information for**

**LigninGraphs: Lignin Structure Determination with Multiscale Graph Modeling**

Yifan Wang,^1,2^ Jake Kalscheur,^1,2^ Elvis Ebikade,^1,2^ Qiang Li,^2^ and Dionisios G. Vlachos^1,2*^

^1^Department of Chemical and Biomolecular Engineering, 150 Academy St., University of Delaware, Newark, Delaware 19716, United States

^2^Catalysis Center for Energy Innovation, RAPID Manufacturing Institute, and Delaware Energy Institute (DEI), 221 Academy St., University of Delaware, Newark, Delaware 19716, United States

*Corresponding author: [vlachos@udel.edu](mailto:vlachos@udel.edu)

**Additional Figures**

**Figure S1**. Units in big graphs where each monomer is a node and each linkage is an edge. In this example, both nodes 0 and 1 are H monomers, and the edge (0,1) represents a beta-O-4 linkage between the two.


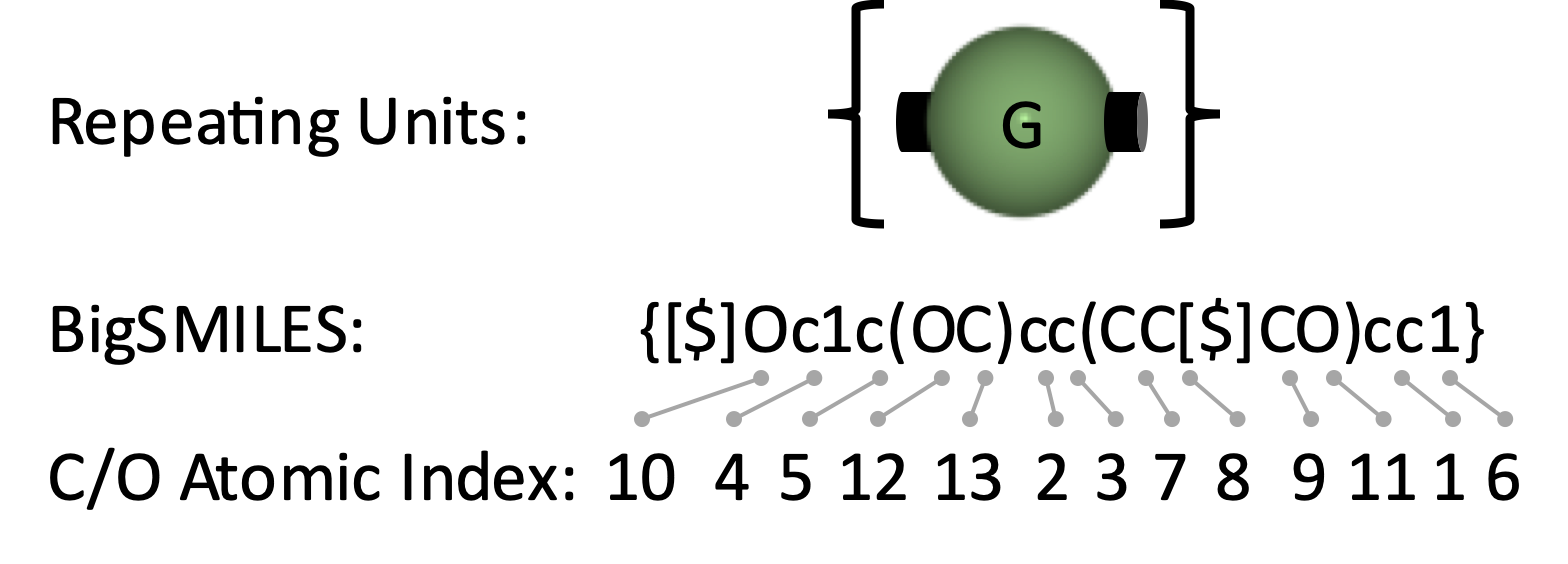


**Figure S2**. BigSMILES notation^1^ for a hypothetical linear lignin structure with only G units and β-O-4 linkages. The corresponding atomic indices, as shown in Figure 2a, are marked for each C/O atom. In this case, the bonding descriptor ([$]) indicates that the 4^th^ or the 8^th^ C can be connected to any other atom with the same bonding descriptor. Such linkages can be written as G-(4,8)-G or G-(8,4)-G in the M1-(C1,C2)-M2 format. Following smiles notation, the lower case ‘c’ indicates aromatic ring carbons, and capital case ‘C’ means non-aromatic carbons.

**Additional Note 1 – LigninGraphs Software Design**

The LigninGraphs software package is structured similarly to the multiscale modeling framework. Given experimental characterization data, one can change the hyperparameters (optional) to perform structure optimization that closely matches the data. LigninGraphs is implemented in a modular fashion. Figure S3b summarizes its core classes, modules, and functions. The main modules, including monomer, polymer, characterization, and optimization, correspond to the sections mentioned in the Methods. The output structure libraries are saved in CSV or Excel format containing each polymer molecule's smiles strings and metric values. The LigninGraphs software also serves as a great visualization tool displaying the structures in graph or chemical form. We provide comprehensive examples on the documentation page to demonstrate its easy implementation and modularity.


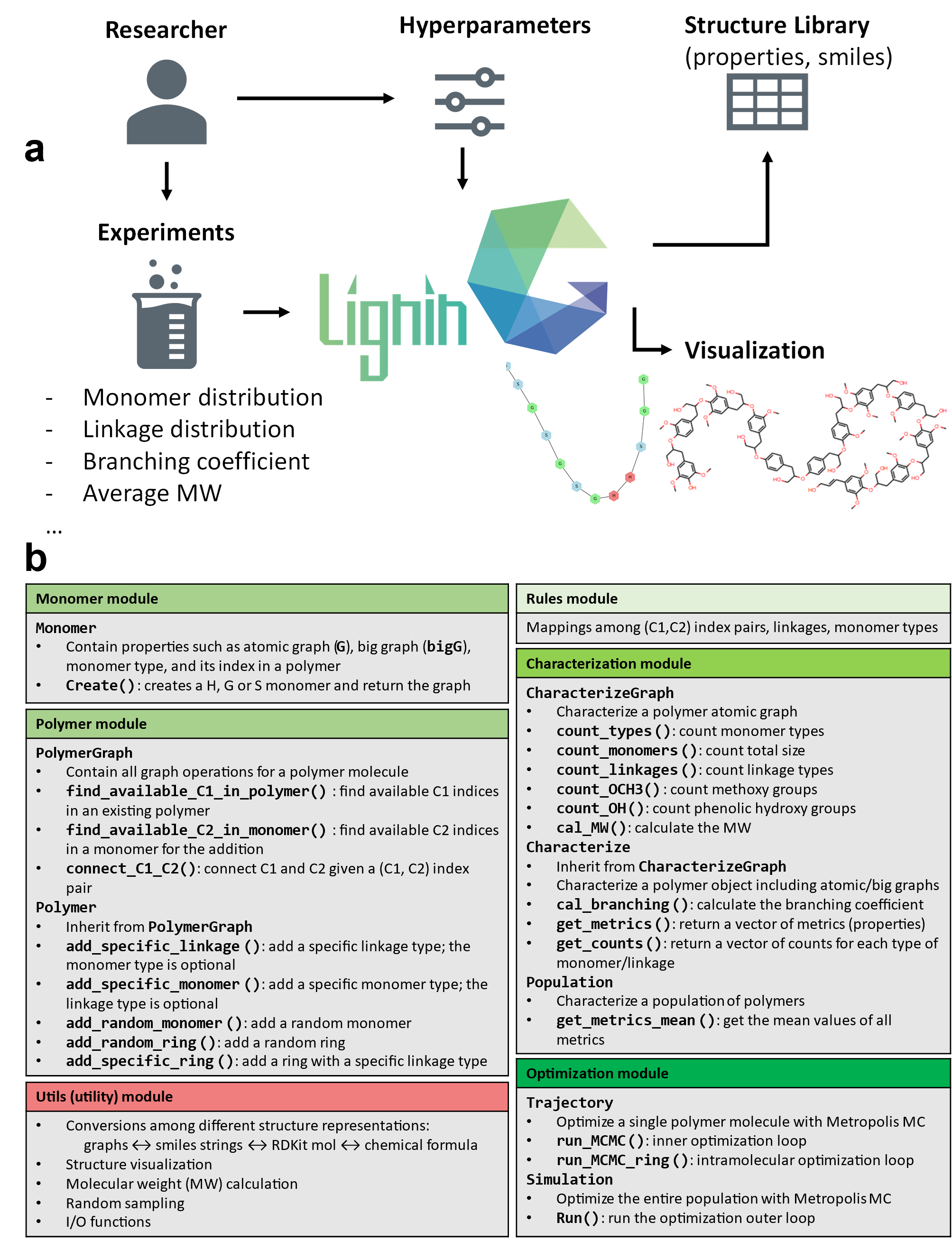


**Figure S3**. LigninGraphs software design. (a) High-level workflow and (b) main modules, classes, functions (marked in bold), and their usages.

**Additional Note 2 – Metropolis Monte Carlo-based Optimization**

**Hierarchical optimization scheme**

We show the extended scheme for hierarchical optimization in Figure S4. The scheme contains three optimization loops: outer loop, inner loop, and intramolecular linkage loop. Table S1 explains the proposed structure additions, stopping criteria, and return values in each loop. We optimize a single polymer molecule $P_{i}$, add it to a population in the outer loop, and then refine the linkage distributions by adding intramolecular linkages. In this case, each polymer molecule and the entire population would have metric values close to the target values. We sample from the target distribution when adding a monomer or a linkage (illustrated in Figure S5). Polymer sizes are also randomly sampled from a normal distribution (Table S2). The variations in polymer structures and sizes introduce diversity into the population while preventing the algorithm from getting stuck near local optima. The workflow in the Figure S4 is following:

1. Start, set distance $d = Inf$
2. Set monomer distribution $\boldsymbol{M}$, linkage distribution $\boldsymbol{L}$, and branching coefficients $\boldsymbol{X}$ (optional)
3. Set hyperparameters or use the default values provided in the example notebook
4. While the number of iterations ($i_{step}$) < the maximum number of iterations for optimizing a population ($i_{stop\_outer}$):
   1. Optimize a single polymer $P_{i}$
   2. Check Metropolis criterion on $d$, if met, add $P_{i}$ to the population
   3. $i_{step}= i_{step}+1$
5. For each polymer $P_{i}$:
   1. While the number of iterations ($i_{step\_intrapolynmer}$) < the maximum number of iterations ($i_{stop\_intrapolymer}$):
      1. Add a possible ring
      2. Check Metropolis criterion on $d$, if met, add the ring to the polymer $P_{i}$
      3. $i_{step\_intrapolynmer}= i_{step\_intrapolynmer}+1$
6. Characterize the population
7. Return the experimental observables and visualize the distributions
8. Save the SMILES strings of the structure population into CSV files

**Figure S4**. Hierarchical optimization scheme in LigninGraphs.

**Table S1**. Proposed structure additions, stopping criteria, and return values in each optimization loop.

| Optimization loop | Proposed structure additions | Results of acceptance | Stopping criteria | Return values |
| --- | --- | --- | --- | --- |
| Outer optimization for a population | Add a new polymer molecule $P_{i}$ from the inner loop | Population size (N) increases by 1 | The number of iterations ($i_{step}$) reaches the limit ($i_{stop\_outer}$) or the population size reaches the limit ($N_{stop}$) | The population; global metrics $\boldsymbol{M}_{\boldsymbol{sim}}\boldsymbol{,}\boldsymbol{L}_{\boldsymbol{sim}}\boldsymbol{,}\boldsymbol{X}_{\boldsymbol{sim}}$ |
| Inner optimization for a polymer molecule $P_{i}$ | Add a new monomer with a specific linkage | Polymer size ($n_{i})$increases by 1 | The number of iterations ($i_{step}$) reaches the limit ($i_{stop\_inner}$) or the polymer size reaches the limit (${n_{i}}_{stop}$ ) | The $P_{i}$ graph, its size $n_{i}$, and distance $d_{i}$ |
| Intramolecular linkage for a polymer molecule $P_{i}$ | Add a specific ring (intrapolymer linkage) within a polymer molecule $P_{i}$ | A ring is formed. Polymer $P_{i}$ is updated | The number of iterations ($i_{step}$) reaches the limit ($i_{stop\_intrapolymer}$) | The $P_{i}$ graph and distance $d_{i}$ |

**Figure S5**. Illustration of new monomer addition via random sampling. For instance, the experimental monomer distributions are 0, 0.37, and 0.63 for H, G, S; the linkage distributions are 0, 0, 0.78, 0, 0.07, and 0.15 for 4-O-5, 5-5, α-O-4, β-O-4, β -5, β-β; the branching propensity is 0.25. After random sampling for each property, with random numbers $r∊[0,1]$ shown in dials, a new G monomer is added via a β-O-4 to a terminal monomer to avoid branching.

**Hyperparameters**

Borrowing a similar concept from machine learning, we introduce hyperparameters to facilitate structure optimization. Unlike the monomer or linkage distributions, hyperparameters do not affect the outcome but control the runtime and accelerate convergence to the target values based on chemical intuition and knowledge. For instance, we could tune the size distribution to match the weighted molecular weight and control the branching propensity when adding a new monomer to reach the global branching coefficient. Table S2 shows a complete list of hyperparameters and their usages.

**Table S2**. List of hyperparameters in the multiscale optimization framework.

| Hyperparameter | Usage |
| --- | --- |
| Metropolis temperature ($T$) | Control the acceptance rate. The higher the temperature, the more likely a move with a positive $\Delta d$will be accepted. |
| Max number of iterations in each loop ($i_{stop\_inner}, i_{stop\_outer}, i_{stop\_intrapolymer}$) | Limit the number of MC attempts and runtime of the simulations. |
| Expected polymer size ($\bar{n}$) | Set a threshold for the polymer molecule $P_{i}$size (${n_{i}}_{stop}$) in the inner loop.  We consider the polymer size from a population following a normal distribution $\boldsymbol{N}$ with a mean of $\bar{n}$. The maximum $n_{max}$ and the minimum polymer size $(n_{min}=2$) should cover the 95% confidence interval for the size distribution. Therefore, the standard deviation $\sigma_{n}$ can be obtained from  $\sigma_{n}=\frac{n_{max}-n_{min}}{2z}$  where $z=1.96$ for 95% confidence interval. For each polymer $P_{i}$, we sample a random size (${n_{i}}_{stop}$) from the distribution to stop the polymer growth  ${n_{i}}_{stop}\sim\boldsymbol{N}(\bar{n}, \left( k\sigma_{n} \right)^{2})$  The scaling factor ($k$) controls the width of this distribution and affects the weighted average molecular weight. The larger the $k$, the wider the range of polymer sizes is; the weighted average molecular weight would be greater than the number average molecular weight, indicating the polymer population is polydisperse.  Note that if the experimental MW ($\bar{MW}$) is available, the polymer size can be expressed in MW. In this case, ${MW}_{min}=0$, ${MW}_{max}=10000$, and  $\sigma_{MW}=\frac{{MW}_{max}-{MW}_{min}}{2z}$  ${{MW}_{i}}_{stop}\sim\boldsymbol{N}(\bar{MW}, \left( k\sigma_{MW} \right)^{2})$ |
| Maximum polymer size ($n_{max}$) |  |
| Size distribution scaling factor ($k$) |  |
| Branching propensity | Determine the branching probability for newly added monomers. The value should be similar to the experimental branching coefficient.  A random number $r\in[0,1]$ is generated each time when adding a monomer. When r $\leq$branching propensity, a C1 in the branched (non-terminal) monomers is selected for bonding. A branched (non-terminal) monomer is connected to two or more monomers.  When r > branching propensity, a C1 in the terminal monomers is selected for bonding. A terminal monomer is only connected to one monomer. |

**Metropolis acceptance criteria**

Here, we utilize the general Metropolis Monte Carlo algorithm^2–5^ to minimize the distance between the structure metrics from the simulation and the target experimental values. We define $\boldsymbol{\sigma=[M, L, X]}$ as a set of metrics for a simulated molecule or population, where $\boldsymbol{M}$, $\boldsymbol{L}$, $\boldsymbol{X}$ are the monomer percentages, linkage percentages, and additional metrics, such as the branching coefficient, respectively. Using Eq. (1) in the main text, we obtain the distance of $\boldsymbol{\sigma}$ between this molecule or the population with target values $d$. When a move is proposed, the new set of metrics $\boldsymbol{\sigma}_{\boldsymbol{new}}$ is obtained. To accept or reject this move, we do the following:

1. Compute $\Delta d = d(\boldsymbol{\sigma}_{\boldsymbol{new}}) - d(\boldsymbol{\sigma})$.
2. If $\Delta d \leq0$, accept the move.
3. If $\Delta d > 0$, compute $w =e^{-\frac{\Delta d}{k_{B}T}}$. Generate a random number $r\in[0,1]$. If $r \leq w$, accept the move; otherwise, reject the move.
4. When the move is accepted, update the structure, $\boldsymbol{\sigma}=\boldsymbol{\sigma}_{\boldsymbol{new}}\boldsymbol{,}$ and the current distance $d\left( \boldsymbol{\sigma} \right)=d(\boldsymbol{\sigma}_{\boldsymbol{new}})$*.*
5. Return $\boldsymbol{\sigma}$ and $d(\boldsymbol{\sigma})$.

**Additional Note 3 –** **Structure metrics of simulated lignin libraries**

**Figure S6**. Poplar lignin structure metrics for the simulated optima and target values. Monomer percentages: a, H. b, G. c, S. Linkage distributions: d, 4-O-5. e, α-O-4. f, β-O-4. g, 5-5. h, β-5. i, β-β. The population's means (µ) and standard deviations (σ) are shown at the top of each subfigure.

**Figure S7**. Miscanthus lignin structure metrics for the simulated optima and target values. Monomer percentages: a, H. b, G. c, S; Linkage distributions: d, 4-O-5. e, α-O-4. f, β-O-4. g, 5-5. h, β-5. i, β-β. The population's means (µ) and standard deviations (σ) are shown at the top of each subfigure.

**Figure S8**. Correlations between (a) branching propensity, (b) 5-5 linkage percentage, (c) β-O-4 linkage percentage, and (d) β- β simulated branching coefficient for pine and poplar simulations. The values of Pearson correlation coefficients ($r_{pearson}$) are indicated. Values close to 1, -1, and 0 suggest a strong positive, negative, and weak correlation, respectively.

**Table S3**. Example structure library for pine lignin. The counts of monomers, linkages and functional groups, branching coefficient, and the smiles strings of the structures are shown.

|  | **H** | **G** | **S** | **4-O-5** | **alpha-O-4** | **beta-O-4** | **5-5** | **beta-5** | **beta-beta** | **beta-1** | **branching_coeff** | **MW** | **monomer_count** | **OH_count** | **OCH3_count** | **smiles** |
| --- | --- | --- | --- | --- | --- | --- | --- | --- | --- | --- | --- | --- | --- | --- | --- | --- |
| **0** | 0 | 5 | 0 | 0 | 0 | 2 | 0 | 1 | 1 | 0 | 0 | 897 | 5 | 5 | 5 | OCC1c2c(OC1c1cc(OC)c(OC(CO)Cc3cc(OC)c(OC(CO)Cc4cc(OC)c(O)cc4)cc3)cc1)c(OC)cc(C1C3COC(C3CO1)c1cc(OC)c(O)cc1)c2 |
| **1** | 0 | 6 | 0 | 0 | 0 | 4 | 0 | 1 | 0 | 0 | 0 | 1079 | 6 | 7 | 6 | OCC1C(Oc2c(OC)cc(CC(Oc3c(OC)cc(C=CCO)cc3)CO)cc21)c1cc(OC)c(OC(CO)Cc2cc(OC)c(OC(CO)Cc3cc(OC)c(OC(CO)Cc4cc(OC)c(O)cc4)cc3)cc2)cc1 |
| **2** | 0 | 5 | 0 | 0 | 0 | 3 | 0 | 1 | 0 | 0 | 0 | 899 | 5 | 6 | 5 | OCC(Oc1c(OC)cc(C=CCO)cc1)Cc1cc(OC)c(OC(CO)Cc2cc(OC)c3c(C(CO)C(c4cc(OC)c(OC(CO)Cc5cc(OC)c(O)cc5)cc4)O3)c2)cc1 |
| **3** | 0 | 5 | 0 | 0 | 0 | 3 | 0 | 0 | 1 | 0 | 0 | 899 | 5 | 5 | 5 | Oc1c(OC)cc(CC(CO)Oc2c(OC)cc(C3OCC4C(OCC43)c3cc(OC)c(OC(CO)Cc4cc(OC)c(OC(CO)Cc5cc(OC)c(O)cc5)cc4)cc3)cc2)cc1 |
| **4** | 0 | 14 | 0 | 0 | 0 | 9 | 0 | 3 | 1 | 0 | 0 | 2515 | 14 | 14 | 14 | OCC(Oc1c(OC)cc(C2C3COC(C3CO2)c2cc(OC)c(OC(CO)Cc3cc(OC)c(O)cc3)cc2)cc1)Cc1cc(OC)c2c(C(CO)C(c3cc(OC)c4c(C(CO)C(c5cc(OC)c(OC(CO)Cc6cc(OC)c(OC(CO)Cc7cc(OC)c(OC(CO)Cc8cc(OC)c(OC(CO)Cc9cc(OC)c(OC(CO)Cc%10cc(OC)c%11c(C(CO)C(c%12cc(OC)c(OC(CO)Cc%13cc(OC)c(OC(CO)Cc%14cc(OC)c(O)cc%14)cc%13)cc%12)O%11)c%10)cc9)cc8)cc7)cc6)cc5)O4)c3)O2)c1 |
| **5** | 0 | 6 | 0 | 0 | 0 | 4 | 0 | 0 | 1 | 0 | 0 | 1079 | 6 | 6 | 6 | OCC(Oc1c(OC)cc(CC(Oc2c(OC)cc(C3C4COC(C4CO3)c3cc(OC)c(O)cc3)cc2)CO)cc1)Cc1cc(OC)c(OC(CO)Cc2cc(OC)c(OC(CO)Cc3cc(OC)c(O)cc3)cc2)cc1 |
| **6** | 0 | 5 | 0 | 0 | 0 | 2 | 0 | 2 | 0 | 0 | 0 | 897 | 5 | 6 | 5 | OCC(Oc1c(OC)cc(C2C(c3c(O2)c(OC)cc(CC(Oc2c(OC)cc(C=CCO)cc2)CO)c3)CO)cc1)Cc1cc(OC)c2c(C(CO)C(c3cc(OC)c(O)cc3)O2)c1 |
| **7** | 0 | 6 | 0 | 0 | 0 | 4 | 0 | 0 | 1 | 0 | 0 | 1079 | 6 | 6 | 6 | OCC(Oc1c(OC)cc(CC(Oc2c(OC)cc(C3C4COC(C4CO3)c3cc(OC)c(O)cc3)cc2)CO)cc1)Cc1cc(OC)c(OC(CO)Cc2cc(OC)c(OC(CO)Cc3cc(OC)c(O)cc3)cc2)cc1 |
| **8** | 0 | 4 | 0 | 0 | 0 | 1 | 0 | 1 | 1 | 0 | 0 | 717 | 4 | 4 | 4 | OCC1c2c(OC1c1cc(OC)c(OC(CO)Cc3cc(OC)c(O)cc3)cc1)c(OC)cc(C1C3COC(C3CO1)c1cc(OC)c(O)cc1)c2 |
| **9** | 0 | 5 | 0 | 0 | 0 | 2 | 0 | 1 | 1 | 0 | 0 | 897 | 5 | 5 | 5 | Oc1c(OC)cc(CC(Oc2c(OC)cc(C3C4COC(C4CO3)c3cc(OC)c(OC(CO)Cc4cc(OC)c5c(C(CO)C(c6cc(OC)c(O)cc6)O5)c4)cc3)cc2)CO)cc1 |
| **10** | 0 | 4 | 0 | 0 | 0 | 2 | 0 | 1 | 0 | 0 | 0 | 719 | 4 | 5 | 4 | OCC(Oc1c(OC)cc(C=CCO)cc1)Cc1cc(OC)c2c(C(CO)C(c3cc(OC)c(OC(CO)Cc4cc(OC)c(O)cc4)cc3)O2)c1 |
| **11** | 0 | 5 | 0 | 0 | 0 | 2 | 0 | 2 | 0 | 0 | 0 | 897 | 5 | 6 | 5 | OCC1C(Oc2c(OC)cc(C3C(c4c(O3)c(OC)cc(C=CCO)c4)CO)cc21)c1cc(OC)c(OC(CO)Cc2cc(OC)c(OC(CO)Cc3cc(OC)c(O)cc3)cc2)cc1 |
| **12** | 0 | 12 | 0 | 0 | 0 | 8 | 0 | 2 | 1 | 0 | 0 | 2156 | 12 | 12 | 12 | OCC1c2c(OC1c1cc(OC)c3c(C(CO)C(c4cc(OC)c(OC(CO)Cc5cc(OC)c(OC(CO)Cc6cc(OC)c(OC(CO)Cc7cc(OC)c(OC(CO)Cc8cc(OC)c(O)cc8)cc7)cc6)cc5)cc4)O3)c1)c(OC)cc(C1C3COC(C3CO1)c1cc(OC)c(OC(CO)Cc3cc(OC)c(OC(CO)Cc4cc(OC)c(OC(CO)Cc5cc(OC)c(OC(CO)Cc6cc(OC)c(O)cc6)cc5)cc4)cc3)cc1)c2 |
| **13** | 0 | 6 | 0 | 0 | 0 | 4 | 0 | 1 | 0 | 0 | 0 | 1079 | 6 | 7 | 6 | OCC(Oc1c(OC)cc(CC(Oc2c(OC)cc(C3C(c4c(O3)c(OC)cc(CC(Oc3c(OC)cc(C=CCO)cc3)CO)c4)CO)cc2)CO)cc1)Cc1cc(OC)c(OC(CO)Cc2cc(OC)c(O)cc2)cc1 |
| **14** | 0 | 4 | 0 | 0 | 0 | 1 | 0 | 1 | 1 | 0 | 0 | 717 | 4 | 4 | 4 | OCC(Oc1c(OC)cc(C2C3COC(C3CO2)c2cc(OC)c(O)cc2)cc1)Cc1cc(OC)c2c(C(CO)C(c3cc(OC)c(O)cc3)O2)c1 |
| **15** | 0 | 4 | 0 | 0 | 0 | 1 | 0 | 1 | 1 | 0 | 0 | 717 | 4 | 4 | 4 | Oc1c(OC)cc(C2C3COC(C3CO2)c2cc(OC)c(OC(CO)Cc3cc(OC)c4c(C(CO)C(c5cc(OC)c(O)cc5)O4)c3)cc2)cc1 |
| **16** | 0 | 4 | 0 | 0 | 0 | 1 | 0 | 2 | 0 | 0 | 0 | 717 | 4 | 5 | 4 | OCC=Cc1cc(OC)c2c(C(CO)C(c3cc(OC)c(OC(CO)Cc4cc(OC)c5c(C(CO)C(c6cc(OC)c(O)cc6)O5)c4)cc3)O2)c1 |
| **17** | 0 | 7 | 0 | 0 | 0 | 6 | 0 | 0 | 0 | 0 | 0 | 1261 | 7 | 8 | 7 | OCC(Oc1c(OC)cc(CC(Oc2c(OC)cc(CC(Oc3c(OC)cc(CC(Oc4c(OC)cc(C=CCO)cc4)CO)cc3)CO)cc2)CO)cc1)Cc1cc(OC)c(OC(CO)Cc2cc(OC)c(OC(CO)Cc3cc(OC)c(O)cc3)cc2)cc1 |
| **18** | 0 | 5 | 0 | 0 | 0 | 3 | 0 | 1 | 0 | 0 | 0 | 899 | 5 | 6 | 5 | OCC(Oc1c(OC)cc(CC(Oc2c(OC)cc(C=CCO)cc2)CO)cc1)Cc1cc(OC)c2c(C(CO)C(c3cc(OC)c(OC(CO)Cc4cc(OC)c(O)cc4)cc3)O2)c1 |
| **19** | 0 | 5 | 0 | 0 | 0 | 1 | 0 | 2 | 1 | 0 | 0 | 895 | 5 | 5 | 5 | OCC1c2c(OC1c1cc(OC)c3c(C(CO)C(c4cc(OC)c(O)cc4)O3)c1)c(OC)cc(C1C3COC(C3CO1)c1cc(OC)c(OC(CO)Cc3cc(OC)c(O)cc3)cc1)c2 |
| **20** | 0 | 5 | 0 | 0 | 0 | 3 | 0 | 0 | 1 | 0 | 0 | 899 | 5 | 5 | 5 | OCC(Oc1c(OC)cc(CC(Oc2c(OC)cc(C3C4COC(C4CO3)c3cc(OC)c(O)cc3)cc2)CO)cc1)Cc1cc(OC)c(OC(CO)Cc2cc(OC)c(O)cc2)cc1 |
| **21** | 0 | 4 | 0 | 0 | 0 | 1 | 0 | 2 | 0 | 0 | 0 | 717 | 4 | 5 | 4 | Oc1c(OC)cc(CC(Oc2c(OC)cc(C3C(c4c(O3)c(OC)cc(C3C(c5c(O3)c(OC)cc(C=CCO)c5)CO)c4)CO)cc2)CO)cc1 |
| **22** | 0 | 4 | 0 | 0 | 0 | 3 | 0 | 0 | 0 | 0 | 0 | 721 | 4 | 5 | 4 | OCC(Oc1c(OC)cc(C=CCO)cc1)Cc1cc(OC)c(OC(CO)Cc2cc(OC)c(OC(CO)Cc3cc(OC)c(O)cc3)cc2)cc1 |
| **23** | 0 | 5 | 0 | 0 | 0 | 3 | 0 | 0 | 1 | 0 | 0 | 899 | 5 | 5 | 5 | OCC(Cc1cc(OC)c(OC(CO)Cc2cc(OC)c(O)cc2)cc1)Oc1c(OC)cc(C2OCC3C(OCC32)c2cc(OC)c(OC(CO)Cc3cc(OC)c(O)cc3)cc2)cc1 |
| **24** | 0 | 4 | 0 | 0 | 0 | 1 | 0 | 1 | 1 | 0 | 0 | 717 | 4 | 4 | 4 | Oc1c(OC)cc(CC(CO)Oc2c(OC)cc(C3C4COC(C4CO3)c3cc(OC)c4c(C(CO)C(c5cc(OC)c(O)cc5)O4)c3)cc2)cc1 |
| **25** | 0 | 6 | 0 | 0 | 0 | 4 | 0 | 0 | 1 | 0 | 0 | 1079 | 6 | 6 | 6 | OCC(Oc1c(OC)cc(CC(Oc2c(OC)cc(C3C4COC(C4CO3)c3cc(OC)c(O)cc3)cc2)CO)cc1)Cc1cc(OC)c(OC(CO)Cc2cc(OC)c(OC(CO)Cc3cc(OC)c(O)cc3)cc2)cc1 |
| **26** | 0 | 4 | 0 | 0 | 0 | 1 | 0 | 1 | 1 | 0 | 0 | 717 | 4 | 4 | 4 | OCC1c2c(OC1c1cc(OC)c(OC(CO)Cc3cc(OC)c(O)cc3)cc1)c(OC)cc(C1C3COC(C3CO1)c1cc(OC)c(O)cc1)c2 |
| **27** | 0 | 7 | 0 | 0 | 0 | 5 | 0 | 1 | 0 | 0 | 0 | 1259 | 7 | 8 | 7 | OCC(Oc1c(OC)cc(CC(Oc2c(OC)cc(C=CCO)cc2)CO)cc1)Cc1cc(OC)c(OC(CO)Cc2cc(OC)c(OC(CO)Cc3cc(OC)c(OC(CO)Cc4cc(OC)c5c(C(CO)C(c6cc(OC)c(O)cc6)O5)c4)cc3)cc2)cc1 |
| **28** | 0 | 5 | 0 | 0 | 0 | 4 | 0 | 0 | 0 | 0 | 0 | 901 | 5 | 6 | 5 | OCC(Oc1c(OC)cc(CC(Oc2c(OC)cc(C=CCO)cc2)CO)cc1)Cc1cc(OC)c(OC(CO)Cc2cc(OC)c(OC(CO)Cc3cc(OC)c(O)cc3)cc2)cc1 |
| **29** | 0 | 7 | 0 | 0 | 0 | 4 | 0 | 2 | 0 | 0 | 0 | 1257 | 7 | 8 | 7 | OCC(Oc1c(OC)cc(C2C(c3c(O2)c(OC)cc(C2C(c4c(O2)c(OC)cc(CC(Oc2c(OC)cc(C=CCO)cc2)CO)c4)CO)c3)CO)cc1)Cc1cc(OC)c(OC(CO)Cc2cc(OC)c(OC(CO)Cc3cc(OC)c(O)cc3)cc2)cc1 |
| **30** | 0 | 7 | 0 | 0 | 0 | 4 | 0 | 1 | 1 | 0 | 0 | 1257 | 7 | 7 | 7 | OCC1c2c(OC1c1cc(OC)c(OC(CO)Cc3cc(OC)c(OC(CO)Cc4cc(OC)c(OC(CO)Cc5cc(OC)c(O)cc5)cc4)cc3)cc1)c(OC)cc(C1C3COC(C3CO1)c1cc(OC)c(OC(CO)Cc3cc(OC)c(O)cc3)cc1)c2 |
| **31** | 0 | 5 | 0 | 0 | 0 | 3 | 0 | 1 | 0 | 0 | 0 | 899 | 5 | 6 | 5 | OCC(Oc1c(OC)cc(CC(Oc2c(OC)cc(C=CCO)cc2)CO)cc1)Cc1cc(OC)c2c(C(CO)C(c3cc(OC)c(OC(CO)Cc4cc(OC)c(O)cc4)cc3)O2)c1 |
| **32** | 0 | 5 | 0 | 0 | 0 | 3 | 0 | 1 | 0 | 0 | 0 | 899 | 5 | 6 | 5 | OCC(Oc1c(OC)cc(C=CCO)cc1)Cc1cc(OC)c(OC(CO)Cc2cc(OC)c3c(C(CO)C(c4cc(OC)c(OC(CO)Cc5cc(OC)c(O)cc5)cc4)O3)c2)cc1 |
| **33** | 0 | 5 | 0 | 0 | 0 | 2 | 0 | 1 | 1 | 0 | 0 | 897 | 5 | 5 | 5 | OCC(Cc1cc(OC)c(OC(CO)Cc2cc(OC)c(O)cc2)cc1)Oc1c(OC)cc(C2OCC3C(OCC32)c2cc(OC)c3c(C(CO)C(c4cc(OC)c(O)cc4)O3)c2)cc1 |
| **34** | 0 | 9 | 0 | 0 | 0 | 6 | 0 | 2 | 0 | 0 | 0 | 1618 | 9 | 10 | 9 | OCC(Oc1c(OC)cc(C=CCO)cc1)Cc1cc(OC)c(OC(CO)Cc2cc(OC)c(OC(CO)Cc3cc(OC)c4c(C(CO)C(c5cc(OC)c6c(C(CO)C(c7cc(OC)c(OC(CO)Cc8cc(OC)c(OC(CO)Cc9cc(OC)c(OC(CO)Cc%10cc(OC)c(O)cc%10)cc9)cc8)cc7)O6)c5)O4)c3)cc2)cc1 |
| **35** | 0 | 7 | 0 | 0 | 0 | 5 | 0 | 1 | 0 | 0 | 0 | 1259 | 7 | 8 | 7 | OCC(Oc1c(OC)cc(CC(Oc2c(OC)cc(C3C(c4c(O3)c(OC)cc(C=CCO)c4)CO)cc2)CO)cc1)Cc1cc(OC)c(OC(CO)Cc2cc(OC)c(OC(CO)Cc3cc(OC)c(OC(CO)Cc4cc(OC)c(O)cc4)cc3)cc2)cc1 |
| **36** | 0 | 4 | 0 | 0 | 0 | 1 | 0 | 1 | 1 | 0 | 0 | 717 | 4 | 4 | 4 | Oc1c(OC)cc(CC(Oc2c(OC)cc(C3C(c4c(O3)c(OC)cc(C3C5COC(C5CO3)c3cc(OC)c(O)cc3)c4)CO)cc2)CO)cc1 |
| **37** | 0 | 4 | 0 | 0 | 0 | 1 | 0 | 1 | 1 | 0 | 0 | 717 | 4 | 4 | 4 | Oc1c(OC)cc(C2C3COC(C3CO2)c2cc(OC)c(OC(CO)Cc3cc(OC)c4c(C(CO)C(c5cc(OC)c(O)cc5)O4)c3)cc2)cc1 |
| **38** | 0 | 4 | 0 | 0 | 0 | 2 | 0 | 0 | 1 | 0 | 0 | 719 | 4 | 4 | 4 | Oc1c(OC)cc(C2C3COC(C3CO2)c2cc(OC)c(OC(CO)Cc3cc(OC)c(OC(CO)Cc4cc(OC)c(O)cc4)cc3)cc2)cc1 |
| **39** | 0 | 5 | 0 | 0 | 0 | 2 | 0 | 1 | 1 | 0 | 0 | 897 | 5 | 5 | 5 | OCC(Oc1c(OC)cc(C2C3COC(C3CO2)c2cc(OC)c3c(C(CO)C(c4cc(OC)c(O)cc4)O3)c2)cc1)Cc1cc(OC)c(OC(CO)Cc2cc(OC)c(O)cc2)cc1 |
| **40** | 0 | 5 | 0 | 0 | 0 | 2 | 0 | 1 | 1 | 0 | 0 | 897 | 5 | 5 | 5 | OCC(Oc1c(OC)cc(C2C3COC(C3CO2)c2cc(OC)c(O)cc2)cc1)Cc1cc(OC)c(OC(CO)Cc2cc(OC)c3c(C(CO)C(c4cc(OC)c(O)cc4)O3)c2)cc1 |
| **41** | 0 | 5 | 0 | 0 | 0 | 3 | 0 | 0 | 1 | 0 | 0 | 899 | 5 | 5 | 5 | OCC(Oc1c(OC)cc(C2C3COC(C3CO2)c2cc(OC)c(O)cc2)cc1)Cc1cc(OC)c(OC(CO)Cc2cc(OC)c(OC(CO)Cc3cc(OC)c(O)cc3)cc2)cc1 |
| **42** | 0 | 6 | 0 | 0 | 0 | 4 | 0 | 0 | 1 | 0 | 0 | 1079 | 6 | 6 | 6 | OCC(Oc1c(OC)cc(CC(Oc2c(OC)cc(C3C4COC(C4CO3)c3cc(OC)c(OC(CO)Cc4cc(OC)c(O)cc4)cc3)cc2)CO)cc1)Cc1cc(OC)c(OC(CO)Cc2cc(OC)c(O)cc2)cc1 |
| **43** | 0 | 4 | 0 | 0 | 0 | 3 | 0 | 0 | 0 | 0 | 0 | 721 | 4 | 5 | 4 | Oc1c(OC)cc(CC(Oc2c(OC)cc(CC(Oc3c(OC)cc(CC(Oc4c(OC)cc(C=CCO)cc4)CO)cc3)CO)cc2)CO)cc1 |
| **44** | 0 | 5 | 0 | 0 | 0 | 2 | 0 | 1 | 1 | 0 | 0 | 897 | 5 | 5 | 5 | OCC(Oc1c(OC)cc(C2C3COC(C3CO2)c2cc(OC)c(O)cc2)cc1)Cc1cc(OC)c(OC(CO)Cc2cc(OC)c3c(C(CO)C(c4cc(OC)c(O)cc4)O3)c2)cc1 |
| **45** | 0 | 5 | 0 | 0 | 0 | 3 | 0 | 0 | 1 | 0 | 0 | 899 | 5 | 5 | 5 | Oc1c(OC)cc(C2C3COC(C3CO2)c2cc(OC)c(OC(CO)Cc3cc(OC)c(OC(CO)Cc4cc(OC)c(OC(CO)Cc5cc(OC)c(O)cc5)cc4)cc3)cc2)cc1 |
| **46** | 0 | 5 | 0 | 0 | 0 | 3 | 0 | 1 | 0 | 0 | 0 | 899 | 5 | 6 | 5 | OCC1C(Oc2c(OC)cc(C=CCO)cc12)c1cc(OC)c(OC(CO)Cc2cc(OC)c(OC(CO)Cc3cc(OC)c(OC(CO)Cc4cc(OC)c(O)cc4)cc3)cc2)cc1 |
| **47** | 0 | 6 | 0 | 0 | 0 | 4 | 0 | 0 | 1 | 0 | 0 | 1079 | 6 | 6 | 6 | OCC(Oc1c(OC)cc(C2C3COC(C3CO2)c2cc(OC)c(OC(CO)Cc3cc(OC)c(O)cc3)cc2)cc1)Cc1cc(OC)c(OC(CO)Cc2cc(OC)c(OC(CO)Cc3cc(OC)c(O)cc3)cc2)cc1 |
| **48** | 0 | 6 | 0 | 0 | 0 | 4 | 0 | 0 | 1 | 0 | 0 | 1079 | 6 | 6 | 6 | OCC(Oc1c(OC)cc(CC(Oc2c(OC)cc(CC(Oc3c(OC)cc(C4C5COC(C5CO4)c4cc(OC)c(O)cc4)cc3)CO)cc2)CO)cc1)Cc1cc(OC)c(OC(CO)Cc2cc(OC)c(O)cc2)cc1 |
| **49** | 0 | 8 | 0 | 0 | 0 | 7 | 0 | 0 | 0 | 0 | 0 | 1442 | 8 | 9 | 8 | OCC(Oc1c(OC)cc(CC(Oc2c(OC)cc(C=CCO)cc2)CO)cc1)Cc1cc(OC)c(OC(CO)Cc2cc(OC)c(OC(CO)Cc3cc(OC)c(OC(CO)Cc4cc(OC)c(OC(CO)Cc5cc(OC)c(OC(CO)Cc6cc(OC)c(O)cc6)cc5)cc4)cc3)cc2)cc1 |
| **50** | 0 | 7 | 0 | 0 | 0 | 5 | 0 | 0 | 1 | 0 | 0 | 1259 | 7 | 7 | 7 | OCC(Oc1c(OC)cc(C2C3COC(C3CO2)c2cc(OC)c(O)cc2)cc1)Cc1cc(OC)c(OC(CO)Cc2cc(OC)c(OC(CO)Cc3cc(OC)c(OC(CO)Cc4cc(OC)c(OC(CO)Cc5cc(OC)c(O)cc5)cc4)cc3)cc2)cc1 |
| **51** | 0 | 7 | 0 | 0 | 0 | 6 | 0 | 0 | 0 | 0 | 0 | 1261 | 7 | 8 | 7 | OCC(Oc1c(OC)cc(CC(Oc2c(OC)cc(CC(Oc3c(OC)cc(C=CCO)cc3)CO)cc2)CO)cc1)Cc1cc(OC)c(OC(CO)Cc2cc(OC)c(OC(CO)Cc3cc(OC)c(OC(CO)Cc4cc(OC)c(O)cc4)cc3)cc2)cc1 |
| **52** | 0 | 7 | 0 | 0 | 0 | 5 | 0 | 0 | 1 | 0 | 0 | 1259 | 7 | 7 | 7 | OCC(Oc1c(OC)cc(CC(Oc2c(OC)cc(C3C4COC(C4CO3)c3cc(OC)c(OC(CO)Cc4cc(OC)c(O)cc4)cc3)cc2)CO)cc1)Cc1cc(OC)c(OC(CO)Cc2cc(OC)c(OC(CO)Cc3cc(OC)c(O)cc3)cc2)cc1 |
| **53** | 0 | 5 | 0 | 0 | 0 | 4 | 0 | 0 | 0 | 0 | 0 | 901 | 5 | 6 | 5 | OCC(Oc1c(OC)cc(C=CCO)cc1)Cc1cc(OC)c(OC(CO)Cc2cc(OC)c(OC(CO)Cc3cc(OC)c(OC(CO)Cc4cc(OC)c(O)cc4)cc3)cc2)cc1 |
| **54** | 0 | 5 | 0 | 0 | 0 | 2 | 0 | 2 | 0 | 0 | 0 | 897 | 5 | 6 | 5 | OCC1C(Oc2c(OC)cc(C3C(c4c(O3)c(OC)cc(C=CCO)c4)CO)cc12)c1cc(OC)c(OC(CO)Cc2cc(OC)c(OC(CO)Cc3cc(OC)c(O)cc3)cc2)cc1 |
| **55** | 0 | 8 | 0 | 0 | 0 | 5 | 0 | 1 | 1 | 0 | 0 | 1438 | 8 | 8 | 8 | OCC(Oc1c(OC)cc(CC(Oc2c(OC)cc(C3C4COC(C4CO3)c3cc(OC)c4c(C(CO)C(c5cc(OC)c(OC(CO)Cc6cc(OC)c(O)cc6)cc5)O4)c3)cc2)CO)cc1)Cc1cc(OC)c(OC(CO)Cc2cc(OC)c(OC(CO)Cc3cc(OC)c(O)cc3)cc2)cc1 |
| **56** | 0 | 6 | 0 | 0 | 0 | 2 | 0 | 3 | 0 | 0 | 0 | 1075 | 6 | 7 | 6 | OCC1C(Oc2c(OC)cc(C3C(c4c(O3)c(OC)cc(C=CCO)c4)CO)cc21)c1cc(OC)c(OC(CO)Cc2cc(OC)c(OC(CO)Cc3cc(OC)c4c(C(CO)C(c5cc(OC)c(O)cc5)O4)c3)cc2)cc1 |
| **57** | 0 | 5 | 0 | 0 | 0 | 3 | 0 | 0 | 1 | 0 | 0 | 899 | 5 | 5 | 5 | OCC(Cc1cc(OC)c(OC(CO)Cc2cc(OC)c(O)cc2)cc1)Oc1c(OC)cc(C2OCC3C(OCC32)c2cc(OC)c(OC(CO)Cc3cc(OC)c(O)cc3)cc2)cc1 |
| **58** | 0 | 5 | 0 | 0 | 0 | 3 | 0 | 0 | 1 | 0 | 0 | 899 | 5 | 5 | 5 | OCC(Oc1c(OC)cc(C2C3COC(C3CO2)c2cc(OC)c(O)cc2)cc1)Cc1cc(OC)c(OC(CO)Cc2cc(OC)c(OC(CO)Cc3cc(OC)c(O)cc3)cc2)cc1 |
| **59** | 0 | 4 | 0 | 0 | 0 | 2 | 0 | 1 | 0 | 0 | 0 | 719 | 4 | 5 | 4 | OCC1C(Oc2c(OC)cc(CC(Oc3c(OC)cc(C=CCO)cc3)CO)cc21)c1cc(OC)c(OC(CO)Cc2cc(OC)c(O)cc2)cc1 |
| **60** | 0 | 4 | 0 | 0 | 0 | 1 | 0 | 2 | 0 | 0 | 0 | 717 | 4 | 5 | 4 | OCC1C(Oc2c(OC)cc(C=CCO)cc21)c1cc(OC)c(OC(CO)Cc2cc(OC)c3c(C(CO)C(c4cc(OC)c(O)cc4)O3)c2)cc1 |
| **61** | 0 | 5 | 0 | 0 | 0 | 3 | 0 | 0 | 1 | 0 | 0 | 899 | 5 | 5 | 5 | OCC(Cc1cc(OC)c(OC(CO)Cc2cc(OC)c(OC(CO)Cc3cc(OC)c(O)cc3)cc2)cc1)Oc1c(OC)cc(C2C3COC(C3CO2)c2cc(OC)c(O)cc2)cc1 |
| **62** | 0 | 4 | 0 | 0 | 0 | 1 | 0 | 1 | 1 | 0 | 0 | 717 | 4 | 4 | 4 | OCC1c2c(OC1c1cc(OC)c(OC(CO)Cc3cc(OC)c(O)cc3)cc1)c(OC)cc(C1C3COC(C3CO1)c1cc(OC)c(O)cc1)c2 |
| **63** | 0 | 6 | 0 | 0 | 0 | 5 | 0 | 0 | 0 | 0 | 0 | 1081 | 6 | 7 | 6 | OCC(Oc1c(OC)cc(CC(Oc2c(OC)cc(C=CCO)cc2)CO)cc1)Cc1cc(OC)c(OC(CO)Cc2cc(OC)c(OC(CO)Cc3cc(OC)c(OC(CO)Cc4cc(OC)c(O)cc4)cc3)cc2)cc1 |
| **64** | 0 | 5 | 0 | 0 | 0 | 2 | 0 | 2 | 0 | 0 | 0 | 897 | 5 | 6 | 5 | Oc1c(OC)cc(C2C(c3c(O2)c(OC)cc(C2C(c4c(O2)c(OC)cc(CC(Oc2c(OC)cc(CC(Oc5c(OC)cc(C=CCO)cc5)CO)cc2)CO)c4)CO)c3)CO)cc1 |
| **65** | 0 | 6 | 0 | 0 | 0 | 3 | 0 | 2 | 0 | 0 | 0 | 1077 | 6 | 7 | 6 | OCC1C(Oc2c(OC)cc(CC(Oc3c(OC)cc(C=CCO)cc3)CO)cc12)c1cc(OC)c2c(C(CO)C(c3cc(OC)c(OC(CO)Cc4cc(OC)c(OC(CO)Cc5cc(OC)c(O)cc5)cc4)cc3)O2)c1 |
| **66** | 0 | 5 | 0 | 0 | 0 | 2 | 0 | 1 | 1 | 0 | 0 | 897 | 5 | 5 | 5 | OCC(Cc1cc(OC)c(OC(CO)Cc2cc(OC)c(O)cc2)cc1)Oc1c(OC)cc(C2C3COC(C3CO2)c2cc(OC)c3c(C(CO)C(c4cc(OC)c(O)cc4)O3)c2)cc1 |
| **67** | 0 | 4 | 0 | 0 | 0 | 1 | 0 | 1 | 1 | 0 | 0 | 717 | 4 | 4 | 4 | OCC(Oc1c(OC)cc(C2C3COC(C3CO2)c2cc(OC)c(O)cc2)cc1)Cc1cc(OC)c2c(C(CO)C(c3cc(OC)c(O)cc3)O2)c1 |
| **68** | 0 | 7 | 0 | 0 | 0 | 2 | 0 | 4 | 0 | 0 | 0 | 1253 | 7 | 8 | 7 | OCC1C(Oc2c(OC)cc(C3C(c4c(O3)c(OC)cc(C3C(c5c(O3)c(OC)cc(C=CCO)c5)CO)c4)CO)cc21)c1cc(OC)c2c(C(CO)C(c3cc(OC)c(OC(CO)Cc4cc(OC)c(OC(CO)Cc5cc(OC)c(O)cc5)cc4)cc3)O2)c1 |
| **69** | 0 | 6 | 0 | 0 | 0 | 5 | 0 | 0 | 0 | 0 | 0 | 1081 | 6 | 7 | 6 | OCC(Oc1c(OC)cc(CC(Oc2c(OC)cc(CC(Oc3c(OC)cc(CC(Oc4c(OC)cc(C=CCO)cc4)CO)cc3)CO)cc2)CO)cc1)Cc1cc(OC)c(OC(CO)Cc2cc(OC)c(O)cc2)cc1 |
| **70** | 0 | 5 | 0 | 0 | 0 | 3 | 0 | 0 | 1 | 0 | 0 | 899 | 5 | 5 | 5 | OCC(Oc1c(OC)cc(CC(Oc2c(OC)cc(C3C4COC(C4CO3)c3cc(OC)c(O)cc3)cc2)CO)cc1)Cc1cc(OC)c(OC(CO)Cc2cc(OC)c(O)cc2)cc1 |
| **71** | 0 | 4 | 0 | 0 | 0 | 1 | 0 | 1 | 1 | 0 | 0 | 717 | 4 | 4 | 4 | Oc1c(OC)cc(CC(CO)Oc2c(OC)cc(C3OCC4C(OCC43)c3cc(OC)c4c(C(CO)C(c5cc(OC)c(O)cc5)O4)c3)cc2)cc1 |
| **72** | 0 | 5 | 0 | 0 | 0 | 3 | 0 | 0 | 1 | 0 | 0 | 899 | 5 | 5 | 5 | OCC(Cc1cc(OC)c(OC(CO)Cc2cc(OC)c(O)cc2)cc1)Oc1c(OC)cc(C2C3COC(C3CO2)c2cc(OC)c(OC(CO)Cc3cc(OC)c(O)cc3)cc2)cc1 |
| **73** | 0 | 6 | 0 | 0 | 0 | 5 | 0 | 0 | 0 | 0 | 0 | 1081 | 6 | 7 | 6 | OCC(Oc1c(OC)cc(CC(Oc2c(OC)cc(CC(Oc3c(OC)cc(C=CCO)cc3)CO)cc2)CO)cc1)Cc1cc(OC)c(OC(CO)Cc2cc(OC)c(OC(CO)Cc3cc(OC)c(O)cc3)cc2)cc1 |
| **74** | 0 | 5 | 0 | 0 | 0 | 3 | 0 | 0 | 1 | 0 | 0 | 899 | 5 | 5 | 5 | OCC(Cc1cc(OC)c(OC(CO)Cc2cc(OC)c(OC(CO)Cc3cc(OC)c(O)cc3)cc2)cc1)Oc1c(OC)cc(C2C3COC(C3CO2)c2cc(OC)c(O)cc2)cc1 |
| **75** | 0 | 6 | 0 | 0 | 0 | 5 | 0 | 0 | 0 | 0 | 0 | 1081 | 6 | 7 | 6 | OCC=Cc1cc(OC)c(OC(CO)Cc2cc(OC)c(OC(CO)Cc3cc(OC)c(OC(CO)Cc4cc(OC)c(OC(CO)Cc5cc(OC)c(OC(CO)Cc6cc(OC)c(O)cc6)cc5)cc4)cc3)cc2)cc1 |
| **76** | 0 | 4 | 0 | 0 | 0 | 1 | 0 | 1 | 1 | 0 | 0 | 717 | 4 | 4 | 4 | Oc1c(OC)cc(C2C(c3c(O2)c(OC)cc(CC(Oc2c(OC)cc(C4C5COC(C5CO4)c4cc(OC)c(O)cc4)cc2)CO)c3)CO)cc1 |
| **77** | 0 | 4 | 0 | 0 | 0 | 1 | 0 | 1 | 1 | 0 | 0 | 717 | 4 | 4 | 4 | Oc1c(OC)cc(CC(Oc2c(OC)cc(C3C4COC(C4CO3)c3cc(OC)c4c(C(CO)C(c5cc(OC)c(O)cc5)O4)c3)cc2)CO)cc1 |
| **78** | 0 | 6 | 0 | 0 | 0 | 5 | 0 | 0 | 0 | 0 | 0 | 1081 | 6 | 7 | 6 | OCC(Oc1c(OC)cc(CC(Oc2c(OC)cc(CC(Oc3c(OC)cc(C=CCO)cc3)CO)cc2)CO)cc1)Cc1cc(OC)c(OC(CO)Cc2cc(OC)c(OC(CO)Cc3cc(OC)c(O)cc3)cc2)cc1 |
| **79** | 0 | 4 | 0 | 0 | 0 | 1 | 0 | 1 | 1 | 0 | 0 | 717 | 4 | 4 | 4 | OCC(Cc1cc(OC)c2c(C(CO)C(c3cc(OC)c(O)cc3)O2)c1)Oc1c(OC)cc(C2C3COC(C3CO2)c2cc(OC)c(O)cc2)cc1 |
| **80** | 0 | 5 | 0 | 0 | 0 | 3 | 0 | 0 | 1 | 0 | 0 | 899 | 5 | 5 | 5 | Oc1c(OC)cc(CC(CO)Oc2c(OC)cc(C3OCC4C(OCC43)c3cc(OC)c(OC(CO)Cc4cc(OC)c(OC(CO)Cc5cc(OC)c(O)cc5)cc4)cc3)cc2)cc1 |
| **81** | 0 | 6 | 0 | 0 | 0 | 4 | 0 | 0 | 1 | 0 | 0 | 1079 | 6 | 6 | 6 | OCC(Cc1cc(OC)c(OC(CO)Cc2cc(OC)c(OC(CO)Cc3cc(OC)c(O)cc3)cc2)cc1)Oc1c(OC)cc(C2OCC3C(OCC32)c2cc(OC)c(OC(CO)Cc3cc(OC)c(O)cc3)cc2)cc1 |
| **82** | 0 | 5 | 0 | 0 | 0 | 3 | 0 | 1 | 0 | 0 | 0 | 899 | 5 | 6 | 5 | OCC1C(Oc2c(OC)cc(CC(Oc3c(OC)cc(C=CCO)cc3)CO)cc12)c1cc(OC)c(OC(CO)Cc2cc(OC)c(OC(CO)Cc3cc(OC)c(O)cc3)cc2)cc1 |
| **83** | 0 | 6 | 0 | 0 | 0 | 4 | 0 | 1 | 0 | 0 | 0 | 1079 | 6 | 7 | 6 | OCC(Oc1c(OC)cc(CC(Oc2c(OC)cc(CC(Oc3c(OC)cc(C4C(c5c(O4)c(OC)cc(C=CCO)c5)CO)cc3)CO)cc2)CO)cc1)Cc1cc(OC)c(OC(CO)Cc2cc(OC)c(O)cc2)cc1 |
| **84** | 0 | 4 | 0 | 0 | 0 | 1 | 0 | 1 | 1 | 0 | 0 | 717 | 4 | 4 | 4 | OCC(Cc1cc(OC)c2c(C(CO)C(c3cc(OC)c(O)cc3)O2)c1)Oc1c(OC)cc(C2C3COC(C3CO2)c2cc(OC)c(O)cc2)cc1 |
| **85** | 0 | 4 | 0 | 0 | 0 | 2 | 0 | 1 | 0 | 0 | 0 | 719 | 4 | 5 | 4 | OCC(Oc1c(OC)cc(C=CCO)cc1)Cc1cc(OC)c(OC(CO)Cc2cc(OC)c3c(C(CO)C(c4cc(OC)c(O)cc4)O3)c2)cc1 |
| **86** | 0 | 6 | 0 | 0 | 0 | 1 | 0 | 3 | 1 | 0 | 0 | 1073 | 6 | 6 | 6 | OCC1C(Oc2c(OC)cc(C3C(c4c(O3)c(OC)cc(C3C5COC(C5CO3)c3cc(OC)c(O)cc3)c4)CO)cc21)c1cc(OC)c2c(C(CO)C(c3cc(OC)c(OC(CO)Cc4cc(OC)c(O)cc4)cc3)O2)c1 |
| **87** | 0 | 4 | 0 | 0 | 0 | 2 | 0 | 1 | 0 | 0 | 0 | 719 | 4 | 5 | 4 | OCC(Oc1c(OC)cc(C=CCO)cc1)Cc1cc(OC)c(OC(CO)Cc2cc(OC)c3c(C(CO)C(c4cc(OC)c(O)cc4)O3)c2)cc1 |
| **88** | 0 | 5 | 0 | 0 | 0 | 3 | 0 | 0 | 1 | 0 | 0 | 899 | 5 | 5 | 5 | OCC(Oc1c(OC)cc(C2C3COC(C3CO2)c2cc(OC)c(OC(CO)Cc3cc(OC)c(O)cc3)cc2)cc1)Cc1cc(OC)c(OC(CO)Cc2cc(OC)c(O)cc2)cc1 |
| **89** | 0 | 7 | 0 | 0 | 0 | 5 | 0 | 0 | 1 | 0 | 0 | 1259 | 7 | 7 | 7 | Oc1c(OC)cc(CC(Oc2c(OC)cc(CC(Oc3c(OC)cc(CC(Oc4c(OC)cc(CC(Oc5c(OC)cc(C6C7COC(C7CO6)c6cc(OC)c(OC(CO)Cc7cc(OC)c(O)cc7)cc6)cc5)CO)cc4)CO)cc3)CO)cc2)CO)cc1 |
| **90** | 0 | 4 | 0 | 0 | 0 | 1 | 0 | 1 | 1 | 0 | 0 | 717 | 4 | 4 | 4 | Oc1c(OC)cc(CC(CO)Oc2c(OC)cc(C3C4COC(C4CO3)c3cc(OC)c4c(C(CO)C(c5cc(OC)c(O)cc5)O4)c3)cc2)cc1 |
| **91** | 0 | 5 | 0 | 0 | 0 | 3 | 0 | 1 | 0 | 0 | 0 | 899 | 5 | 6 | 5 | OCC(Oc1c(OC)cc(C=CCO)cc1)Cc1cc(OC)c2c(C(CO)C(c3cc(OC)c(OC(CO)Cc4cc(OC)c(OC(CO)Cc5cc(OC)c(O)cc5)cc4)cc3)O2)c1 |
| **92** | 0 | 4 | 0 | 0 | 0 | 2 | 0 | 1 | 0 | 0 | 0 | 719 | 4 | 5 | 4 | OCC1C(Oc2c(OC)cc(C=CCO)cc12)c1cc(OC)c(OC(CO)Cc2cc(OC)c(OC(CO)Cc3cc(OC)c(O)cc3)cc2)cc1 |
| **93** | 0 | 5 | 0 | 0 | 0 | 3 | 0 | 1 | 0 | 0 | 0 | 899 | 5 | 6 | 5 | OCC(Oc1c(OC)cc(C2C(c3c(O2)c(OC)cc(C=CCO)c3)CO)cc1)Cc1cc(OC)c(OC(CO)Cc2cc(OC)c(OC(CO)Cc3cc(OC)c(O)cc3)cc2)cc1 |
| **94** | 0 | 9 | 0 | 0 | 0 | 7 | 0 | 1 | 0 | 0 | 0 | 1620 | 9 | 10 | 9 | OCC(Oc1c(OC)cc(CC(Oc2c(OC)cc(CC(Oc3c(OC)cc(C4C(c5c(O4)c(OC)cc(C=CCO)c5)CO)cc3)CO)cc2)CO)cc1)Cc1cc(OC)c(OC(CO)Cc2cc(OC)c(OC(CO)Cc3cc(OC)c(OC(CO)Cc4cc(OC)c(OC(CO)Cc5cc(OC)c(O)cc5)cc4)cc3)cc2)cc1 |
| **95** | 0 | 5 | 0 | 0 | 0 | 3 | 0 | 1 | 0 | 0 | 0 | 899 | 5 | 6 | 5 | OCC(Oc1c(OC)cc(C=CCO)cc1)Cc1cc(OC)c2c(C(CO)C(c3cc(OC)c(OC(CO)Cc4cc(OC)c(OC(CO)Cc5cc(OC)c(O)cc5)cc4)cc3)O2)c1 |
| **96** | 0 | 4 | 0 | 0 | 0 | 2 | 0 | 1 | 0 | 0 | 0 | 719 | 4 | 5 | 4 | Oc1c(OC)cc(CC(Oc2c(OC)cc(CC(Oc3c(OC)cc(C4C(c5c(O4)c(OC)cc(C=CCO)c5)CO)cc3)CO)cc2)CO)cc1 |
| **97** | 0 | 7 | 0 | 0 | 0 | 3 | 0 | 2 | 1 | 0 | 0 | 1255 | 7 | 7 | 7 | OCC(Oc1c(OC)cc(C2C(c3c(O2)c(OC)cc(C2C4COC(C4CO2)c2cc(OC)c(O)cc2)c3)CO)cc1)Cc1cc(OC)c(OC(CO)Cc2cc(OC)c(OC(CO)Cc3cc(OC)c4c(C(CO)C(c5cc(OC)c(O)cc5)O4)c3)cc2)cc1 |
| **98** | 0 | 5 | 0 | 0 | 0 | 3 | 0 | 0 | 1 | 0 | 0 | 899 | 5 | 5 | 5 | Oc1c(OC)cc(CC(CO)Oc2c(OC)cc(C3C4COC(C4CO3)c3cc(OC)c(OC(CO)Cc4cc(OC)c(OC(CO)Cc5cc(OC)c(O)cc5)cc4)cc3)cc2)cc1 |
| **99** | 0 | 11 | 0 | 0 | 0 | 7 | 0 | 2 | 1 | 0 | 0 | 1976 | 11 | 11 | 11 | OCC(Oc1c(OC)cc(C2C(c3c(O2)c(OC)cc(CC(Oc2c(OC)cc(CC(Oc4c(OC)cc(C5C6COC(C6CO5)c5cc(OC)c(OC(CO)Cc6cc(OC)c(O)cc6)cc5)cc4)CO)cc2)CO)c3)CO)cc1)Cc1cc(OC)c2c(C(CO)C(c3cc(OC)c(OC(CO)Cc4cc(OC)c(OC(CO)Cc5cc(OC)c(OC(CO)Cc6cc(OC)c(O)cc6)cc5)cc4)cc3)O2)c1 |
| **100** | 0 | 5 | 0 | 0 | 0 | 3 | 0 | 1 | 0 | 0 | 0 | 899 | 5 | 6 | 5 | OCC(Oc1c(OC)cc(C2C(c3c(O2)c(OC)cc(CC(Oc2c(OC)cc(C=CCO)cc2)CO)c3)CO)cc1)Cc1cc(OC)c(OC(CO)Cc2cc(OC)c(O)cc2)cc1 |

**Table S4**. Example structure library for poplar lignin. The counts of monomers, linkages and functional groups, branching coefficient, and the smiles strings of the structures are shown.

|  | H | G | S | 4-O-5 | alpha-O-4 | beta-O-4 | 5-5 | beta-5 | beta-beta | beta-1 | branching_coeff | MW | monomer_count | OH_count | OCH3_count | smiles |
| --- | --- | --- | --- | --- | --- | --- | --- | --- | --- | --- | --- | --- | --- | --- | --- | --- |
| **0** | 0 | 1 | 7 | 0 | 0 | 6 | 0 | 0 | 1 | 0 | 0 | 1650 | 8 | 8 | 15 | OCC(Oc1c(OC)cc(CC(Oc2c(OC)cc(C3C4COC(C4CO3)c3cc(OC)c(OC(CO)Cc4cc(OC)c(OC(CO)Cc5cc(OC)c(OC(CO)Cc6cc(OC)c(O)cc6)c(OC)c5)c(OC)c4)c(OC)c3)cc2OC)CO)cc1OC)Cc1cc(OC)c(OC(CO)Cc2cc(OC)c(O)c(OC)c2)c(OC)c1 |
| **1** | 0 | 2 | 1 | 0 | 0 | 3 | 0 | 0 | 0 | 0 | 0 | 571 | 3 | 3 | 4 | OCC1Cc2cc(OC)c(OC(CO)Cc3cc(OC)c(OC(CO)Cc4cc(OC)c(O1)cc4)cc3)c(OC)c2 |
| **2** | 0 | 2 | 2 | 0 | 0 | 2 | 1 | 0 | 1 | 0 | 0 | 777 | 4 | 4 | 6 | OCC1Cc2cc(OC)c(OC(CO)Cc3cc(OC)c(O)c(c4c(O)c(OC)cc(C5OCC6C(OCC65)c5cc(OC)c(O1)c(OC)c5)c4)c3)c(OC)c2 |
| **3** | 0 | 2 | 1 | 0 | 0 | 2 | 1 | 0 | 0 | 0 | 0 | 569 | 3 | 3 | 4 | OCC1Cc2cc(OC)c(OC(CO)Cc3cc(OC)c4c(c5c(O1)c(OC)cc(CC(CO)O4)c5)c3)c(OC)c2 |
| **4** | 0 | 1 | 5 | 0 | 0 | 4 | 0 | 0 | 1 | 0 | 0 | 1229 | 6 | 6 | 11 | OCC(Oc1c(OC)cc(C2C3COC(C3CO2)c2cc(OC)c(OC(CO)Cc3cc(OC)c(O)c(OC)c3)cc2)cc1OC)Cc1cc(OC)c(OC(CO)Cc2cc(OC)c(OC(CO)Cc3cc(OC)c(O)c(OC)c3)c(OC)c2)c(OC)c1 |
| **5** | 0 | 4 | 2 | 0 | 0 | 5 | 1 | 1 | 0 | 0 | 0.3 | 1137 | 6 | 6 | 8 | OCC1Cc2cc(OC)c(OC(CO)Cc3cc(OC)c4c(C(CO)C(c5cc(OC)c(OC(CO)Cc6cc(OC)c7c(c6)c6c(O1)c(OC)cc(CC(Oc1c(OC)cc(CC(O7)CO)cc1OC)CO)c6)c(OC)c5)O4)c3)cc2 |
| **6** | 0 | 1 | 5 | 0 | 0 | 4 | 0 | 0 | 1 | 0 | 0 | 1229 | 6 | 6 | 11 | OCC(Cc1cc(OC)c(OC(CO)Cc2cc(OC)c(OC(CO)Cc3cc(OC)c(O)c(OC)c3)cc2)c(OC)c1)Oc1c(OC)cc(C2OCC3C(OCC23)c2cc(OC)c(OC(CO)Cc3cc(OC)c(O)c(OC)c3)c(OC)c2)cc1OC |
| **7** | 0 | 2 | 1 | 0 | 0 | 1 | 0 | 0 | 1 | 0 | 0 | 569 | 3 | 3 | 4 | Oc1c(OC)cc(CC(CO)Oc2c(OC)cc(C3C4COC(C4CO3)c3cc(OC)c(O)cc3)cc2)cc1OC |
| **8** | 0 | 1 | 3 | 0 | 0 | 4 | 0 | 0 | 0 | 0 | 0 | 811 | 4 | 4 | 7 | OCC1Cc2cc(OC)c(OC(CO)Cc3cc(OC)c(OC(CO)Cc4cc(OC)c(OC(CO)Cc5cc(OC)c(O1)c(OC)c5)cc4)c(OC)c3)c(OC)c2 |
| **9** | 0 | 1 | 2 | 0 | 0 | 2 | 0 | 1 | 0 | 0 | 0 | 599 | 3 | 3 | 5 | OCC1Cc2cc(OC)c3c(C(CO)C(c4cc(OC)c(OC(CO)Cc5cc(OC)c(O1)c(OC)c5)c(OC)c4)O3)c2 |
| **10** | 0 | 0 | 2 | 0 | 0 | 0 | 0 | 0 | 1 | 0 | 0 | 418 | 2 | 2 | 4 | Oc1c(OC)cc(C2C3COC(C3CO2)c2cc(OC)c(O)c(OC)c2)cc1OC |
| **11** | 0 | 2 | 2 | 0 | 0 | 4 | 0 | 0 | 0 | 0 | 0 | 781 | 4 | 4 | 6 | OCC1Cc2cc(OC)c(OC(CO)Cc3cc(OC)c(OC(CO)Cc4cc(OC)c(OC(CO)Cc5cc(OC)c(O1)c(OC)c5)cc4)cc3)c(OC)c2 |
| **12** | 0 | 5 | 1 | 0 | 0 | 4 | 2 | 0 | 1 | 0 | 0.5 | 1105 | 6 | 6 | 7 | OCC1Cc2cc(OC)c3c(c4c(OC(CO)Cc5cc(OC)c(O1)c(c1c(O)c(OC)cc(CC(CO)Oc6c(OC)cc(CC(CO)O3)cc6)c1)c5)c(OC)cc(C1C3COC(C3CO1)c1cc(OC)c(O)c(OC)c1)c4)c2 |
| **13** | 0 | 1 | 3 | 0 | 0 | 4 | 0 | 0 | 0 | 0 | 0 | 811 | 4 | 4 | 7 | OCC1Cc2cc(OC)c(OC(CO)Cc3cc(OC)c(OC(CO)Cc4cc(OC)c(OC(CO)Cc5cc(OC)c(O1)cc5)c(OC)c4)c(OC)c3)c(OC)c2 |
| **14** | 0 | 2 | 2 | 0 | 0 | 2 | 0 | 2 | 0 | 0 | 0 | 777 | 4 | 4 | 6 | OCC1C2c3cc(OC)c(OC(CO)Cc4cc(OC)c(OC(CO)Cc5cc(OC)c6c(C(CO)C(c7cc(OC)c(O2)c1c7)O6)c5)c(OC)c4)c(OC)c3 |
| **15** | 0 | 1 | 3 | 0 | 0 | 4 | 0 | 0 | 0 | 0 | 0 | 811 | 4 | 4 | 7 | OCC1Cc2cc(OC)c(OC(CO)Cc3cc(OC)c(OC(CO)Cc4cc(OC)c(OC(CO)Cc5cc(OC)c(O1)c(OC)c5)c(OC)c4)c(OC)c3)cc2 |
| **16** | 0 | 0 | 3 | 0 | 0 | 1 | 0 | 0 | 1 | 0 | 0 | 629 | 3 | 3 | 6 | Oc1c(OC)cc(CC(Oc2c(OC)cc(C3C4COC(C4CO3)c3cc(OC)c(O)c(OC)c3)cc2OC)CO)cc1OC |
| **17** | 0 | 1 | 2 | 0 | 0 | 2 | 0 | 1 | 0 | 0 | 0 | 599 | 3 | 3 | 5 | OCC1Cc2cc(OC)c3c(C(CO)C(c4cc(OC)c(OC(CO)Cc5cc(OC)c(O1)c(OC)c5)c(OC)c4)O3)c2 |
| **18** | 0 | 2 | 3 | 0 | 0 | 3 | 0 | 0 | 1 | 0 | 0 | 989 | 5 | 5 | 8 | OCC(Oc1c(OC)cc(C2C3COC(C3CO2)c2cc(OC)c(O)cc2)cc1)Cc1cc(OC)c(OC(CO)Cc2cc(OC)c(OC(CO)Cc3cc(OC)c(O)c(OC)c3)c(OC)c2)c(OC)c1 |
| **19** | 0 | 1 | 3 | 0 | 0 | 4 | 0 | 0 | 0 | 0 | 0 | 811 | 4 | 4 | 7 | OCC1Cc2cc(OC)c(OC(CO)Cc3cc(OC)c(OC(CO)Cc4cc(OC)c(OC(CO)Cc5cc(OC)c(O1)c(OC)c5)c(OC)c4)c(OC)c3)cc2 |
| **20** | 0 | 2 | 2 | 0 | 0 | 3 | 1 | 0 | 0 | 0 | 0 | 779 | 4 | 4 | 6 | OCC1Cc2cc(OC)c(OC(CO)Cc3cc(OC)c4c(c5c(OC(CO)Cc6cc(OC)c(O1)c(OC)c6)c(OC)cc(CC(CO)O4)c5)c3)c(OC)c2 |
| **21** | 0 | 1 | 3 | 0 | 0 | 4 | 0 | 0 | 0 | 0 | 0 | 811 | 4 | 4 | 7 | OCC1Cc2cc(OC)c(OC(CO)Cc3cc(OC)c(OC(CO)Cc4cc(OC)c(OC(CO)Cc5cc(OC)c(O1)cc5)c(OC)c4)c(OC)c3)c(OC)c2 |
| **22** | 0 | 3 | 4 | 0 | 0 | 5 | 0 | 0 | 1 | 0 | 0 | 1380 | 7 | 7 | 11 | OCC(Oc1c(OC)cc(CC(Oc2c(OC)cc(CC(Oc3c(OC)cc(C4C5COC(C5CO4)c4cc(OC)c(O)cc4)cc3OC)CO)cc2OC)CO)cc1)Cc1cc(OC)c(OC(CO)Cc2cc(OC)c(OC(CO)Cc3cc(OC)c(O)c(OC)c3)c(OC)c2)cc1 |
| **23** | 0 | 1 | 3 | 0 | 0 | 4 | 0 | 0 | 0 | 0 | 0 | 811 | 4 | 4 | 7 | OCC1Cc2cc(OC)c(OC(CO)Cc3cc(OC)c(OC(CO)Cc4cc(OC)c(OC(CO)Cc5cc(OC)c(O1)cc5)c(OC)c4)c(OC)c3)c(OC)c2 |
| **24** | 0 | 2 | 1 | 0 | 0 | 3 | 0 | 0 | 0 | 0 | 0 | 571 | 3 | 3 | 4 | OCC1Cc2cc(OC)c(OC(CO)Cc3cc(OC)c(OC(CO)Cc4cc(OC)c(O1)cc4)cc3)c(OC)c2 |
| **25** | 0 | 1 | 5 | 0 | 0 | 4 | 0 | 0 | 1 | 0 | 0 | 1229 | 6 | 6 | 11 | Oc1c(OC)cc(CC(Oc2c(OC)cc(CC(Oc3c(OC)cc(C4C5COC(C5CO4)c4cc(OC)c(OC(CO)Cc5cc(OC)c(OC(CO)Cc6cc(OC)c(O)c(OC)c6)c(OC)c5)c(OC)c4)cc3OC)CO)cc2OC)CO)cc1 |
| **26** | 0 | 1 | 5 | 0 | 0 | 3 | 0 | 1 | 1 | 0 | 0 | 1227 | 6 | 6 | 11 | OCC(Cc1cc(OC)c(OC(CO)Cc2cc(OC)c3c(C(CO)C(c4cc(OC)c(O)c(OC)c4)O3)c2)c(OC)c1)Oc1c(OC)cc(C2OCC3C(OCC23)c2cc(OC)c(OC(CO)Cc3cc(OC)c(O)c(OC)c3)c(OC)c2)cc1OC |
| **27** | 0 | 2 | 2 | 0 | 0 | 3 | 1 | 0 | 0 | 0 | 0 | 779 | 4 | 4 | 6 | OCC1Cc2cc(OC)c(OC(CO)Cc3cc(OC)c4c(c5c(OC(CO)Cc6cc(OC)c(O1)c(OC)c6)c(OC)cc(CC(CO)O4)c5)c3)c(OC)c2 |
| **28** | 0 | 0 | 3 | 0 | 0 | 1 | 0 | 0 | 1 | 0 | 0 | 629 | 3 | 3 | 6 | Oc1c(OC)cc(CC(Oc2c(OC)cc(C3C4COC(C4CO3)c3cc(OC)c(O)c(OC)c3)cc2OC)CO)cc1OC |
| **29** | 0 | 1 | 2 | 0 | 0 | 1 | 0 | 0 | 1 | 0 | 0 | 599 | 3 | 3 | 5 | Oc1c(OC)cc(CC(Oc2c(OC)cc(C3C4COC(C4CO3)c3cc(OC)c(O)c(OC)c3)cc2)CO)cc1OC |
| **30** | 0 | 1 | 2 | 0 | 0 | 1 | 0 | 0 | 1 | 0 | 0 | 599 | 3 | 3 | 5 | Oc1c(OC)cc(CC(Oc2c(OC)cc(C3C4COC(C4CO3)c3cc(OC)c(O)c(OC)c3)cc2)CO)cc1OC |
| **31** | 0 | 3 | 2 | 0 | 0 | 2 | 1 | 0 | 1 | 0 | 0 | 957 | 5 | 5 | 7 | Oc1c(OC)cc(CC(CO)Oc2c(OC)cc(C3C4COC(C4CO3)c3cc(OC)c4c(c5c(OC(CO)Cc6cc(OC)c(O)cc6)c(OC)cc(CC(CO)O4)c5)c3)cc2OC)cc1OC |
| **32** | 0 | 2 | 2 | 0 | 0 | 3 | 1 | 0 | 0 | 0 | 0 | 779 | 4 | 4 | 6 | OCC1Cc2cc(OC)c(OC(CO)Cc3cc(OC)c(OC(CO)Cc4cc(OC)c5c(c4)c4c(O1)c(OC)cc(CC(O5)CO)c4)c(OC)c3)c(OC)c2 |
| **33** | 0 | 2 | 3 | 0 | 0 | 2 | 1 | 0 | 1 | 0 | 0 | 987 | 5 | 5 | 8 | OCC(Oc1c(OC)cc(C2C3COC(C3CO2)c2cc(OC)c3c(c4c(O)c(OC)cc(CC(CO)O3)c4)c2)cc1OC)Cc1cc(OC)c(OC(CO)Cc2cc(OC)c(O)c(OC)c2)c(OC)c1 |
| **34** | 0 | 1 | 5 | 0 | 0 | 4 | 0 | 0 | 1 | 0 | 0 | 1229 | 6 | 6 | 11 | OCC(Cc1cc(OC)c(OC(CO)Cc2cc(OC)c(OC(CO)Cc3cc(OC)c(O)c(OC)c3)c(OC)c2)c(OC)c1)Oc1c(OC)cc(C2C3COC(C3CO2)c2cc(OC)c(OC(CO)Cc3cc(OC)c(O)c(OC)c3)c(OC)c2)cc1 |
| **35** | 0 | 1 | 3 | 0 | 0 | 4 | 0 | 0 | 0 | 0 | 0 | 811 | 4 | 4 | 7 | OCC1Cc2cc(OC)c(OC(CO)Cc3cc(OC)c(OC(CO)Cc4cc(OC)c(OC(CO)Cc5cc(OC)c(O1)c(OC)c5)c(OC)c4)c(OC)c3)cc2 |
| **36** | 0 | 2 | 1 | 0 | 0 | 3 | 0 | 0 | 0 | 0 | 0 | 571 | 3 | 3 | 4 | OCC1Cc2cc(OC)c(OC(CO)Cc3cc(OC)c(OC(CO)Cc4cc(OC)c(O1)cc4)cc3)c(OC)c2 |
| **37** | 0 | 3 | 6 | 0 | 0 | 7 | 0 | 0 | 1 | 0 | 0 | 1800 | 9 | 9 | 15 | OCC(Oc1c(OC)cc(C2C3COC(C3CO2)c2cc(OC)c(OC(CO)Cc3cc(OC)c(OC(CO)Cc4cc(OC)c(O)c(OC)c4)cc3)c(OC)c2)cc1OC)Cc1cc(OC)c(OC(CO)Cc2cc(OC)c(OC(CO)Cc3cc(OC)c(OC(CO)Cc4cc(OC)c(OC(CO)Cc5cc(OC)c(O)c(OC)c5)c(OC)c4)c(OC)c3)cc2)cc1 |
| **38** | 0 | 2 | 2 | 0 | 0 | 4 | 0 | 0 | 0 | 0 | 0 | 781 | 4 | 4 | 6 | OCC1Cc2cc(OC)c(OC(CO)Cc3cc(OC)c(OC(CO)Cc4cc(OC)c(OC(CO)Cc5cc(OC)c(O1)cc5)c(OC)c4)cc3)c(OC)c2 |
| **39** | 0 | 3 | 5 | 0 | 0 | 5 | 0 | 1 | 1 | 0 | 0 | 1588 | 8 | 8 | 13 | OCC(Oc1c(OC)cc(C2C3COC(C3CO2)c2cc(OC)c3c(C(CO)C(c4cc(OC)c(OC(CO)Cc5cc(OC)c(O)cc5)cc4)O3)c2)cc1OC)Cc1cc(OC)c(OC(CO)Cc2cc(OC)c(OC(CO)Cc3cc(OC)c(OC(CO)Cc4cc(OC)c(O)c(OC)c4)c(OC)c3)c(OC)c2)c(OC)c1 |
| **40** | 0 | 1 | 2 | 0 | 0 | 2 | 0 | 1 | 0 | 0 | 0 | 599 | 3 | 3 | 5 | OCC1Cc2cc(OC)c(OC(CO)Cc3cc(OC)c4c(C(CO)C(c5cc(OC)c(O1)c(OC)c5)O4)c3)c(OC)c2 |
| **41** | 0 | 2 | 1 | 0 | 0 | 2 | 1 | 0 | 0 | 0 | 0 | 569 | 3 | 3 | 4 | OCC1Cc2cc(OC)c(OC(CO)Cc3cc(OC)c4c(c5c(O1)c(OC)cc(CC(CO)O4)c5)c3)c(OC)c2 |
| **42** | 0 | 2 | 2 | 0 | 0 | 3 | 0 | 1 | 0 | 0 | 0 | 779 | 4 | 4 | 6 | OCC1Cc2cc(OC)c(OC(CO)Cc3cc(OC)c4c(c3)C(CO)C(O4)c3cc(OC)c(OC(CO)Cc4cc(OC)c(O1)c(OC)c4)c(OC)c3)cc2 |
| **43** | 0 | 0 | 3 | 0 | 0 | 1 | 0 | 0 | 1 | 0 | 0 | 629 | 3 | 3 | 6 | Oc1c(OC)cc(CC(Oc2c(OC)cc(C3C4COC(C4CO3)c3cc(OC)c(O)c(OC)c3)cc2OC)CO)cc1OC |
| **44** | 0 | 4 | 1 | 0 | 0 | 3 | 0 | 0 | 1 | 0 | 0 | 929 | 5 | 5 | 6 | OCC(Cc1cc(OC)c(OC(CO)Cc2cc(OC)c(O)cc2)cc1)Oc1c(OC)cc(C2C3COC(C3CO2)c2cc(OC)c(OC(CO)Cc3cc(OC)c(O)cc3)cc2)cc1OC |
| **45** | 0 | 2 | 2 | 0 | 0 | 3 | 0 | 1 | 0 | 0 | 0 | 779 | 4 | 4 | 6 | OCC1Cc2cc(OC)c(OC(CO)Cc3cc(OC)c4c(C(CO)C(c5cc(OC)c(OC(CO)Cc6cc(OC)c(O1)c(OC)c6)cc5)O4)c3)c(OC)c2 |
| **46** | 0 | 2 | 2 | 0 | 0 | 4 | 0 | 0 | 0 | 0 | 0 | 781 | 4 | 4 | 6 | OCC1Cc2cc(OC)c(OC(CO)Cc3cc(OC)c(OC(CO)Cc4cc(OC)c(OC(CO)Cc5cc(OC)c(O1)c(OC)c5)cc4)c(OC)c3)cc2 |
| **47** | 0 | 2 | 1 | 0 | 0 | 3 | 0 | 0 | 0 | 0 | 0 | 571 | 3 | 3 | 4 | OCC1Cc2cc(OC)c(OC(CO)Cc3cc(OC)c(OC(CO)Cc4cc(OC)c(O1)cc4)cc3)c(OC)c2 |
| **48** | 0 | 1 | 3 | 0 | 0 | 4 | 0 | 0 | 0 | 0 | 0 | 811 | 4 | 4 | 7 | OCC1Cc2cc(OC)c(OC(CO)Cc3cc(OC)c(OC(CO)Cc4cc(OC)c(OC(CO)Cc5cc(OC)c(O1)c(OC)c5)c(OC)c4)cc3)c(OC)c2 |
| **49** | 0 | 1 | 5 | 0 | 0 | 4 | 0 | 0 | 1 | 0 | 0 | 1229 | 6 | 6 | 11 | OCC(Cc1cc(OC)c(OC(CO)Cc2cc(OC)c(OC(CO)Cc3cc(OC)c(OC(CO)Cc4cc(OC)c(O)c(OC)c4)c(OC)c3)c(OC)c2)c(OC)c1)Oc1c(OC)cc(C2C3COC(C3CO2)c2cc(OC)c(O)c(OC)c2)cc1 |
| **50** | 0 | 0 | 3 | 0 | 0 | 1 | 0 | 0 | 1 | 0 | 0 | 629 | 3 | 3 | 6 | Oc1c(OC)cc(CC(Oc2c(OC)cc(C3C4COC(C4CO3)c3cc(OC)c(O)c(OC)c3)cc2OC)CO)cc1OC |
| **51** | 0 | 1 | 5 | 0 | 0 | 3 | 0 | 1 | 1 | 0 | 0 | 1227 | 6 | 6 | 11 | OCC(Oc1c(OC)cc(CC(Oc2c(OC)cc(C3C4COC(C4CO3)c3cc(OC)c(O)c(OC)c3)cc2OC)CO)cc1OC)Cc1cc(OC)c2c(C(CO)C(c3cc(OC)c(OC(CO)Cc4cc(OC)c(O)c(OC)c4)c(OC)c3)O2)c1 |
| **52** | 0 | 1 | 5 | 0 | 0 | 4 | 0 | 0 | 1 | 0 | 0 | 1229 | 6 | 6 | 11 | OCC(Oc1c(OC)cc(C2C3COC(C3CO2)c2cc(OC)c(OC(CO)Cc3cc(OC)c(OC(CO)Cc4cc(OC)c(O)c(OC)c4)c(OC)c3)c(OC)c2)cc1)Cc1cc(OC)c(OC(CO)Cc2cc(OC)c(O)c(OC)c2)c(OC)c1 |
| **53** | 0 | 1 | 3 | 0 | 0 | 4 | 0 | 0 | 0 | 0 | 0 | 811 | 4 | 4 | 7 | OCC1Cc2cc(OC)c(OC(CO)Cc3cc(OC)c(OC(CO)Cc4cc(OC)c(OC(CO)Cc5cc(OC)c(O1)c(OC)c5)c(OC)c4)cc3)c(OC)c2 |
| **54** | 0 | 1 | 5 | 0 | 0 | 4 | 0 | 0 | 1 | 0 | 0 | 1229 | 6 | 6 | 11 | OCC(Cc1cc(OC)c(OC(CO)Cc2cc(OC)c(O)c(OC)c2)c(OC)c1)Oc1c(OC)cc(C2C3COC(C3CO2)c2cc(OC)c(OC(CO)Cc3cc(OC)c(OC(CO)Cc4cc(OC)c(O)c(OC)c4)cc3)c(OC)c2)cc1OC |
| **55** | 0 | 3 | 5 | 0 | 0 | 6 | 0 | 0 | 1 | 0 | 0 | 1590 | 8 | 8 | 13 | OCC(Oc1c(OC)cc(CC(Oc2c(OC)cc(CC(Oc3c(OC)cc(C4C5COC(C5CO4)c4cc(OC)c(OC(CO)Cc5cc(OC)c(O)cc5)c(OC)c4)cc3OC)CO)cc2OC)CO)cc1OC)Cc1cc(OC)c(OC(CO)Cc2cc(OC)c(OC(CO)Cc3cc(OC)c(O)c(OC)c3)cc2)cc1 |
| **56** | 0 | 4 | 4 | 0 | 0 | 6 | 0 | 0 | 1 | 0 | 0 | 1560 | 8 | 8 | 12 | OCC(Cc1cc(OC)c(OC(CO)Cc2cc(OC)c(OC(CO)Cc3cc(OC)c(OC(CO)Cc4cc(OC)c(OC(CO)Cc5cc(OC)c(O)cc5)cc4)cc3)c(OC)c2)c(OC)c1)Oc1c(OC)cc(C2C3COC(C3CO2)c2cc(OC)c(OC(CO)Cc3cc(OC)c(O)cc3)c(OC)c2)cc1OC |
| **57** | 0 | 3 | 1 | 0 | 0 | 2 | 1 | 0 | 1 | 0 | 0 | 747 | 4 | 4 | 5 | Oc1c(OC)cc2cc1c1c(O)c(OC)cc(C3OCC4C(OCC34)c3cc(OC)c(OC(CO)Cc4cc(OC)c(OC(CO)C2)cc4)c(OC)c3)c1 |
| **58** | 0 | 1 | 3 | 0 | 0 | 4 | 0 | 0 | 0 | 0 | 0 | 811 | 4 | 4 | 7 | OCC1Cc2cc(OC)c(OC(CO)Cc3cc(OC)c(OC(CO)Cc4cc(OC)c(OC(CO)Cc5cc(OC)c(O1)c(OC)c5)c(OC)c4)c(OC)c3)cc2 |
| **59** | 0 | 1 | 4 | 0 | 0 | 2 | 0 | 1 | 1 | 0 | 0 | 1017 | 5 | 5 | 9 | OCC(Cc1cc(OC)c(OC(CO)Cc2cc(OC)c(O)c(OC)c2)c(OC)c1)Oc1c(OC)cc(C2C3COC(C3CO2)c2cc(OC)c3c(C(CO)C(c4cc(OC)c(O)c(OC)c4)O3)c2)cc1OC |
| **60** | 0 | 1 | 5 | 0 | 0 | 4 | 0 | 0 | 1 | 0 | 0 | 1229 | 6 | 6 | 11 | Oc1c(OC)cc(CC(Oc2c(OC)cc(CC(Oc3c(OC)cc(C4C5COC(C5CO4)c4cc(OC)c(OC(CO)Cc5cc(OC)c(OC(CO)Cc6cc(OC)c(O)c(OC)c6)c(OC)c5)c(OC)c4)cc3)CO)cc2OC)CO)cc1OC |
| **61** | 0 | 4 | 1 | 0 | 0 | 3 | 1 | 0 | 1 | 0 | 0 | 927 | 5 | 5 | 6 | OCC1Oc2c(OC)cc(C3C4COC(C4CO3)c3cc(OC)c(OC(CO)Cc4cc(OC)c(O)c(c4)c4c(O)c(OC)cc(CC(CO)Oc5c(OC)cc(C1)cc5)c4)c(OC)c3)cc2 |
| **62** | 0 | 1 | 3 | 0 | 0 | 4 | 0 | 0 | 0 | 0 | 0 | 811 | 4 | 4 | 7 | OCC1Cc2cc(OC)c(OC(CO)Cc3cc(OC)c(OC(CO)Cc4cc(OC)c(OC(CO)Cc5cc(OC)c(O1)cc5)c(OC)c4)c(OC)c3)c(OC)c2 |
| **63** | 0 | 2 | 4 | 0 | 0 | 4 | 0 | 0 | 1 | 0 | 0 | 1199 | 6 | 6 | 10 | Oc1c(OC)cc(CC(Oc2c(OC)cc(CC(Oc3c(OC)cc(C4C5COC(C5CO4)c4cc(OC)c(OC(CO)Cc5cc(OC)c(OC(CO)Cc6cc(OC)c(O)cc6)cc5)c(OC)c4)cc3OC)CO)cc2OC)CO)cc1OC |
| **64** | 0 | 1 | 7 | 0 | 0 | 6 | 0 | 0 | 1 | 0 | 0 | 1650 | 8 | 8 | 15 | OCC(Oc1c(OC)cc(C2C3COC(C3CO2)c2cc(OC)c(OC(CO)Cc3cc(OC)c(O)c(OC)c3)c(OC)c2)cc1OC)Cc1cc(OC)c(OC(CO)Cc2cc(OC)c(OC(CO)Cc3cc(OC)c(OC(CO)Cc4cc(OC)c(OC(CO)Cc5cc(OC)c(O)cc5)c(OC)c4)c(OC)c3)c(OC)c2)c(OC)c1 |
| **65** | 0 | 4 | 5 | 0 | 0 | 8 | 0 | 1 | 0 | 0 | 0 | 1770 | 9 | 9 | 14 | OCC1Cc2cc(OC)c(OC(CO)Cc3cc(OC)c(OC(CO)Cc4cc(OC)c(OC(CO)Cc5cc(OC)c(OC(CO)Cc6cc(OC)c(OC(CO)Cc7cc(OC)c(OC(CO)Cc8cc(OC)c9c(c8)C(CO)C(O9)c8cc(OC)c(OC(CO)Cc9cc(OC)c(O1)c(OC)c9)cc8)cc7)cc6)c(OC)c5)c(OC)c4)c(OC)c3)c(OC)c2 |
| **66** | 0 | 1 | 2 | 0 | 0 | 2 | 0 | 1 | 0 | 0 | 0 | 599 | 3 | 3 | 5 | OCC1Cc2cc(OC)c(OC(CO)Cc3cc(OC)c4c(C(CO)C(c5cc(OC)c(O1)c(OC)c5)O4)c3)c(OC)c2 |
| **67** | 0 | 2 | 2 | 0 | 0 | 2 | 0 | 0 | 1 | 0 | 0 | 779 | 4 | 4 | 6 | Oc1c(OC)cc(C2OCC3C(OCC23)c2cc(OC)c(OC(CO)Cc3cc(OC)c(OC(CO)Cc4cc(OC)c(O)cc4)cc3)c(OC)c2)cc1OC |
| **68** | 0 | 1 | 3 | 0 | 0 | 4 | 0 | 0 | 0 | 0 | 0 | 811 | 4 | 4 | 7 | OCC1Cc2cc(OC)c(OC(CO)Cc3cc(OC)c(OC(CO)Cc4cc(OC)c(OC(CO)Cc5cc(OC)c(O1)c(OC)c5)c(OC)c4)c(OC)c3)cc2 |
| **69** | 0 | 2 | 1 | 0 | 0 | 3 | 0 | 0 | 0 | 0 | 0 | 571 | 3 | 3 | 4 | OCC1Cc2cc(OC)c(OC(CO)Cc3cc(OC)c(OC(CO)Cc4cc(OC)c(O1)cc4)c(OC)c3)cc2 |
| **70** | 0 | 0 | 3 | 0 | 0 | 1 | 0 | 0 | 1 | 0 | 0 | 629 | 3 | 3 | 6 | Oc1c(OC)cc(CC(CO)Oc2c(OC)cc(C3C4COC(C4CO3)c3cc(OC)c(O)c(OC)c3)cc2OC)cc1OC |
| **71** | 0 | 1 | 3 | 0 | 0 | 3 | 0 | 1 | 0 | 0 | 0 | 809 | 4 | 4 | 7 | OCC1Cc2cc(OC)c(OC(CO)Cc3cc(OC)c4c(C(CO)C(c5cc(OC)c(OC(CO)Cc6cc(OC)c(O1)c(OC)c6)c(OC)c5)O4)c3)c(OC)c2 |
| **72** | 0 | 2 | 2 | 0 | 0 | 3 | 1 | 0 | 0 | 0 | 0 | 779 | 4 | 4 | 6 | OCC1Cc2cc(OC)c(OC(CO)Cc3cc(OC)c(OC(CO)Cc4cc(OC)c5c(c6c(O1)c(OC)cc(CC(CO)O5)c6)c4)c(OC)c3)c(OC)c2 |
| **73** | 0 | 4 | 4 | 0 | 0 | 6 | 1 | 0 | 1 | 0 | 0.3 | 1558 | 8 | 8 | 12 | OCC1Cc2cc(OC)c(OC(CO)Cc3cc(OC)c(OC(CO)Cc4cc(OC)c(OC(CO)Cc5cc(OC)c(OC(CO)Cc6cc(OC)c(O)cc6)cc5)c(c5c(OC(CO)Cc6cc(OC)c(O1)c(OC)c6)c(OC)cc(C1C6COC(C6CO1)c1cc(OC)c(O)c(OC)c1)c5)c4)c(OC)c3)c(OC)c2 |
| **74** | 0 | 0 | 8 | 0 | 0 | 6 | 0 | 0 | 1 | 0 | 0 | 1680 | 8 | 8 | 16 | OCC(Oc1c(OC)cc(C2C3COC(C3CO2)c2cc(OC)c(OC(CO)Cc3cc(OC)c(OC(CO)Cc4cc(OC)c(OC(CO)Cc5cc(OC)c(O)c(OC)c5)c(OC)c4)c(OC)c3)c(OC)c2)cc1OC)Cc1cc(OC)c(OC(CO)Cc2cc(OC)c(OC(CO)Cc3cc(OC)c(O)c(OC)c3)c(OC)c2)c(OC)c1 |
| **75** | 0 | 2 | 1 | 0 | 0 | 2 | 1 | 0 | 0 | 0 | 0 | 569 | 3 | 3 | 4 | OCC1Cc2cc(OC)c(OC(CO)Cc3cc(OC)c4c(c3)c3c(O1)c(OC)cc(CC(O4)CO)c3)c(OC)c2 |
| **76** | 0 | 3 | 3 | 0 | 0 | 4 | 0 | 0 | 1 | 0 | 0 | 1169 | 6 | 6 | 9 | Oc1c(OC)cc(CC(Oc2c(OC)cc(C3C4COC(C4CO3)c3cc(OC)c(OC(CO)Cc4cc(OC)c(OC(CO)Cc5cc(OC)c(OC(CO)Cc6cc(OC)c(O)cc6)c(OC)c5)cc4)cc3)cc2OC)CO)cc1OC |
| **77** | 0 | 1 | 5 | 0 | 0 | 6 | 0 | 0 | 0 | 0 | 0 | 1231 | 6 | 6 | 11 | OCC1Cc2cc(OC)c(OC(CO)Cc3cc(OC)c(OC(CO)Cc4cc(OC)c(OC(CO)Cc5cc(OC)c(OC(CO)Cc6cc(OC)c(OC(CO)Cc7cc(OC)c(O1)c(OC)c7)c(OC)c6)c(OC)c5)c(OC)c4)cc3)c(OC)c2 |
| **78** | 0 | 1 | 3 | 0 | 0 | 4 | 0 | 0 | 0 | 0 | 0 | 811 | 4 | 4 | 7 | OCC1Cc2cc(OC)c(OC(CO)Cc3cc(OC)c(OC(CO)Cc4cc(OC)c(OC(CO)Cc5cc(OC)c(O1)cc5)c(OC)c4)c(OC)c3)c(OC)c2 |
| **79** | 0 | 1 | 4 | 0 | 0 | 5 | 0 | 0 | 0 | 0 | 0 | 1021 | 5 | 5 | 9 | OCC1Cc2cc(OC)c(OC(CO)Cc3cc(OC)c(OC(CO)Cc4cc(OC)c(OC(CO)Cc5cc(OC)c(OC(CO)Cc6cc(OC)c(O1)c(OC)c6)c(OC)c5)cc4)c(OC)c3)c(OC)c2 |
| **80** | 0 | 1 | 5 | 0 | 0 | 4 | 0 | 0 | 1 | 0 | 0 | 1229 | 6 | 6 | 11 | Oc1c(OC)cc(C2OCC3C(OCC23)c2cc(OC)c(OC(CO)Cc3cc(OC)c(OC(CO)Cc4cc(OC)c(OC(CO)Cc5cc(OC)c(OC(CO)Cc6cc(OC)c(O)c(OC)c6)c(OC)c5)c(OC)c4)c(OC)c3)c(OC)c2)cc1 |
| **81** | 0 | 2 | 1 | 0 | 0 | 3 | 0 | 0 | 0 | 0 | 0 | 571 | 3 | 3 | 4 | OCC1Cc2cc(OC)c(OC(CO)Cc3cc(OC)c(OC(CO)Cc4cc(OC)c(O1)cc4)c(OC)c3)cc2 |
| **82** | 0 | 1 | 2 | 0 | 0 | 1 | 0 | 0 | 1 | 0 | 0 | 599 | 3 | 3 | 5 | Oc1c(OC)cc(CC(Oc2c(OC)cc(C3C4COC(C4CO3)c3cc(OC)c(O)c(OC)c3)cc2OC)CO)cc1 |
| **83** | 0 | 0 | 3 | 0 | 0 | 1 | 0 | 0 | 1 | 0 | 0 | 629 | 3 | 3 | 6 | Oc1c(OC)cc(CC(CO)Oc2c(OC)cc(C3C4COC(C4CO3)c3cc(OC)c(O)c(OC)c3)cc2OC)cc1OC |
| **84** | 0 | 1 | 3 | 0 | 0 | 4 | 0 | 0 | 0 | 0 | 0 | 811 | 4 | 4 | 7 | OCC1Cc2cc(OC)c(OC(CO)Cc3cc(OC)c(OC(CO)Cc4cc(OC)c(OC(CO)Cc5cc(OC)c(O1)c(OC)c5)c(OC)c4)cc3)c(OC)c2 |
| **85** | 0 | 5 | 3 | 0 | 0 | 5 | 1 | 1 | 1 | 0 | 0 | 1526 | 8 | 8 | 11 | OCC1C2c3cc(OC)c(OC(CO)Cc4cc(OC)c(OC(CO)Cc5cc(OC)c(OC(CO)Cc6cc(OC)c(OC(CO)Cc7cc(OC)c(O)c(c7)c7c(O)c(OC)cc(CC(CO)Oc8c(OC)cc(C9OCC%10C(OCC%109)c9cc(OC)c(O2)c1c9)cc8OC)c7)c(OC)c6)c(OC)c5)cc4)cc3 |
| **86** | 0 | 2 | 2 | 0 | 0 | 3 | 1 | 0 | 0 | 0 | 0 | 779 | 4 | 4 | 6 | OCC1Cc2cc(OC)c(OC(CO)Cc3cc(OC)c(OC(CO)Cc4cc(OC)c5c(c6c(O1)c(OC)cc(CC(CO)O5)c6)c4)c(OC)c3)c(OC)c2 |
| **87** | 0 | 2 | 1 | 0 | 0 | 3 | 0 | 0 | 0 | 0 | 0 | 571 | 3 | 3 | 4 | OCC1Cc2cc(OC)c(OC(CO)Cc3cc(OC)c(OC(CO)Cc4cc(OC)c(O1)c(OC)c4)cc3)cc2 |
| **88** | 0 | 3 | 5 | 0 | 0 | 7 | 0 | 1 | 0 | 0 | 0 | 1590 | 8 | 8 | 13 | OCC1Cc2cc(OC)c(OC(CO)Cc3cc(OC)c(OC(CO)Cc4cc(OC)c(OC(CO)Cc5cc(OC)c(OC(CO)Cc6cc(OC)c7c(c6)C(CO)C(O7)c6cc(OC)c(OC(CO)Cc7cc(OC)c(OC(CO)Cc8cc(OC)c(O1)c(OC)c8)c(OC)c7)cc6)cc5)c(OC)c4)c(OC)c3)c(OC)c2 |
| **89** | 0 | 3 | 3 | 0 | 0 | 4 | 1 | 0 | 1 | 0 | 0.2 | 1167 | 6 | 6 | 9 | Oc1c(OC)cc2cc1c1c(OC(CO)Cc3cc(OC)c(O)cc3)c(OC)cc(CC(CO)Oc3c(OC)cc(CC(CO)Oc4c(OC)cc(CC(CO)Oc5c(OC)cc(C6OCC7C2OCC76)cc5OC)cc4OC)cc3OC)c1 |
| **90** | 0 | 2 | 1 | 0 | 0 | 2 | 1 | 0 | 0 | 0 | 0 | 569 | 3 | 3 | 4 | OCC1Cc2cc(OC)c3c(c4c(OC(CO)Cc5cc(OC)c(O1)c(OC)c5)c(OC)cc(CC(CO)O3)c4)c2 |
| **91** | 0 | 4 | 1 | 0 | 0 | 5 | 0 | 0 | 0 | 0 | 0 | 931 | 5 | 5 | 6 | OCC1Cc2cc(OC)c(OC(CO)Cc3cc(OC)c(OC(CO)Cc4cc(OC)c(OC(CO)Cc5cc(OC)c(OC(CO)Cc6cc(OC)c(O1)cc6)cc5)c(OC)c4)cc3)cc2 |
| **92** | 0 | 4 | 2 | 0 | 0 | 4 | 0 | 0 | 1 | 0 | 0 | 1139 | 6 | 6 | 8 | OCC(Oc1c(OC)cc(C2C3COC(C3CO2)c2cc(OC)c(OC(CO)Cc3cc(OC)c(O)c(OC)c3)c(OC)c2)cc1)Cc1cc(OC)c(OC(CO)Cc2cc(OC)c(OC(CO)Cc3cc(OC)c(O)cc3)cc2)cc1 |
| **93** | 0 | 3 | 2 | 0 | 0 | 4 | 0 | 1 | 0 | 0 | 0 | 959 | 5 | 5 | 7 | OCC1C2c3cc(OC)c(OC(CO)Cc4cc(OC)c(OC(CO)Cc5cc(OC)c(OC(CO)Cc6cc(OC)c(OC(CO)Cc7cc(OC)c(O2)c1c7)c(OC)c6)cc5)c(OC)c4)cc3 |
| **94** | 0 | 2 | 2 | 0 | 0 | 4 | 0 | 0 | 0 | 0 | 0 | 781 | 4 | 4 | 6 | OCC1Cc2cc(OC)c(OC(CO)Cc3cc(OC)c(OC(CO)Cc4cc(OC)c(OC(CO)Cc5cc(OC)c(O1)cc5)c(OC)c4)c(OC)c3)cc2 |
| **95** | 0 | 1 | 4 | 0 | 0 | 3 | 0 | 0 | 1 | 0 | 0 | 1019 | 5 | 5 | 9 | OCC(Cc1cc(OC)c(OC(CO)Cc2cc(OC)c(O)c(OC)c2)c(OC)c1)Oc1c(OC)cc(C2OCC3C(OCC23)c2cc(OC)c(OC(CO)Cc3cc(OC)c(O)c(OC)c3)c(OC)c2)cc1 |
| **96** | 0 | 1 | 3 | 0 | 0 | 4 | 0 | 0 | 0 | 0 | 0 | 811 | 4 | 4 | 7 | OCC1Cc2cc(OC)c(OC(CO)Cc3cc(OC)c(OC(CO)Cc4cc(OC)c(OC(CO)Cc5cc(OC)c(O1)c(OC)c5)cc4)c(OC)c3)c(OC)c2 |
| **97** | 0 | 1 | 2 | 0 | 0 | 1 | 0 | 0 | 1 | 0 | 0 | 599 | 3 | 3 | 5 | Oc1c(OC)cc(CC(Oc2c(OC)cc(C3C4COC(C4CO3)c3cc(OC)c(O)c(OC)c3)cc2)CO)cc1OC |
| **98** | 0 | 3 | 2 | 0 | 0 | 3 | 0 | 0 | 1 | 0 | 0 | 959 | 5 | 5 | 7 | OCC(Oc1c(OC)cc(CC(Oc2c(OC)cc(C3C4COC(C4CO3)c3cc(OC)c(O)c(OC)c3)cc2OC)CO)cc1)Cc1cc(OC)c(OC(CO)Cc2cc(OC)c(O)cc2)cc1 |
| **99** | 0 | 2 | 3 | 0 | 0 | 3 | 1 | 0 | 1 | 0 | 0 | 987 | 5 | 5 | 8 | OCC1Oc2c(OC)cc(C3C4COC(C4CO3)c3cc(OC)c(OC(CO)Cc4cc(OC)c(O)c(c4)c4c(O)c(OC)cc(CC(CO)Oc5c(OC)cc(C1)cc5OC)c4)c(OC)c3)cc2OC |
| **100** | 0 | 2 | 1 | 0 | 0 | 3 | 0 | 0 | 0 | 0 | 0 | 571 | 3 | 3 | 4 | OCC1Cc2cc(OC)c(OC(CO)Cc3cc(OC)c(OC(CO)Cc4cc(OC)c(O1)cc4)c(OC)c3)cc2 |

**Table S5**. Example structure library for miscanthus lignin. The counts of monomers, linkages and functional groups, branching coefficient, and the smiles strings of the structures are shown.

|  | **H** | **G** | **S** | **4-O-5** | **alpha-O-4** | **beta-O-4** | **5-5** | **beta-5** | **beta-beta** | **beta-1** | **branching_coeff** | **MW** | **monomer_count** | **OH_count** | **OCH3_count** | **smiles** |
| --- | --- | --- | --- | --- | --- | --- | --- | --- | --- | --- | --- | --- | --- | --- | --- | --- |
| **0** | 0 | 2 | 4 | 0 | 0 | 4 | 0 | 0 | 1 | 0 | 0 | 1199 | 6 | 6 | 10 | OCC(Oc1c(OC)cc(C2C3COC(C3CO2)c2cc(OC)c(OC(CO)Cc3cc(OC)c(O)c(OC)c3)c(OC)c2)cc1)Cc1cc(OC)c(OC(CO)Cc2cc(OC)c(OC(CO)Cc3cc(OC)c(O)c(OC)c3)cc2)c(OC)c1 |
| **1** | 1 | 6 | 1 | 0 | 0 | 5 | 0 | 2 | 0 | 0 | 0 | 1438 | 8 | 9 | 8 | OCC(Oc1c(OC)cc(C=CCO)cc1)Cc1cc(OC)c2c(C(CO)C(c3ccc(OC(CO)Cc4cc(OC)c(OC(CO)Cc5cc(OC)c6c(C(CO)C(c7cc(OC)c(OC(CO)Cc8cc(OC)c(OC(CO)Cc9cc(OC)c(O)cc9)c(OC)c8)cc7)O6)c5)cc4)cc3)O2)c1 |
| **2** | 0 | 2 | 2 | 0 | 0 | 1 | 0 | 1 | 1 | 0 | 0 | 777 | 4 | 4 | 6 | Oc1c(OC)cc(C2C(c3c(O2)c(OC)cc(CC(Oc2c(OC)cc(C4C5COC(C5CO4)c4cc(OC)c(O)cc4)cc2OC)CO)c3)CO)cc1OC |
| **3** | 0 | 2 | 5 | 0 | 0 | 4 | 0 | 1 | 1 | 0 | 0 | 1408 | 7 | 7 | 12 | OCC(Oc1c(OC)cc(CC(Oc2c(OC)cc(C3C4COC(C4CO3)c3cc(OC)c4c(C(CO)C(c5cc(OC)c(O)c(OC)c5)O4)c3)cc2OC)CO)cc1OC)Cc1cc(OC)c(OC(CO)Cc2cc(OC)c(OC(CO)Cc3cc(OC)c(O)c(OC)c3)cc2)c(OC)c1 |
| **4** | 0 | 4 | 3 | 0 | 0 | 4 | 0 | 1 | 1 | 0 | 0 | 1347 | 7 | 7 | 10 | OCC(Cc1cc(OC)c(OC(CO)Cc2cc(OC)c3c(C(CO)C(c4cc(OC)c(O)c(OC)c4)O3)c2)c(OC)c1)Oc1c(OC)cc(C2OCC3C(OCC32)c2cc(OC)c(OC(CO)Cc3cc(OC)c(OC(CO)Cc4cc(OC)c(O)c(OC)c4)cc3)cc2)cc1 |
| **5** | 0 | 2 | 3 | 0 | 0 | 4 | 0 | 0 | 0 | 0 | 0 | 991 | 5 | 6 | 8 | OCC(Oc1c(OC)cc(CC(Oc2c(OC)cc(C=CCO)cc2OC)CO)cc1)Cc1cc(OC)c(OC(CO)Cc2cc(OC)c(OC(CO)Cc3cc(OC)c(O)cc3)c(OC)c2)c(OC)c1 |
| **6** | 0 | 4 | 3 | 0 | 0 | 3 | 0 | 3 | 0 | 0 | 0 | 1345 | 7 | 8 | 10 | OCC1C(Oc2c(OC)cc(CC(Oc3c(OC)cc(C4C(c5c(O4)c(OC)cc(C=CCO)c5)CO)cc3)CO)cc21)c1cc(OC)c2c(C(CO)C(c3cc(OC)c(OC(CO)Cc4cc(OC)c(OC(CO)Cc5cc(OC)c(O)c(OC)c5)c(OC)c4)c(OC)c3)O2)c1 |
| **7** | 0 | 2 | 4 | 0 | 0 | 4 | 0 | 0 | 1 | 0 | 0 | 1199 | 6 | 6 | 10 | OCC(Cc1cc(OC)c(OC(CO)Cc2cc(OC)c(OC(CO)Cc3cc(OC)c(O)c(OC)c3)cc2)cc1)Oc1c(OC)cc(C2OCC3C(OCC23)c2cc(OC)c(OC(CO)Cc3cc(OC)c(O)c(OC)c3)c(OC)c2)cc1OC |
| **8** | 0 | 3 | 2 | 0 | 0 | 2 | 0 | 2 | 0 | 0 | 0 | 957 | 5 | 6 | 7 | OCC(Oc1c(OC)cc(C2C(c3c(O2)c(OC)cc(C2C(c4c(O2)c(OC)cc(C=CCO)c4)CO)c3)CO)cc1OC)Cc1cc(OC)c(OC(CO)Cc2cc(OC)c(O)cc2)c(OC)c1 |
| **9** | 1 | 3 | 1 | 0 | 0 | 3 | 0 | 1 | 0 | 0 | 0 | 899 | 5 | 6 | 5 | OCC1C(Oc2c(OC)cc(C=CCO)cc12)c1cc(OC)c(OC(CO)Cc2cc(OC)c(OC(CO)Cc3ccc(OC(CO)Cc4cc(OC)c(O)c(OC)c4)cc3)cc2)cc1 |
| **10** | 1 | 2 | 1 | 0 | 0 | 1 | 0 | 1 | 1 | 0 | 0 | 717 | 4 | 4 | 4 | OCC(Oc1ccc(C2C3COC(C3CO2)c2cc(OC)c(O)c(OC)c2)cc1)Cc1cc(OC)c2c(C(CO)C(c3cc(OC)c(O)cc3)O2)c1 |
| **11** | 0 | 1 | 4 | 0 | 0 | 3 | 0 | 0 | 1 | 0 | 0 | 1019 | 5 | 5 | 9 | Oc1c(OC)cc(CC(Oc2c(OC)cc(C3C4COC(C4CO3)c3cc(OC)c(OC(CO)Cc4cc(OC)c(OC(CO)Cc5cc(OC)c(O)cc5)c(OC)c4)c(OC)c3)cc2OC)CO)cc1OC |
| **12** | 0 | 2 | 5 | 0 | 0 | 5 | 0 | 0 | 1 | 0 | 0 | 1410 | 7 | 7 | 12 | OCC(Cc1cc(OC)c(OC(CO)Cc2cc(OC)c(O)c(OC)c2)cc1)Oc1c(OC)cc(C2C3COC(C3CO2)c2cc(OC)c(OC(CO)Cc3cc(OC)c(OC(CO)Cc4cc(OC)c(OC(CO)Cc5cc(OC)c(O)cc5)c(OC)c4)c(OC)c3)c(OC)c2)cc1OC |
| **13** | 0 | 4 | 3 | 0 | 0 | 5 | 0 | 0 | 1 | 0 | 0 | 1349 | 7 | 7 | 10 | OCC(Cc1cc(OC)c(OC(CO)Cc2cc(OC)c(O)cc2)cc1)Oc1c(OC)cc(C2C3COC(C3CO2)c2cc(OC)c(OC(CO)Cc3cc(OC)c(OC(CO)Cc4cc(OC)c(OC(CO)Cc5cc(OC)c(O)cc5)c(OC)c4)cc3)c(OC)c2)cc1OC |
| **14** | 0 | 5 | 3 | 0 | 0 | 5 | 0 | 1 | 1 | 0 | 0 | 1528 | 8 | 8 | 11 | OCC(Oc1c(OC)cc(CC(Oc2c(OC)cc(C3C(c4c(O3)c(OC)cc(C3C5COC(C5CO3)c3cc(OC)c(O)cc3)c4)CO)cc2)CO)cc1OC)Cc1cc(OC)c(OC(CO)Cc2cc(OC)c(OC(CO)Cc3cc(OC)c(OC(CO)Cc4cc(OC)c(O)cc4)c(OC)c3)c(OC)c2)cc1 |
| **15** | 1 | 3 | 4 | 0 | 0 | 6 | 0 | 0 | 1 | 0 | 0 | 1530 | 8 | 8 | 11 | OCC(Oc1ccc(CC(Oc2c(OC)cc(CC(Oc3c(OC)cc(C4C5COC(C5CO4)c4cc(OC)c(O)c(OC)c4)cc3)CO)cc2OC)CO)cc1)Cc1cc(OC)c(OC(CO)Cc2cc(OC)c(OC(CO)Cc3cc(OC)c(OC(CO)Cc4cc(OC)c(O)c(OC)c4)c(OC)c3)cc2)cc1 |
| **16** | 0 | 3 | 4 | 0 | 0 | 4 | 0 | 1 | 1 | 0 | 0 | 1377 | 7 | 7 | 11 | OCC(Oc1c(OC)cc(C2C3COC(C3CO2)c2cc(OC)c3c(C(CO)C(c4cc(OC)c(O)cc4)O3)c2)cc1OC)Cc1cc(OC)c(OC(CO)Cc2cc(OC)c(OC(CO)Cc3cc(OC)c(OC(CO)Cc4cc(OC)c(O)cc4)c(OC)c3)c(OC)c2)c(OC)c1 |
| **17** | 1 | 4 | 2 | 0 | 0 | 3 | 0 | 2 | 1 | 0 | 0 | 1285 | 7 | 7 | 8 | OCC1C(Oc2c(OC)cc(C3C4COC(C4CO3)c3ccc4c(C(CO)C(c5cc(OC)c(O)c(OC)c5)O4)c3)cc21)c1cc(OC)c(OC(CO)Cc2cc(OC)c(OC(CO)Cc3cc(OC)c(OC(CO)Cc4cc(OC)c(O)cc4)c(OC)c3)cc2)cc1 |
| **18** | 0 | 1 | 4 | 0 | 0 | 2 | 0 | 1 | 1 | 0 | 0 | 1017 | 5 | 5 | 9 | Oc1c(OC)cc(CC(CO)Oc2c(OC)cc(C3C4COC(C4CO3)c3cc(OC)c(OC(CO)Cc4cc(OC)c5c(C(CO)C(c6cc(OC)c(O)c(OC)c6)O5)c4)c(OC)c3)cc2OC)cc1OC |
| **19** | 0 | 6 | 4 | 0 | 0 | 9 | 0 | 0 | 0 | 0 | 0 | 1922 | 10 | 11 | 14 | OCC(Oc1c(OC)cc(C=CCO)cc1)Cc1cc(OC)c(OC(CO)Cc2cc(OC)c(OC(CO)Cc3cc(OC)c(OC(CO)Cc4cc(OC)c(OC(CO)Cc5cc(OC)c(OC(CO)Cc6cc(OC)c(OC(CO)Cc7cc(OC)c(OC(CO)Cc8cc(OC)c(OC(CO)Cc9cc(OC)c(O)c(OC)c9)cc8)cc7)cc6)c(OC)c5)c(OC)c4)cc3)c(OC)c2)cc1 |
| **20** | 0 | 5 | 0 | 0 | 0 | 2 | 0 | 1 | 1 | 0 | 0 | 897 | 5 | 5 | 5 | OCC(Oc1c(OC)cc(C2C3COC(C3CO2)c2cc(OC)c(O)cc2)cc1)Cc1cc(OC)c(OC(CO)Cc2cc(OC)c3c(C(CO)C(c4cc(OC)c(O)cc4)O3)c2)cc1 |
| **21** | 0 | 2 | 2 | 0 | 0 | 1 | 0 | 1 | 1 | 0 | 0 | 777 | 4 | 4 | 6 | Oc1c(OC)cc(C2OCC3C(OCC23)c2cc(OC)c(OC(CO)Cc3cc(OC)c4c(C(CO)C(c5cc(OC)c(O)cc5)O4)c3)c(OC)c2)cc1OC |
| **22** | 0 | 4 | 5 | 0 | 0 | 5 | 0 | 2 | 1 | 0 | 0 | 1766 | 9 | 9 | 14 | OCC(Oc1c(OC)cc(C2C3COC(C3CO2)c2cc(OC)c(OC(CO)Cc3cc(OC)c(O)c(OC)c3)c(OC)c2)cc1)Cc1cc(OC)c(OC(CO)Cc2cc(OC)c3c(C(CO)C(c4cc(OC)c(OC(CO)Cc5cc(OC)c(OC(CO)Cc6cc(OC)c7c(C(CO)C(c8cc(OC)c(O)c(OC)c8)O7)c6)cc5)c(OC)c4)O3)c2)c(OC)c1 |
| **23** | 0 | 4 | 4 | 0 | 0 | 5 | 0 | 1 | 1 | 0 | 0 | 1558 | 8 | 8 | 12 | OCC(Oc1c(OC)cc(CC(Oc2c(OC)cc(C3C4COC(C4CO3)c3cc(OC)c(OC(CO)Cc4cc(OC)c(O)cc4)c(OC)c3)cc2OC)CO)cc1OC)Cc1cc(OC)c(OC(CO)Cc2cc(OC)c(OC(CO)Cc3cc(OC)c4c(C(CO)C(c5cc(OC)c(O)cc5)O4)c3)cc2)c(OC)c1 |
| **24** | 1 | 5 | 4 | 0 | 0 | 8 | 0 | 1 | 0 | 0 | 0 | 1890 | 10 | 11 | 13 | OCC(Oc1c(OC)cc(CC(Oc2c(OC)cc(CC(Oc3ccc(C=CCO)cc3)CO)cc2)CO)cc1OC)Cc1cc(OC)c2c(C(CO)C(c3cc(OC)c(OC(CO)Cc4cc(OC)c(OC(CO)Cc5cc(OC)c(OC(CO)Cc6cc(OC)c(OC(CO)Cc7cc(OC)c(OC(CO)Cc8cc(OC)c(O)cc8)cc7)c(OC)c6)c(OC)c5)cc4)c(OC)c3)O2)c1 |
| **25** | 0 | 4 | 3 | 0 | 0 | 3 | 0 | 2 | 1 | 0 | 0 | 1345 | 7 | 7 | 10 | OCC1c2c(OC1c1cc(OC)c(OC(CO)Cc3cc(OC)c(O)c(OC)c3)c(OC)c1)c(OC)cc(C1C3COC(C3CO1)c1cc(OC)c(OC(CO)Cc3cc(OC)c(OC(CO)Cc4cc(OC)c5c(C(CO)C(c6cc(OC)c(O)cc6)O5)c4)c(OC)c3)cc1)c2 |
| **26** | 0 | 7 | 3 | 0 | 0 | 8 | 0 | 0 | 1 | 0 | 0 | 1890 | 10 | 10 | 13 | OCC(Oc1c(OC)cc(CC(Oc2c(OC)cc(C3C4COC(C4CO3)c3cc(OC)c(O)cc3)cc2)CO)cc1OC)Cc1cc(OC)c(OC(CO)Cc2cc(OC)c(OC(CO)Cc3cc(OC)c(OC(CO)Cc4cc(OC)c(OC(CO)Cc5cc(OC)c(OC(CO)Cc6cc(OC)c(OC(CO)Cc7cc(OC)c(O)cc7)cc6)cc5)cc4)c(OC)c3)cc2)c(OC)c1 |
| **27** | 0 | 2 | 3 | 0 | 0 | 2 | 0 | 1 | 1 | 0 | 0 | 987 | 5 | 5 | 8 | OCC(Oc1c(OC)cc(C2C3COC(C3CO2)c2cc(OC)c(O)c(OC)c2)cc1OC)Cc1cc(OC)c(OC(CO)Cc2cc(OC)c3c(C(CO)C(c4cc(OC)c(O)cc4)O3)c2)c(OC)c1 |
| **28** | 0 | 2 | 6 | 0 | 0 | 6 | 0 | 1 | 0 | 0 | 0 | 1620 | 8 | 9 | 14 | OCC(Oc1c(OC)cc(CC(Oc2c(OC)cc(C3C(c4c(O3)c(OC)cc(C=CCO)c4)CO)cc2OC)CO)cc1OC)Cc1cc(OC)c(OC(CO)Cc2cc(OC)c(OC(CO)Cc3cc(OC)c(OC(CO)Cc4cc(OC)c(OC(CO)Cc5cc(OC)c(O)c(OC)c5)cc4)c(OC)c3)c(OC)c2)c(OC)c1 |
| **29** | 0 | 4 | 3 | 0 | 0 | 4 | 0 | 1 | 1 | 0 | 0 | 1347 | 7 | 7 | 10 | OCC(Cc1cc(OC)c(OC(CO)Cc2cc(OC)c3c(C(CO)C(c4cc(OC)c(O)c(OC)c4)O3)c2)c(OC)c1)Oc1c(OC)cc(C2OCC3C(OCC32)c2cc(OC)c(OC(CO)Cc3cc(OC)c(OC(CO)Cc4cc(OC)c(O)cc4)cc3)cc2)cc1OC |
| **30** | 0 | 1 | 5 | 0 | 0 | 4 | 0 | 0 | 1 | 0 | 0 | 1229 | 6 | 6 | 11 | Oc1c(OC)cc(CC(CO)Oc2c(OC)cc(C3C4COC(C4CO3)c3cc(OC)c(OC(CO)Cc4cc(OC)c(OC(CO)Cc5cc(OC)c(OC(CO)Cc6cc(OC)c(O)c(OC)c6)c(OC)c5)cc4)c(OC)c3)cc2OC)cc1OC |
| **31** | 1 | 2 | 4 | 0 | 0 | 6 | 0 | 0 | 0 | 0 | 0 | 1351 | 7 | 8 | 10 | OCC(Oc1c(OC)cc(CC(Oc2ccc(CC(Oc3c(OC)cc(C=CCO)cc3OC)CO)cc2)CO)cc1)Cc1cc(OC)c(OC(CO)Cc2cc(OC)c(OC(CO)Cc3cc(OC)c(OC(CO)Cc4cc(OC)c(O)c(OC)c4)c(OC)c3)c(OC)c2)cc1 |
| **32** | 2 | 2 | 0 | 0 | 0 | 1 | 0 | 1 | 1 | 0 | 0 | 657 | 4 | 4 | 2 | OCC1C(Oc2c(OC)cc(C3C4COC(C4CO3)c3cc(OC)c(O)cc3)cc12)c1ccc(OC(CO)Cc2ccc(O)cc2)cc1 |
| **33** | 0 | 3 | 3 | 0 | 0 | 5 | 0 | 0 | 0 | 0 | 0 | 1171 | 6 | 7 | 9 | OCC(Oc1c(OC)cc(CC(Oc2c(OC)cc(CC(Oc3c(OC)cc(C=CCO)cc3)CO)cc2OC)CO)cc1OC)Cc1cc(OC)c(OC(CO)Cc2cc(OC)c(OC(CO)Cc3cc(OC)c(O)c(OC)c3)cc2)cc1 |
| **34** | 0 | 2 | 6 | 0 | 0 | 6 | 0 | 0 | 1 | 0 | 0 | 1620 | 8 | 8 | 14 | OCC(Oc1c(OC)cc(CC(Oc2c(OC)cc(CC(Oc3c(OC)cc(C4C5COC(C5CO4)c4cc(OC)c(O)cc4)cc3OC)CO)cc2OC)CO)cc1)Cc1cc(OC)c(OC(CO)Cc2cc(OC)c(OC(CO)Cc3cc(OC)c(OC(CO)Cc4cc(OC)c(O)c(OC)c4)c(OC)c3)c(OC)c2)c(OC)c1 |
| **35** | 0 | 2 | 4 | 0 | 0 | 4 | 0 | 1 | 0 | 0 | 0 | 1199 | 6 | 7 | 10 | Oc1c(OC)cc(CC(Oc2c(OC)cc(CC(Oc3c(OC)cc(C4C(c5c(O4)c(OC)cc(CC(Oc4c(OC)cc(CC(Oc6c(OC)cc(C=CCO)cc6OC)CO)cc4OC)CO)c5)CO)cc3OC)CO)cc2)CO)cc1OC |
| **36** | 0 | 5 | 2 | 0 | 0 | 4 | 0 | 1 | 1 | 0 | 0 | 1317 | 7 | 7 | 9 | Oc1c(OC)cc(C2OCC3C(OCC32)c2cc(OC)c3c(C(CO)C(c4cc(OC)c(OC(CO)Cc5cc(OC)c(OC(CO)Cc6cc(OC)c(OC(CO)Cc7cc(OC)c(OC(CO)Cc8cc(OC)c(O)cc8)cc7)cc6)cc5)c(OC)c4)O3)c2)cc1OC |
| **37** | 0 | 3 | 6 | 0 | 0 | 6 | 0 | 1 | 1 | 0 | 0 | 1798 | 9 | 9 | 15 | OCC(Oc1c(OC)cc(CC(Oc2c(OC)cc(CC(Oc3c(OC)cc(C4C5COC(C5CO4)c4cc(OC)c5c(C(CO)C(c6cc(OC)c(OC(CO)Cc7cc(OC)c(O)c(OC)c7)c(OC)c6)O5)c4)cc3OC)CO)cc2OC)CO)cc1OC)Cc1cc(OC)c(OC(CO)Cc2cc(OC)c(OC(CO)Cc3cc(OC)c(O)cc3)cc2)c(OC)c1 |
| **38** | 0 | 4 | 3 | 0 | 0 | 5 | 0 | 0 | 1 | 0 | 0 | 1349 | 7 | 7 | 10 | OCC(Oc1c(OC)cc(CC(Oc2c(OC)cc(C3C4COC(C4CO3)c3cc(OC)c(OC(CO)Cc4cc(OC)c(OC(CO)Cc5cc(OC)c(O)cc5)c(OC)c4)cc3)cc2)CO)cc1OC)Cc1cc(OC)c(OC(CO)Cc2cc(OC)c(O)c(OC)c2)cc1 |
| **39** | 1 | 3 | 1 | 0 | 0 | 2 | 0 | 1 | 1 | 0 | 0 | 897 | 5 | 5 | 5 | Oc1c(OC)cc(CC(CO)Oc2c(OC)cc(C3OCC4C(OCC43)c3cc(OC)c(OC(CO)Cc4cc(OC)c5c(C(CO)C(c6ccc(O)cc6)O5)c4)cc3)cc2OC)cc1 |
| **40** | 1 | 3 | 2 | 0 | 0 | 4 | 0 | 0 | 1 | 0 | 0 | 1109 | 6 | 6 | 7 | OCC(Oc1c(OC)cc(C2C3COC(C3CO2)c2cc(OC)c(O)c(OC)c2)cc1)Cc1cc(OC)c(OC(CO)Cc2cc(OC)c(OC(CO)Cc3ccc(OC(CO)Cc4cc(OC)c(O)c(OC)c4)cc3)cc2)cc1 |
| **41** | 0 | 5 | 2 | 0 | 0 | 5 | 0 | 1 | 0 | 0 | 0 | 1319 | 7 | 8 | 9 | OCC(Oc1c(OC)cc(CC(Oc2c(OC)cc(CC(Oc3c(OC)cc(CC(Oc4c(OC)cc(C=CCO)cc4)CO)cc3)CO)cc2)CO)cc1)Cc1cc(OC)c(OC(CO)Cc2cc(OC)c3c(C(CO)C(c4cc(OC)c(O)c(OC)c4)O3)c2)c(OC)c1 |
| **42** | 0 | 3 | 1 | 0 | 0 | 1 | 0 | 1 | 1 | 0 | 0 | 747 | 4 | 4 | 5 | OCC1C(Oc2c(OC)cc(C3C4COC(C4CO3)c3cc(OC)c(O)cc3)cc21)c1cc(OC)c(OC(CO)Cc2cc(OC)c(O)cc2)c(OC)c1 |
| **43** | 0 | 1 | 3 | 0 | 0 | 1 | 0 | 1 | 1 | 0 | 0 | 807 | 4 | 4 | 7 | Oc1c(OC)cc(CC(CO)Oc2c(OC)cc(C3OCC4C(OCC43)c3cc(OC)c4c(C(CO)C(c5cc(OC)c(O)c(OC)c5)O4)c3)cc2OC)cc1OC |
| **44** | 1 | 3 | 3 | 0 | 0 | 5 | 0 | 0 | 1 | 0 | 0 | 1319 | 7 | 7 | 9 | Oc1ccc(C2C3COC(C3CO2)c2cc(OC)c(OC(CO)Cc3cc(OC)c(OC(CO)Cc4cc(OC)c(OC(CO)Cc5cc(OC)c(OC(CO)Cc6cc(OC)c(OC(CO)Cc7cc(OC)c(O)cc7)cc6)c(OC)c5)c(OC)c4)c(OC)c3)cc2)cc1 |
| **45** | 0 | 3 | 4 | 0 | 0 | 4 | 0 | 1 | 1 | 0 | 0 | 1377 | 7 | 7 | 11 | OCC(Oc1c(OC)cc(C2C3COC(C3CO2)c2cc(OC)c(OC(CO)Cc3cc(OC)c(OC(CO)Cc4cc(OC)c5c(C(CO)C(c6cc(OC)c(O)cc6)O5)c4)c(OC)c3)c(OC)c2)cc1OC)Cc1cc(OC)c(OC(CO)Cc2cc(OC)c(O)c(OC)c2)cc1 |
| **46** | 0 | 7 | 2 | 0 | 0 | 6 | 0 | 1 | 1 | 0 | 0 | 1678 | 9 | 9 | 11 | OCC(Oc1c(OC)cc(C2C3COC(C3CO2)c2cc(OC)c3c(C(CO)C(c4cc(OC)c(O)c(OC)c4)O3)c2)cc1)Cc1cc(OC)c(OC(CO)Cc2cc(OC)c(OC(CO)Cc3cc(OC)c(OC(CO)Cc4cc(OC)c(OC(CO)Cc5cc(OC)c(OC(CO)Cc6cc(OC)c(O)c(OC)c6)cc5)cc4)cc3)cc2)cc1 |
| **47** | 0 | 3 | 3 | 0 | 0 | 4 | 0 | 0 | 1 | 0 | 0 | 1169 | 6 | 6 | 9 | OCC(Oc1c(OC)cc(CC(Oc2c(OC)cc(CC(Oc3c(OC)cc(C4C5COC(C5CO4)c4cc(OC)c(O)c(OC)c4)cc3)CO)cc2)CO)cc1)Cc1cc(OC)c(OC(CO)Cc2cc(OC)c(O)c(OC)c2)c(OC)c1 |
| **48** | 1 | 2 | 2 | 0 | 0 | 2 | 0 | 1 | 1 | 0 | 0 | 927 | 5 | 5 | 6 | OCC(Oc1c(OC)cc(CC(Oc2ccc(C3C4COC(C4CO3)c3cc(OC)c(O)c(OC)c3)cc2)CO)cc1)Cc1cc(OC)c2c(C(CO)C(c3cc(OC)c(O)c(OC)c3)O2)c1 |
| **49** | 0 | 4 | 2 | 0 | 0 | 4 | 0 | 0 | 1 | 0 | 0 | 1139 | 6 | 6 | 8 | OCC(Cc1cc(OC)c(OC(CO)Cc2cc(OC)c(O)c(OC)c2)c(OC)c1)Oc1c(OC)cc(C2C3COC(C3CO2)c2cc(OC)c(OC(CO)Cc3cc(OC)c(OC(CO)Cc4cc(OC)c(O)cc4)cc3)cc2)cc1 |
| **50** | 0 | 4 | 3 | 0 | 0 | 5 | 0 | 1 | 0 | 0 | 0 | 1349 | 7 | 8 | 10 | OCC1C(Oc2c(OC)cc(C=CCO)cc21)c1cc(OC)c(OC(CO)Cc2cc(OC)c(OC(CO)Cc3cc(OC)c(OC(CO)Cc4cc(OC)c(OC(CO)Cc5cc(OC)c(OC(CO)Cc6cc(OC)c(O)cc6)cc5)c(OC)c4)cc3)c(OC)c2)c(OC)c1 |
| **51** | 0 | 4 | 3 | 0 | 0 | 5 | 0 | 0 | 1 | 0 | 0 | 1349 | 7 | 7 | 10 | OCC(Oc1c(OC)cc(CC(Oc2c(OC)cc(C3C4COC(C4CO3)c3cc(OC)c(O)c(OC)c3)cc2OC)CO)cc1OC)Cc1cc(OC)c(OC(CO)Cc2cc(OC)c(OC(CO)Cc3cc(OC)c(OC(CO)Cc4cc(OC)c(O)cc4)cc3)cc2)cc1 |
| **52** | 0 | 4 | 3 | 0 | 0 | 5 | 0 | 0 | 1 | 0 | 0 | 1349 | 7 | 7 | 10 | OCC(Oc1c(OC)cc(C2C3COC(C3CO2)c2cc(OC)c(O)c(OC)c2)cc1)Cc1cc(OC)c(OC(CO)Cc2cc(OC)c(OC(CO)Cc3cc(OC)c(OC(CO)Cc4cc(OC)c(OC(CO)Cc5cc(OC)c(O)cc5)c(OC)c4)c(OC)c3)cc2)cc1 |
| **53** | 0 | 2 | 6 | 0 | 0 | 7 | 0 | 0 | 0 | 0 | 0 | 1622 | 8 | 9 | 14 | OCC(Oc1c(OC)cc(CC(Oc2c(OC)cc(CC(Oc3c(OC)cc(C=CCO)cc3OC)CO)cc2)CO)cc1OC)Cc1cc(OC)c(OC(CO)Cc2cc(OC)c(OC(CO)Cc3cc(OC)c(OC(CO)Cc4cc(OC)c(OC(CO)Cc5cc(OC)c(O)c(OC)c5)c(OC)c4)cc3)c(OC)c2)c(OC)c1 |
| **54** | 1 | 5 | 4 | 0 | 0 | 6 | 0 | 2 | 1 | 0 | 0 | 1886 | 10 | 10 | 13 | OCC(Oc1c(OC)cc(CC(Oc2c(OC)cc(C3C4COC(C4CO3)c3ccc4c(C(CO)C(c5cc(OC)c(OC(CO)Cc6cc(OC)c(O)cc6)c(OC)c5)O4)c3)cc2)CO)cc1)Cc1cc(OC)c(OC(CO)Cc2cc(OC)c(OC(CO)Cc3cc(OC)c(OC(CO)Cc4cc(OC)c5c(C(CO)C(c6cc(OC)c(O)c(OC)c6)O5)c4)c(OC)c3)c(OC)c2)cc1 |
| **55** | 1 | 1 | 5 | 0 | 0 | 5 | 0 | 0 | 1 | 0 | 0 | 1380 | 7 | 7 | 11 | OCC(Oc1c(OC)cc(C2C3COC(C3CO2)c2cc(OC)c(O)c(OC)c2)cc1)Cc1ccc(OC(CO)Cc2cc(OC)c(OC(CO)Cc3cc(OC)c(OC(CO)Cc4cc(OC)c(OC(CO)Cc5cc(OC)c(O)c(OC)c5)c(OC)c4)c(OC)c3)c(OC)c2)cc1 |
| **56** | 0 | 4 | 4 | 0 | 0 | 5 | 0 | 2 | 0 | 0 | 0 | 1558 | 8 | 9 | 12 | OCC(Oc1c(OC)cc(C2C(c3c(O2)c(OC)cc(CC(Oc2c(OC)cc(C=CCO)cc2)CO)c3)CO)cc1OC)Cc1cc(OC)c(OC(CO)Cc2cc(OC)c(OC(CO)Cc3cc(OC)c4c(C(CO)C(c5cc(OC)c(OC(CO)Cc6cc(OC)c(O)c(OC)c6)c(OC)c5)O4)c3)cc2)c(OC)c1 |
| **57** | 0 | 4 | 3 | 0 | 0 | 6 | 0 | 0 | 0 | 0 | 0 | 1351 | 7 | 8 | 10 | OCC(Oc1c(OC)cc(CC(Oc2c(OC)cc(CC(Oc3c(OC)cc(CC(Oc4c(OC)cc(C=CCO)cc4)CO)cc3OC)CO)cc2)CO)cc1OC)Cc1cc(OC)c(OC(CO)Cc2cc(OC)c(OC(CO)Cc3cc(OC)c(O)c(OC)c3)cc2)cc1 |
| **58** | 0 | 3 | 2 | 0 | 0 | 3 | 0 | 1 | 0 | 0 | 0 | 959 | 5 | 6 | 7 | OCC(Oc1c(OC)cc(C=CCO)cc1OC)Cc1cc(OC)c(OC(CO)Cc2cc(OC)c(OC(CO)Cc3cc(OC)c4c(C(CO)C(c5cc(OC)c(O)c(OC)c5)O4)c3)cc2)cc1 |
| **59** | 0 | 3 | 6 | 0 | 0 | 6 | 0 | 1 | 1 | 0 | 0 | 1798 | 9 | 9 | 15 | OCC(Cc1cc(OC)c(OC(CO)Cc2cc(OC)c(O)c(OC)c2)cc1)Oc1c(OC)cc(C2OCC3C(OCC23)c2cc(OC)c(OC(CO)Cc3cc(OC)c4c(C(CO)C(c5cc(OC)c(OC(CO)Cc6cc(OC)c(OC(CO)Cc7cc(OC)c(OC(CO)Cc8cc(OC)c(O)c(OC)c8)c(OC)c7)cc6)c(OC)c5)O4)c3)c(OC)c2)cc1OC |
| **60** | 1 | 1 | 4 | 0 | 0 | 3 | 0 | 1 | 1 | 0 | 0 | 1167 | 6 | 6 | 9 | OCC1C(Oc2ccc(C3C4COC(C4CO3)c3cc(OC)c(O)c(OC)c3)cc21)c1cc(OC)c(OC(CO)Cc2cc(OC)c(OC(CO)Cc3cc(OC)c(OC(CO)Cc4cc(OC)c(O)c(OC)c4)c(OC)c3)c(OC)c2)cc1 |
| **61** | 0 | 4 | 2 | 0 | 0 | 2 | 0 | 3 | 0 | 0 | 0 | 1135 | 6 | 7 | 8 | OCC1C(Oc2c(OC)cc(CC(Oc3c(OC)cc(C4C(c5c(O4)c(OC)cc(C=CCO)c5)CO)cc3)CO)cc21)c1cc(OC)c2c(C(CO)C(c3cc(OC)c(OC(CO)Cc4cc(OC)c(O)c(OC)c4)c(OC)c3)O2)c1 |
| **62** | 0 | 5 | 4 | 0 | 0 | 6 | 0 | 1 | 1 | 0 | 0 | 1738 | 9 | 9 | 13 | OCC(Cc1cc(OC)c(OC(CO)Cc2cc(OC)c(OC(CO)Cc3cc(OC)c4c(C(CO)C(c5cc(OC)c(OC(CO)Cc6cc(OC)c(O)cc6)cc5)O4)c3)cc2)c(OC)c1)Oc1c(OC)cc(C2OCC3C(OCC23)c2cc(OC)c(OC(CO)Cc3cc(OC)c(OC(CO)Cc4cc(OC)c(O)cc4)c(OC)c3)c(OC)c2)cc1OC |
| **63** | 0 | 2 | 4 | 0 | 0 | 4 | 0 | 0 | 1 | 0 | 0 | 1199 | 6 | 6 | 10 | OCC(Oc1c(OC)cc(C2C3COC(C3CO2)c2cc(OC)c(O)c(OC)c2)cc1)Cc1cc(OC)c(OC(CO)Cc2cc(OC)c(OC(CO)Cc3cc(OC)c(OC(CO)Cc4cc(OC)c(O)c(OC)c4)c(OC)c3)c(OC)c2)cc1 |
| **64** | 0 | 3 | 4 | 0 | 0 | 5 | 0 | 1 | 0 | 0 | 0 | 1380 | 7 | 8 | 11 | OCC(Oc1c(OC)cc(CC(Oc2c(OC)cc(CC(Oc3c(OC)cc(C=CCO)cc3OC)CO)cc2OC)CO)cc1)Cc1cc(OC)c2c(C(CO)C(c3cc(OC)c(OC(CO)Cc4cc(OC)c(OC(CO)Cc5cc(OC)c(O)cc5)c(OC)c4)c(OC)c3)O2)c1 |
| **65** | 0 | 2 | 2 | 0 | 0 | 1 | 0 | 2 | 0 | 0 | 0 | 777 | 4 | 5 | 6 | OCC=Cc1cc(OC)c2c(C(CO)C(c3cc(OC)c(OC(CO)Cc4cc(OC)c5c(C(CO)C(c6cc(OC)c(O)c(OC)c6)O5)c4)c(OC)c3)O2)c1 |
| **66** | 0 | 2 | 4 | 0 | 0 | 4 | 0 | 0 | 1 | 0 | 0 | 1199 | 6 | 6 | 10 | OCC(Cc1cc(OC)c(OC(CO)Cc2cc(OC)c(OC(CO)Cc3cc(OC)c(O)cc3)cc2)c(OC)c1)Oc1c(OC)cc(C2C3COC(C3CO2)c2cc(OC)c(OC(CO)Cc3cc(OC)c(O)c(OC)c3)c(OC)c2)cc1OC |
| **67** | 0 | 3 | 1 | 0 | 0 | 1 | 0 | 1 | 1 | 0 | 0 | 747 | 4 | 4 | 5 | OCC(Oc1c(OC)cc(C2C3COC(C3CO2)c2cc(OC)c(O)c(OC)c2)cc1)Cc1cc(OC)c2c(C(CO)C(c3cc(OC)c(O)cc3)O2)c1 |
| **68** | 0 | 3 | 6 | 0 | 0 | 6 | 0 | 1 | 1 | 0 | 0 | 1798 | 9 | 9 | 15 | OCC1C(Oc2c(OC)cc(C3C4COC(C4CO3)c3cc(OC)c(OC(CO)Cc4cc(OC)c(OC(CO)Cc5cc(OC)c(O)c(OC)c5)c(OC)c4)c(OC)c3)cc21)c1cc(OC)c(OC(CO)Cc2cc(OC)c(OC(CO)Cc3cc(OC)c(OC(CO)Cc4cc(OC)c(OC(CO)Cc5cc(OC)c(O)c(OC)c5)cc4)cc3)c(OC)c2)c(OC)c1 |
| **69** | 0 | 2 | 4 | 0 | 0 | 4 | 0 | 1 | 0 | 0 | 0 | 1199 | 6 | 7 | 10 | OCC(Oc1c(OC)cc(CC(Oc2c(OC)cc(C=CCO)cc2OC)CO)cc1OC)Cc1cc(OC)c2c(C(CO)C(c3cc(OC)c(OC(CO)Cc4cc(OC)c(OC(CO)Cc5cc(OC)c(O)cc5)c(OC)c4)c(OC)c3)O2)c1 |
| **70** | 1 | 0 | 5 | 0 | 0 | 4 | 0 | 1 | 0 | 0 | 0 | 1199 | 6 | 7 | 10 | OCC(Oc1c(OC)cc(CC(Oc2c(OC)cc(C3C(c4c(O3)ccc(C=CCO)c4)CO)cc2OC)CO)cc1OC)Cc1cc(OC)c(OC(CO)Cc2cc(OC)c(OC(CO)Cc3cc(OC)c(O)c(OC)c3)c(OC)c2)c(OC)c1 |
| **71** | 0 | 2 | 2 | 0 | 0 | 1 | 0 | 2 | 0 | 0 | 0 | 777 | 4 | 5 | 6 | Oc1c(OC)cc(C2C(c3c(O2)c(OC)cc(CC(Oc2c(OC)cc(C4C(c5c(O4)c(OC)cc(C=CCO)c5)CO)cc2OC)CO)c3)CO)cc1OC |
| **72** | 0 | 3 | 4 | 0 | 0 | 5 | 0 | 0 | 1 | 0 | 0 | 1380 | 7 | 7 | 11 | OCC(Oc1c(OC)cc(C2C3COC(C3CO2)c2cc(OC)c(OC(CO)Cc3cc(OC)c(O)cc3)c(OC)c2)cc1OC)Cc1cc(OC)c(OC(CO)Cc2cc(OC)c(OC(CO)Cc3cc(OC)c(OC(CO)Cc4cc(OC)c(O)c(OC)c4)c(OC)c3)cc2)cc1 |
| **73** | 0 | 2 | 2 | 0 | 0 | 1 | 0 | 1 | 1 | 0 | 0 | 777 | 4 | 4 | 6 | OCC(Oc1c(OC)cc(C2C3COC(C3CO2)c2cc(OC)c(O)c(OC)c2)cc1OC)Cc1cc(OC)c2c(C(CO)C(c3cc(OC)c(O)cc3)O2)c1 |
| **74** | 0 | 3 | 1 | 0 | 0 | 1 | 0 | 2 | 0 | 0 | 0 | 747 | 4 | 5 | 5 | OCC1C(Oc2c(OC)cc(C=CCO)cc12)c1cc(OC)c2c(C(CO)C(c3cc(OC)c(OC(CO)Cc4cc(OC)c(O)cc4)c(OC)c3)O2)c1 |
| **75** | 0 | 7 | 4 | 0 | 0 | 9 | 0 | 0 | 1 | 0 | 0 | 2100 | 11 | 11 | 15 | OCC(Oc1c(OC)cc(CC(Oc2c(OC)cc(CC(Oc3c(OC)cc(CC(Oc4c(OC)cc(C5C6COC(C6CO5)c5cc(OC)c(OC(CO)Cc6cc(OC)c(OC(CO)Cc7cc(OC)c(O)c(OC)c7)c(OC)c6)cc5)cc4)CO)cc3)CO)cc2)CO)cc1OC)Cc1cc(OC)c(OC(CO)Cc2cc(OC)c(OC(CO)Cc3cc(OC)c(OC(CO)Cc4cc(OC)c(O)c(OC)c4)cc3)cc2)cc1 |
| **76** | 0 | 8 | 2 | 0 | 0 | 6 | 0 | 2 | 1 | 0 | 0 | 1856 | 10 | 10 | 12 | OCC(Oc1c(OC)cc(CC(Oc2c(OC)cc(CC(Oc3c(OC)cc(C4C(c5c(O4)c(OC)cc(C4C6COC(C6CO4)c4cc(OC)c6c(C(CO)C(c7cc(OC)c(O)c(OC)c7)O6)c4)c5)CO)cc3)CO)cc2)CO)cc1)Cc1cc(OC)c(OC(CO)Cc2cc(OC)c(OC(CO)Cc3cc(OC)c(OC(CO)Cc4cc(OC)c(O)cc4)cc3)cc2)c(OC)c1 |
| **77** | 0 | 3 | 4 | 0 | 0 | 5 | 0 | 0 | 1 | 0 | 0 | 1380 | 7 | 7 | 11 | OCC(Oc1c(OC)cc(CC(Oc2c(OC)cc(CC(Oc3c(OC)cc(C4C5COC(C5CO4)c4cc(OC)c(O)c(OC)c4)cc3)CO)cc2OC)CO)cc1)Cc1cc(OC)c(OC(CO)Cc2cc(OC)c(OC(CO)Cc3cc(OC)c(O)c(OC)c3)c(OC)c2)cc1 |
| **78** | 0 | 1 | 3 | 0 | 0 | 1 | 0 | 1 | 1 | 0 | 0 | 807 | 4 | 4 | 7 | Oc1c(OC)cc(C2C3COC(C3CO2)c2cc(OC)c(OC(CO)Cc3cc(OC)c4c(C(CO)C(c5cc(OC)c(O)c(OC)c5)O4)c3)c(OC)c2)cc1OC |
| **79** | 0 | 2 | 6 | 0 | 0 | 5 | 0 | 1 | 1 | 0 | 0 | 1618 | 8 | 8 | 14 | OCC(Cc1cc(OC)c(OC(CO)Cc2cc(OC)c(O)c(OC)c2)c(OC)c1)Oc1c(OC)cc(C2C3COC(C3CO2)c2cc(OC)c(OC(CO)Cc3cc(OC)c4c(C(CO)C(c5cc(OC)c(OC(CO)Cc6cc(OC)c(OC(CO)Cc7cc(OC)c(O)cc7)c(OC)c6)c(OC)c5)O4)c3)c(OC)c2)cc1OC |
| **80** | 0 | 6 | 1 | 0 | 0 | 5 | 0 | 0 | 1 | 0 | 0 | 1289 | 7 | 7 | 8 | OCC(Oc1c(OC)cc(CC(Oc2c(OC)cc(C3C4COC(C4CO3)c3cc(OC)c(O)cc3)cc2)CO)cc1)Cc1cc(OC)c(OC(CO)Cc2cc(OC)c(OC(CO)Cc3cc(OC)c(OC(CO)Cc4cc(OC)c(O)cc4)cc3)c(OC)c2)cc1 |
| **81** | 0 | 5 | 4 | 0 | 0 | 6 | 0 | 1 | 1 | 0 | 0 | 1738 | 9 | 9 | 13 | OCC(Cc1cc(OC)c(OC(CO)Cc2cc(OC)c3c(C(CO)C(c4cc(OC)c(OC(CO)Cc5cc(OC)c(OC(CO)Cc6cc(OC)c(O)c(OC)c6)c(OC)c5)cc4)O3)c2)c(OC)c1)Oc1c(OC)cc(C2C3COC(C3CO2)c2cc(OC)c(OC(CO)Cc3cc(OC)c(OC(CO)Cc4cc(OC)c(O)c(OC)c4)cc3)cc2)cc1 |
| **82** | 0 | 4 | 3 | 0 | 0 | 6 | 0 | 0 | 0 | 0 | 0 | 1351 | 7 | 8 | 10 | OCC(Oc1c(OC)cc(CC(Oc2c(OC)cc(C=CCO)cc2OC)CO)cc1)Cc1cc(OC)c(OC(CO)Cc2cc(OC)c(OC(CO)Cc3cc(OC)c(OC(CO)Cc4cc(OC)c(OC(CO)Cc5cc(OC)c(O)cc5)c(OC)c4)c(OC)c3)cc2)cc1 |
| **83** | 1 | 2 | 1 | 0 | 0 | 1 | 0 | 1 | 1 | 0 | 0 | 717 | 4 | 4 | 4 | OCC1C(Oc2c(OC)cc(C3C4COC(C4CO3)c3cc(OC)c(O)cc3)cc21)c1cc(OC)c(OC(CO)Cc2ccc(O)cc2)c(OC)c1 |
| **84** | 0 | 1 | 4 | 0 | 0 | 2 | 0 | 1 | 1 | 0 | 0 | 1017 | 5 | 5 | 9 | OCC(Cc1cc(OC)c2c(C(CO)C(c3cc(OC)c(O)c(OC)c3)O2)c1)Oc1c(OC)cc(C2C3COC(C3CO2)c2cc(OC)c(OC(CO)Cc3cc(OC)c(O)c(OC)c3)c(OC)c2)cc1OC |
| **85** | 0 | 2 | 4 | 0 | 0 | 4 | 0 | 0 | 1 | 0 | 0 | 1199 | 6 | 6 | 10 | OCC(Oc1c(OC)cc(CC(Oc2c(OC)cc(C3C4COC(C4CO3)c3cc(OC)c(O)cc3)cc2OC)CO)cc1)Cc1cc(OC)c(OC(CO)Cc2cc(OC)c(OC(CO)Cc3cc(OC)c(O)c(OC)c3)c(OC)c2)c(OC)c1 |
| **86** | 0 | 2 | 4 | 0 | 0 | 4 | 0 | 0 | 1 | 0 | 0 | 1199 | 6 | 6 | 10 | OCC(Cc1cc(OC)c(OC(CO)Cc2cc(OC)c(OC(CO)Cc3cc(OC)c(O)c(OC)c3)cc2)c(OC)c1)Oc1c(OC)cc(C2C3COC(C3CO2)c2cc(OC)c(OC(CO)Cc3cc(OC)c(O)c(OC)c3)c(OC)c2)cc1 |
| **87** | 0 | 5 | 3 | 0 | 0 | 5 | 0 | 1 | 1 | 0 | 0 | 1528 | 8 | 8 | 11 | OCC(Cc1cc(OC)c(OC(CO)Cc2cc(OC)c(O)cc2)cc1)Oc1c(OC)cc(C2C3COC(C3CO2)c2cc(OC)c(OC(CO)Cc3cc(OC)c(OC(CO)Cc4cc(OC)c5c(C(CO)C(c6cc(OC)c(OC(CO)Cc7cc(OC)c(O)cc7)c(OC)c6)O5)c4)c(OC)c3)c(OC)c2)cc1 |
| **88** | 0 | 3 | 3 | 0 | 0 | 4 | 0 | 0 | 1 | 0 | 0 | 1169 | 6 | 6 | 9 | OCC(Cc1cc(OC)c(OC(CO)Cc2cc(OC)c(OC(CO)Cc3cc(OC)c(O)cc3)c(OC)c2)c(OC)c1)Oc1c(OC)cc(C2C3COC(C3CO2)c2cc(OC)c(OC(CO)Cc3cc(OC)c(O)cc3)cc2)cc1OC |
| **89** | 0 | 5 | 1 | 0 | 0 | 4 | 0 | 1 | 0 | 0 | 0 | 1109 | 6 | 7 | 7 | OCC(Oc1c(OC)cc(CC(Oc2c(OC)cc(C=CCO)cc2)CO)cc1)Cc1cc(OC)c(OC(CO)Cc2cc(OC)c3c(C(CO)C(c4cc(OC)c(OC(CO)Cc5cc(OC)c(O)cc5)cc4)O3)c2)c(OC)c1 |
| **90** | 0 | 2 | 5 | 0 | 0 | 5 | 0 | 0 | 1 | 0 | 0 | 1410 | 7 | 7 | 12 | OCC(Oc1c(OC)cc(CC(Oc2c(OC)cc(CC(Oc3c(OC)cc(C4C5COC(C5CO4)c4cc(OC)c(O)cc4)cc3OC)CO)cc2)CO)cc1OC)Cc1cc(OC)c(OC(CO)Cc2cc(OC)c(OC(CO)Cc3cc(OC)c(O)c(OC)c3)c(OC)c2)c(OC)c1 |
| **91** | 1 | 3 | 3 | 0 | 0 | 5 | 0 | 0 | 1 | 0 | 0 | 1319 | 7 | 7 | 9 | OCC(Oc1c(OC)cc(C2C3COC(C3CO2)c2ccc(OC(CO)Cc3cc(OC)c(OC(CO)Cc4cc(OC)c(O)c(OC)c4)c(OC)c3)cc2)cc1)Cc1cc(OC)c(OC(CO)Cc2cc(OC)c(OC(CO)Cc3cc(OC)c(O)cc3)cc2)c(OC)c1 |
| **92** | 1 | 2 | 4 | 0 | 0 | 4 | 0 | 1 | 1 | 0 | 0 | 1347 | 7 | 7 | 10 | OCC(Oc1c(OC)cc(C2C3COC(C3CO2)c2cc(OC)c3c(C(CO)C(c4cc(OC)c(OC(CO)Cc5cc(OC)c(O)c(OC)c5)cc4)O3)c2)cc1OC)Cc1cc(OC)c(OC(CO)Cc2ccc(OC(CO)Cc3cc(OC)c(O)c(OC)c3)cc2)c(OC)c1 |
| **93** | 0 | 4 | 5 | 0 | 0 | 6 | 0 | 1 | 1 | 0 | 0 | 1768 | 9 | 9 | 14 | OCC(Oc1c(OC)cc(C2C(c3c(O2)c(OC)cc(CC(Oc2c(OC)cc(CC(Oc4c(OC)cc(C5C6COC(C6CO5)c5cc(OC)c(OC(CO)Cc6cc(OC)c(O)c(OC)c6)c(OC)c5)cc4OC)CO)cc2)CO)c3)CO)cc1)Cc1cc(OC)c(OC(CO)Cc2cc(OC)c(OC(CO)Cc3cc(OC)c(O)c(OC)c3)cc2)c(OC)c1 |
| **94** | 0 | 3 | 2 | 0 | 0 | 2 | 0 | 2 | 0 | 0 | 0 | 957 | 5 | 6 | 7 | OCC(Oc1c(OC)cc(C2C(c3c(O2)c(OC)cc(C=CCO)c3)CO)cc1OC)Cc1cc(OC)c(OC(CO)Cc2cc(OC)c3c(C(CO)C(c4cc(OC)c(O)c(OC)c4)O3)c2)cc1 |
| **95** | 0 | 2 | 4 | 0 | 0 | 4 | 0 | 0 | 1 | 0 | 0 | 1199 | 6 | 6 | 10 | OCC(Oc1c(OC)cc(CC(Oc2c(OC)cc(C3C4COC(C4CO3)c3cc(OC)c(O)c(OC)c3)cc2OC)CO)cc1)Cc1cc(OC)c(OC(CO)Cc2cc(OC)c(OC(CO)Cc3cc(OC)c(O)c(OC)c3)c(OC)c2)cc1 |
| **96** | 0 | 4 | 2 | 0 | 0 | 4 | 0 | 1 | 0 | 0 | 0 | 1139 | 6 | 7 | 8 | OCC(Oc1c(OC)cc(CC(Oc2c(OC)cc(C3C(c4c(O3)c(OC)cc(C=CCO)c4)CO)cc2)CO)cc1OC)Cc1cc(OC)c(OC(CO)Cc2cc(OC)c(OC(CO)Cc3cc(OC)c(O)cc3)c(OC)c2)cc1 |
| **97** | 1 | 1 | 2 | 0 | 0 | 1 | 0 | 1 | 1 | 0 | 0 | 747 | 4 | 4 | 5 | Oc1c(OC)cc(CC(CO)Oc2c(OC)cc(C3OCC4C(OCC43)c3ccc4c(C(CO)C(c5cc(OC)c(O)c(OC)c5)O4)c3)cc2)cc1OC |
| **98** | 0 | 5 | 2 | 0 | 0 | 5 | 0 | 1 | 0 | 0 | 0 | 1319 | 7 | 8 | 9 | OCC(Oc1c(OC)cc(CC(Oc2c(OC)cc(CC(Oc3c(OC)cc(C=CCO)cc3)CO)cc2OC)CO)cc1)Cc1cc(OC)c2c(C(CO)C(c3cc(OC)c(OC(CO)Cc4cc(OC)c(OC(CO)Cc5cc(OC)c(O)cc5)cc4)c(OC)c3)O2)c1 |
| **99** | 0 | 3 | 4 | 0 | 0 | 6 | 0 | 0 | 0 | 0 | 0 | 1382 | 7 | 8 | 11 | OCC(Oc1c(OC)cc(C=CCO)cc1)Cc1cc(OC)c(OC(CO)Cc2cc(OC)c(OC(CO)Cc3cc(OC)c(OC(CO)Cc4cc(OC)c(OC(CO)Cc5cc(OC)c(OC(CO)Cc6cc(OC)c(O)c(OC)c6)cc5)c(OC)c4)c(OC)c3)c(OC)c2)cc1 |
| **100** | 0 | 2 | 3 | 0 | 0 | 2 | 0 | 1 | 1 | 0 | 0 | 987 | 5 | 5 | 8 | OCC(Oc1c(OC)cc(C2C(c3c(O2)c(OC)cc(C2C4COC(C4CO2)c2cc(OC)c(O)cc2)c3)CO)cc1OC)Cc1cc(OC)c(OC(CO)Cc2cc(OC)c(O)c(OC)c2)c(OC)c1 |

**Additional Note 4 – Computational performance**

The time complexity describes the amount of computer time it takes to run an algorithm and is often a good indicator of the algorithm's performance. The focus is on how such complexity changes with increasing input size. Big O notation is commonly used to describe the upper bound on the execution time as a function of the input size ($n$), typically expressed in $O(n)$, $O(nlogn)$, $O(n^{a})$, etc.

We measure the time complexity of polymerization for the proposed algorithm. The input here is the polymer size ($n$) or the number of monomers in a molecule. To standardize the polymerization process, we simulate the growth of a lignin molecule with only G units connected via β-O-4 linkage. Unlike other linkages, such as 5-5, the β-O-4 linkage can be added repeatedly to a growing polymer without occupying all bonding C1 atoms or terminating the growth. We follow the procedure of Lignin-KMC^6^ in measuring the time complexity. We vary $n$ between 50 and 1,000 in increments of 50. For each size, we measure the CPU execution time using the build-in time package in Python on a Windows 10 laptop with an Intel(R) Core(TM) i7-8750H processor. Each run is repeated five times to compute the mean and the 95% confidence intervals shown in Figure S6. The resulting time complexity can be expressed by fitting the data to a power-law model, $t=an^{b}$. The coefficient $a$ varies across different machines, but the exponent $b$ stays constant. For LigninGraphs, $b = 2.04$; the time complexity is near quadratic (~$O(n^{2})$). For Lignin-KMC, $b = 2.54$; the time complexity is in between quadratic and cubic. If the same coefficient $a$ holds on our machines, LigninGraphs runs faster than Lignin-KMC when n is greater than or equal to 100. To put the runtime into perspective, we anticipate that LigninGraphs takes ~2.5 minutes and Lignin-KMC ~10 min when modeling the polymerization of 1,000 monomers.

**Figure S9**. Computational time complexity of polymerization. For LigninGraphs, blue dots indicate the measured mean CPU execution time for a simulation at a specific polymer size; the blue error bars indicate the 95% confidence interval of the CPU time, averaged over five runs. The blue and orange dashed lines are the power-law fits of the polymerization time complexity for LigninGraphs and Lignin-KMC, respectively.

For typical Metropolis Monte Carlo simulations, the acceptance ratio, the total MC attempts, and the runtime are largely dependent on the Metropolis temperature. Figure S10 shows an example of distance trajectories for a population and a single polymer. At low temperatures, $\Delta d$ needs to be close to zero or negative for the MC attempt to be accepted. As a result, the acceptance ratio is low, and more attempts are required to complete the simulation, but the final distance would be close to zero, indicating a good match to the target values. Users should strike a balance between runtime and accuracy. Table S6 shows the test results for acceptance ratios, total MC attempts, and runtime at varying temperatures.

**Figure S10**. Example distance trajectories in the Metropolis Monte Carlo simulations for (a) a structure population and (b) a single polymer.

**Table S6**. Acceptance ratios, total Monte Carlo (MC) attempts, and runtime for various lignin feedstocks.

| Feedstock | Temperature (K) | Acceptance Ratio | | | Total MC Attempts | Total Runtime (Minutes) |
| --- | --- | --- | --- | --- | --- | --- |
|  |  | Monomer Addition | Polymer Addition | Ring Addition |  |  |
| Miscanthus | 1 | 0.005 | 0.737 | 0.000 | 94137 | 16.45 |
|  | 5 | 0.007 | 0.893 | 0.000 | 78282 | 10.42 |
|  | 10 | 0.014 | 0.953 | 0.000 | 48463 | 5.56 |
|  | 15 | 0.047 | 1.000 | 0.000 | 16633 | 1.85 |
|  | 25 | 0.132 | 0.981 | 0.000 | 6217 | 0.72 |
|  | 50 | 0.653 | 1.000 | 0.000 | 1274 | 0.16 |
|  | 100 | 0.780 | 1.000 | 0.000 | 1120 | 0.18 |
|  | 200 | 0.941 | 1.000 | 0.000 | 924 | 0.12 |
|  | 300 | 0.983 | 1.000 | 0.000 | 884 | 0.13 |
| Pine | 1 | 0.013 | 0.470 | 0.001 | 99598 | 18.46 |
|  | 5 | 0.083 | 0.878 | 0.001 | 59088 | 2.14 |
|  | 10 | 0.095 | 0.910 | 0.001 | 58365 | 1.88 |
|  | 15 | 0.163 | 0.953 | 0.001 | 54805 | 1.36 |
|  | 25 | 0.227 | 0.935 | 0.001 | 53480 | 1.11 |
|  | 50 | 0.864 | 0.981 | 0.001 | 51037 | 0.76 |
|  | 100 | 0.922 | 1.000 | 0.001 | 50985 | 0.82 |
|  | 200 | 0.989 | 1.000 | 0.001 | 50887 | 0.88 |
|  | 300 | 0.987 | 1.000 | 0.001 | 50884 | 0.9 |
| Poplar | 1 | 0.006 | 0.445 | 0.001 | 131303 | 27.39 |
|  | 5 | 0.009 | 0.878 | 0.002 | 116743 | 10.5 |
|  | 10 | 0.014 | 0.962 | 0.002 | 97105 | 6.25 |
|  | 15 | 0.020 | 0.935 | 0.002 | 85133 | 4.75 |
|  | 25 | 0.052 | 0.971 | 0.001 | 66713 | 2.61 |
|  | 50 | 0.205 | 1.000 | 0.001 | 54046 | 1.26 |
|  | 100 | 0.406 | 1.000 | 0.002 | 52153 | 1.08 |
|  | 200 | 0.891 | 1.000 | 0.001 | 51026 | 0.95 |
|  | 300 | 0.974 | 1.000 | 0.002 | 50984 | 0.29 |

**Supplementary References**

1. Lin, T. *et al.* BigSMILES: A Structurally-Based Line Notation for Describing Macromolecules. *ACS Cent. Sci.* **5**, 1523–1531 (2019).

2. Metropolis, N., Rosenbluth, A. W., Rosenbluth, M. N., Teller, A. H. & Teller, E. Equation of state calculations by fast computing machines. *J. Chem. Phys.* **21**, 1087–1092 (1953).

3. Gelman, A., Roberts, G. O. & Gilks, W. R. Efficient Metropolis jumping rules. *Bayesian Statistics* vol. 5 599–608 (1996).

4. Moskovkin, P. & Hou, M. Metropolis Monte Carlo predictions of free Co-Pt nanoclusters. *J. Alloys Compd.* **434**–**435**, 550–554 (2007).

5. Liu, X., Seider, W. D. & Sinno, T. A general method for spatially coarse-graining Metropolis Monte Carlo simulations onto a lattice. *J. Chem. Phys.* **138**, (2013).

6. Orella, M. J. *et al.* Lignin-KMC: A Toolkit for Simulating Lignin Biosynthesis. *ACS Sustain. Chem. Eng.* **7**, 18313–18322 (2019).
